# Diosbulbin C, a novel active ingredient in *Dioscorea bulbifera* L. extract, inhibits lung cancer cell proliferation by inducing G0/G1 phase cell cycle arrest

**DOI:** 10.1186/s12906-023-04245-9

**Published:** 2023-12-04

**Authors:** Zhiyu Zhu, Yanfen Liu, Jiangping Zeng, Shuyi Ren, Lu Wei, Fei Wang, Xiaoyu Sun, Yufei Huang, Haiyang Jiang, Xinbing Sui, Weiwei Jin, Lijun Jin, Xueni Sun

**Affiliations:** 1 School of Pharmacy, Key Laboratory of Elemene Class Anti-Cancer Chinese Medicines, Engineering Laboratory of Development and Application of Traditional Chinese Medicines, Collaborative Innovation Center of Traditional Chinese Medicines of Zhejiang Province, Hangzhou Normal University, Hangzhou, 311121 Zhejiang China; 2 Department of Gastrointestinal and Pancreatic Surgery, Key Laboratory of Gastroenterology of Zhejiang Province, Zhejiang Provincial People’s Hospital, People’s Hospital of Hangzhou Medical College, Hangzhou, 310014 Zhejiang China; 3 Department of Traditional Chinese Medicine, Hangzhou Shangcheng District People’s Hospital, Hangzhou, China

**Keywords:** Diosbulbin C, Bioactive ingredient, Traditional Chinese medicine, Non-small cell lung cancer, Anticancer activity

## Abstract

**Background:**

Despite the critical progress of non-small cell lung cancer (NSCLC) therapeutic approaches, the clinical outcomes remain considerably poor. The requirement of developing novel therapeutic interventions is still urgent. In this study, we showed for the first time that diosbulbin C, a natural diterpene lactone component extracted from traditional Chinese medicine *Dioscorea bulbifera* L., possesses high anticancer activity in NSCLC.

**Methods:**

A549 and NCI-H1299 cells were used. The inhibitory effects of the diosbulbin C on NSCLC cell proliferation were evaluated using cytotoxicity, clone formation, EdU assay, and flow cytometry. Network pharmacology methods were used to explore the targets through which the diosbulbin C inhibited NSCLC cell proliferation. Molecular docking, qRT-PCR, and western blotting were used to validate the molecular targets and regulated molecules of diosbulbin C in NSCLC.

**Results:**

Diosbulbin C treatment in NSCLC cells results in a remarkable reduction in cell proliferation and induces significant G0/G1 phase cell cycle arrest. *AKT1*, *DHFR*, and *TYMS* were identified as the potential targets of diosbulbin C. Diosbulbin C may inhibit NSCLC cell proliferation by downregulating the expression/activation of AKT, DHFR, and TYMS. In addition, diosbulbin C was predicted to exhibit high drug-likeness properties with good water solubility and intestinal absorption, highlighting its potential value in the discovery and development of anti-lung cancer drugs.

**Conclusions:**

Diosbulbin C induces cell cycle arrest and inhibits the proliferation of NSCLC cells, possibly by downregulating the expression/activation of AKT, DHFR, and TYMS.

**Supplementary Information:**

The online version contains supplementary material available at 10.1186/s12906-023-04245-9.

## Introduction

Despite the decline in cancer death rate in recent years due to earlier detection and treatment advances, lung cancer (LC) still runs the leading cause of cancer-associated mortality worldwide [1]. LC is pathologically classified into small cell lung cancer (SCLC) and non-small cell lung cancer (NSCLC). The latter accounts for the large majority of all diagnosed LC cases (~ 85%) [2]. Currently, treatments of NSCLC in the clinic mainly include surgery, radio-/chemo-therapy, targeted therapy, immunotherapy, and combination therapy. Despite the fact that current treatments have shown significant help in improving the survival of patients with NSCLC, studies have shown that targeted therapy is only effective in less than 25% of patients [3]. The combination of chemotherapies also failed to prolong the overall survival of patients with metastatic NSCLC [4]. Although immunotherapy can partly compensate for the deficiency of combined chemotherapy and optimize the treatment plan of patients with metastatic NSCLC, to a certain extent, immunotherapy-related adverse reactions become an ignorable problem in clinical practice [5].

Traditional Chinese Medicine (TCM) is commonly used as an adjuvant therapy to relieve symptoms of advanced cancers, which has shown certain advantages in preventing tumor occurrence, reducing adverse side effects of chemo-/radio-therapy, improving therapeutic effects, and minimizing tumor recurrence and metastasis [6]. It opens an excellent avenue for bioactive compound exploration in anti-lung cancer drug discovery.

**Fig. 1:**
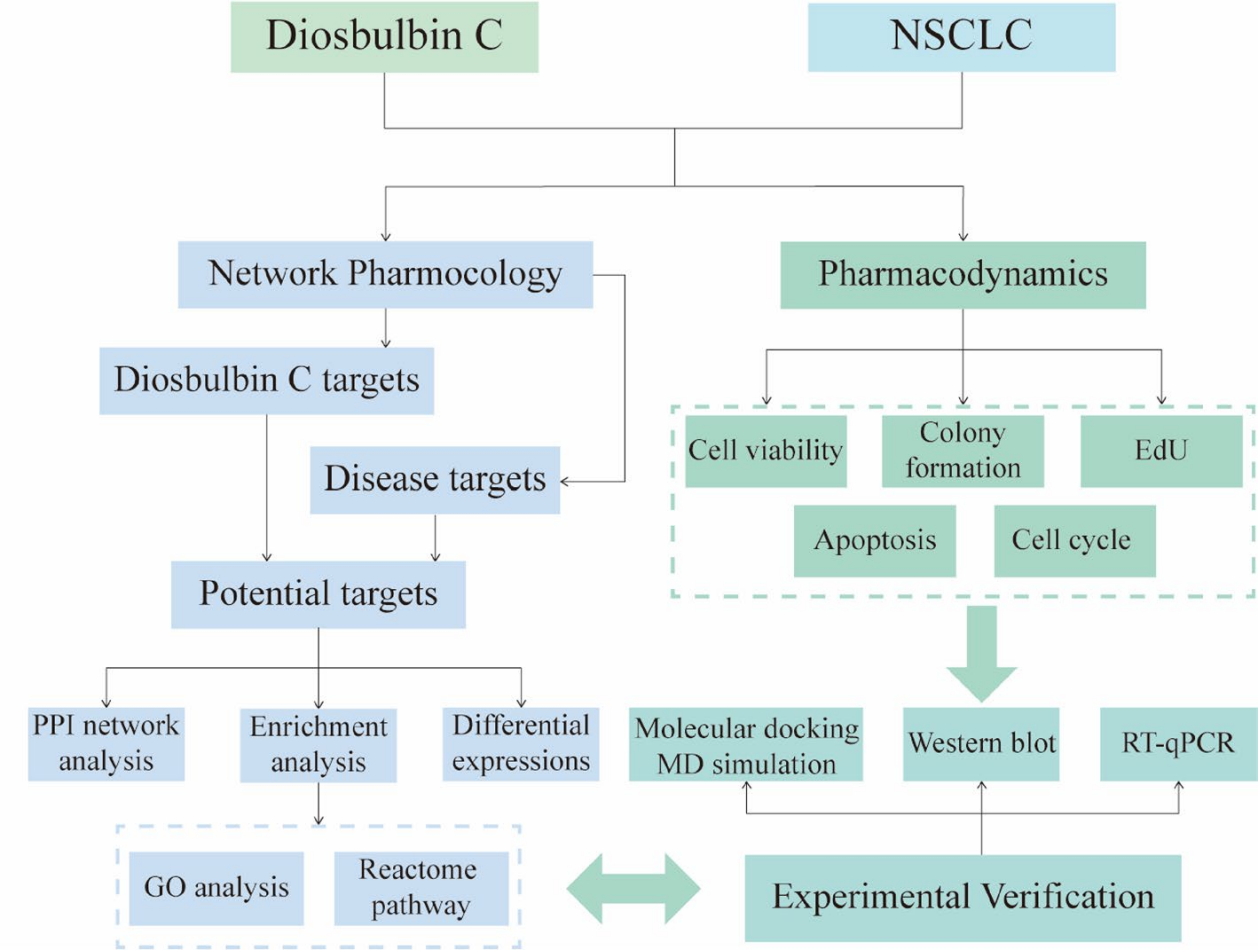
Flowchart of the network pharmacological and experimental studies of the diosbulbin C in NSCLC cells

*Dioscorea bulbifera* L. is a TCM broadly used in the clinic [7]. It has long been used to treat tumors in traditional Chinese medicine [8]. *Dioscorea bulbifera* L. contains various active ingredients, such as steroid saponins and diterpenoids [9, 10]. In particular, Diosbulbin B and C are the diterpene lactone components extracted from *Dioscorea bulbifera* L. [11], of which diosbulbin B has been reported to be an antitumor bioactive ingredient in *Dioscorea bulbifera* L. [12]. However, to the best of our knowledge, the antitumor activity of diosbulbin C has yet to be reported. In this study, we evaluated the anti-lung cancer activity of diosbulbin C with two NSCLC cell lines, A549 and NCI-H1299. Through network pharmacological analyses, including target prediction, Reactome pathway enrichment, Gene Oncology (GO) function analysis, and protein–protein interaction (PPI), together with targets validation using molecular docking, qRT-PCR, and western blotting, the possible mechanism of anticancer effect of diosbulbin C in NSCLC was also preliminarily explored. Our results demonstrated that diosbulbin C induces G0/G1 phase cell cycle arrest and inhibits cell proliferation in NSCLC, possibly by downregulating the expression/activation of AKT, DHFR, and TYMS. Besides, results of ADMET prediction also strongly suggest the high potential of diosbulbin C being developed as an effective anti-lung cancer drug. The research flow chart of this study on diosbulbin C as a potential anti-lung cancer drug is shown in Fig. 1.

## Materials and methods

### Cell culture

The human NSCLC cell lines A549 and NCI-H1299 (H1299) and Human Embryonic Lung Fibroblast Cells (HELF) were purchased from the National Collection of Authenticated Cell Cultures. A549 and H1299 cells were cultured in RPMI-1640 medium (VISTECH), which contained 10% FBS (VISTECH) and 1% Penicillin–Streptomycin (BasalMedia). All cells were maintained in a 5% CO_2_ incubator at 37 °C. The diosbulbin C was purchased from Shanghai yuanye Bio-Technology Co., Ltd (B51007). The cytotoxicity of diosbulbin C dissolved in DMSO in NSCLC cells was determined using Cell Counting Kit-8 (CCK-8, Meilunbio).

### Colony-formation assay

A549 and H1299 cells are plated in 10 cm dishes at a density of 5 × 10^3^ per well with 2 mL cell culture medium. Cells were separately treated with 100 and 200 μM diosbulbin C for 48 h, followed by 10 days’ culture in a complete growth medium. Colony formation is observed under the microscope. After fixing with 4% fixative solution and staining with 0.1% crystal violet solution, the colonies were imaged and counted.

### EdU assay

BeyoClick™ EdU (5-Ethynyl-2’-deoxyuridine) Cell Proliferation Kit with Alexa Fluor 488 (C0071S) was purchased from Beyotime and used to investigate cell proliferation ability. Cells were seeded into 6-well plates and incubated for 24 h, followed by diosbulbin C treatment at different concentrations for 48 h. The EdU assay was conducted according to the kit’s instructions. Briefly, pre-warmed EDU at a dilution of 1:1000 was added to cell cultures, followed by two hours of incubation. Cells were then fixed and permeabilized with 4% formaldehyde and PBS with 0.3% Triton X-100, respectively. Cell nuclei were stained with Hoechst, and the results were visualized by an inverted fluorescent microscope.

### Apoptosis and cell cycle analysis

Cell apoptosis and cell cycle were measured using Annexin V–FITC/PI Apoptosis Detection Kit (YEASEN) and Cell Cycle Staining Kit (MULTI SCIENCES), respectively. Cells were seeded into 6-well plates with a density of 3 × 10^5^ per well with 2 mL cell culture medium. After treating the cells with different concentrations of diosbulbin C for 48 h, apoptosis and cell cycling were analyzed by flow cytometry (CytoFLEX S, Beckman Coulter). Data obtained from flow cytometry were processed by CytExpert 2.4 software.

### Target exploration of diosbulbin C in NSCLC

SwissTargetPrediction (http://www.swisstargetprediction.ch) is a freely available online database used to predict the potential targets of diosbulbin C [13]. DisGeNET (http://www.disgenet.org) is a shared database of genes associated with the disease and used to predict potential therapeutic targets of NSCLC [14]. The “drug-target” network was constructed by Cytoscape 3.9.1 [15]. The GO analysis and Reactome pathway enrichment analysis was performed to further explore the biological functions of the identified potential targets using the DAVID database (https://david.ncifcrf.gov) [16]. The differential expression of the targets between normal and lung cancer tissues was analyzed by GEPIA (https://gepia.cancer-pku.cn) [17]. To further study the interactions between target genes, protein–protein interaction (PPI) analysis was carried out using the STRING database (https://string-db.org) [18]. PPI data obtained from STRING was further processed by Cytoscape 3.9.1, a bioinformatics software used for data visualization and integration, to obtain the PPI network [15].

### Target validation

#### Molecular docking and molecular dynamics simulation

To assess the binding affinity of diosbulbin C to potential targets, Discovery Studio (DS) 2019 was used for flexible docking diosbulbin C and candidate targets [19]. Drug ligands and protein receptors were downloaded from PubChem and PDB database, respectively [20]. The target protein was pretreated before molecular docking analysis, including removing water molecules, hydrogenation, and predicting the active pocket. CDOCKER Interaction Energy, ChiFlex Energy, and LibDockScore are the main parameters used to evaluate the docking analysis.

Molecular dynamics (MD) simulation has been widely applied to estimate the structural characterization and study the binding stability of protein ligand systems [21]. After docking, the MD simulation was performed using DS 2019 software. The workflow of MD simulation includes four steps: energy minimization, heating, equilibration, and production dynamics simulation [22]. It was then processed as follows: Macromolecules–Clean protein–Prepare protein; Change forcefield–CHARMm36-Apply forcefield; Run simulation-Solvation; Standard dynamics cascade -Equilibration simulation time is 20 ps—Production simulation time is 200 ps. The root mean square deviation (RMSD) was used to validate the protein–ligand complex stability during the simulation.

#### Western blotting analysis

Cells were harvested after being exposed to 0, 100, and 200 μM of diosbulbin C for 48 h and lysed with a lysis buffer. The protein concentrations were determined using BCA Protein Assay Kit (Beyotime, P0012S). Protein sample was subsequently subjected to SDS–polyacrylamide gel electrophoresis, and then the samples were transferred to PVDF membranes. The membranes were blocked with 5% skim milk at room temperature for one hour and incubated with primary and secondary antibodies. Subsequently, the protein bands were developed with enhanced chemiluminescence. The antibodies used were as follows: Antibodies against β-actin (internal control), AKT, CDK4, CDK6, Cyclin D1, Cyclin E2, and p-RB were purchased from Cell Signaling Technology (CST) and antibodies against DHFR, TYMS were purchased from HUABIO.

#### qRT-PCR

Total RNA from cells was prepared using RNAex Pro Reagent (AG, AG21102) and reverse transcribed using the HiScript III RT SuperMix for qRT-PCR (Vazyme, R323). ChamQ Universal SYBR qRT-PCR Master Mix (Vazyme, Q711) was used to analyze the mRNA expression. The primer sequences were as follows [23, 24]:

- Human DHFR:
  - Forward: ACTGCTGAGATACAGGGAAATG
  - Reverse: GCAGCTTCTT ACTGCAAACAC
- Human TYMS:
  - Forward: CAACGCTGACGACAGAAGAA
  - Reverse: GCTCACTGTTCACCACATAGA
- Human GAPDH:
  - Forward: GGTGTGAACCATGAGAAGTATGA
  - Reverse: GAGTCCTTCCACGATACCAAAG

#### Prediction of ADMET properties

The ADMET (absorption, distribution, metabolism, excretion, toxicology) properties of diosbulbin C were analyzed using ADMET Descriptors which are available with Discovery Studio 2019.

#### Statistics

Statistical analysis and plots were performed using GraphPad Prism 7.0 software. Data in our study were expressed as mean ± SD. *p* < 0.05 was regarded as statistically significant.

## Results

### Diosbulbin C inhibits NSCLC cell proliferation

**Fig. 2:**
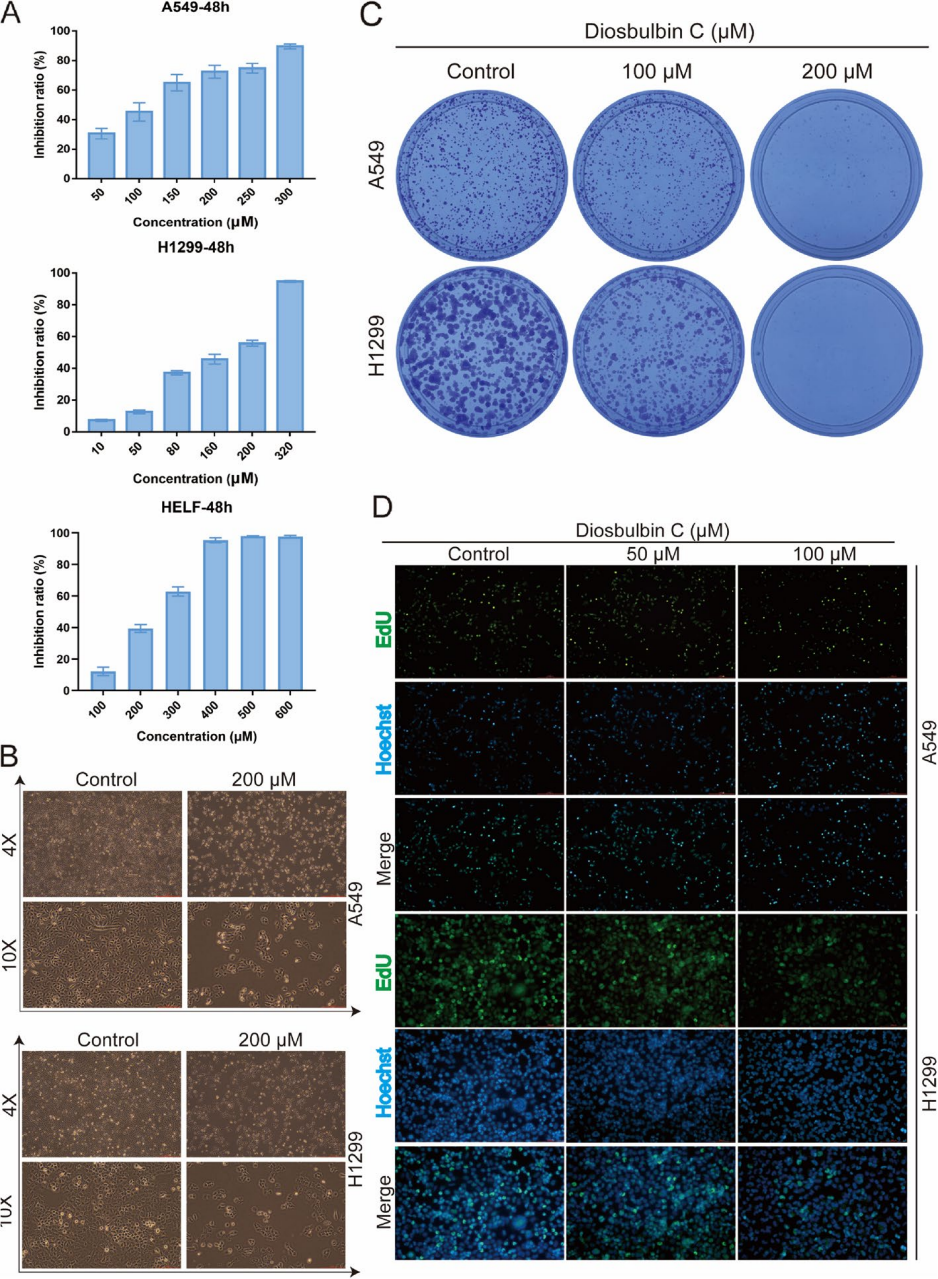
Diosbulbin C inhibits cell viability and proliferation in NSCLC cells. **A** Cell viability was detected using CCK-8 assay after treatment with different concentrations of diosbulbin C in A549, H1299, and HELF cells for 48 h. **B** The cell morphology change was detected under a microscope after the treatment with diosbulbin C for 48 h. **C** Results of the colony formation of A549 and H1299 cells after the treatment with different concentrations of diosbulbin C. **D** Representative results of EdU assay of A549 and H1299 cells with/without diosbulbin C treatment

To explore the anticancer activity of diosbulbin C in NSCLC, a series of cytological experiments was conducted. Firstly, the impact of diosbulbin C on cell viability was investigated using the CCK-8 kit. After exposure to different concentrations of diosbulbin C for 48 h, dose-dependent suppression of diosbulbin C on cell viability was observed in NSCLC cells (Fig. 2A and Supplementary Figure S1). The IC50 (half-maximal inhibitory concentration) values of 100.2 μM, 141.9 μM, and 228.6 μM in A549, H1299, and normal lung HELF cells, respectively, were obtained, suggesting the relatively low cytotoxicity of diosbulbin C to normal cells. Besides, diosbulbin C treatment resulted in significant suppression of NSCLC cell proliferation as observed under the microscope (Fig. 2B). The inhibitory effect of diosbulbin C on the proliferation of NSCLC cells was further evaluated by colony formation and EdU assays. The colony formation results showed that diosbulbin C significantly inhibited the colony formation of NSCLC cells (Fig. 2C). And compared to the control group, the percentage of EdU-positive cells in diosbulbin C treatment groups was also significantly decreased (Fig. 2D). These findings demonstrate the anti-proliferative effect of diosbulbin C on NSCLC cells.

**Fig. 3:**
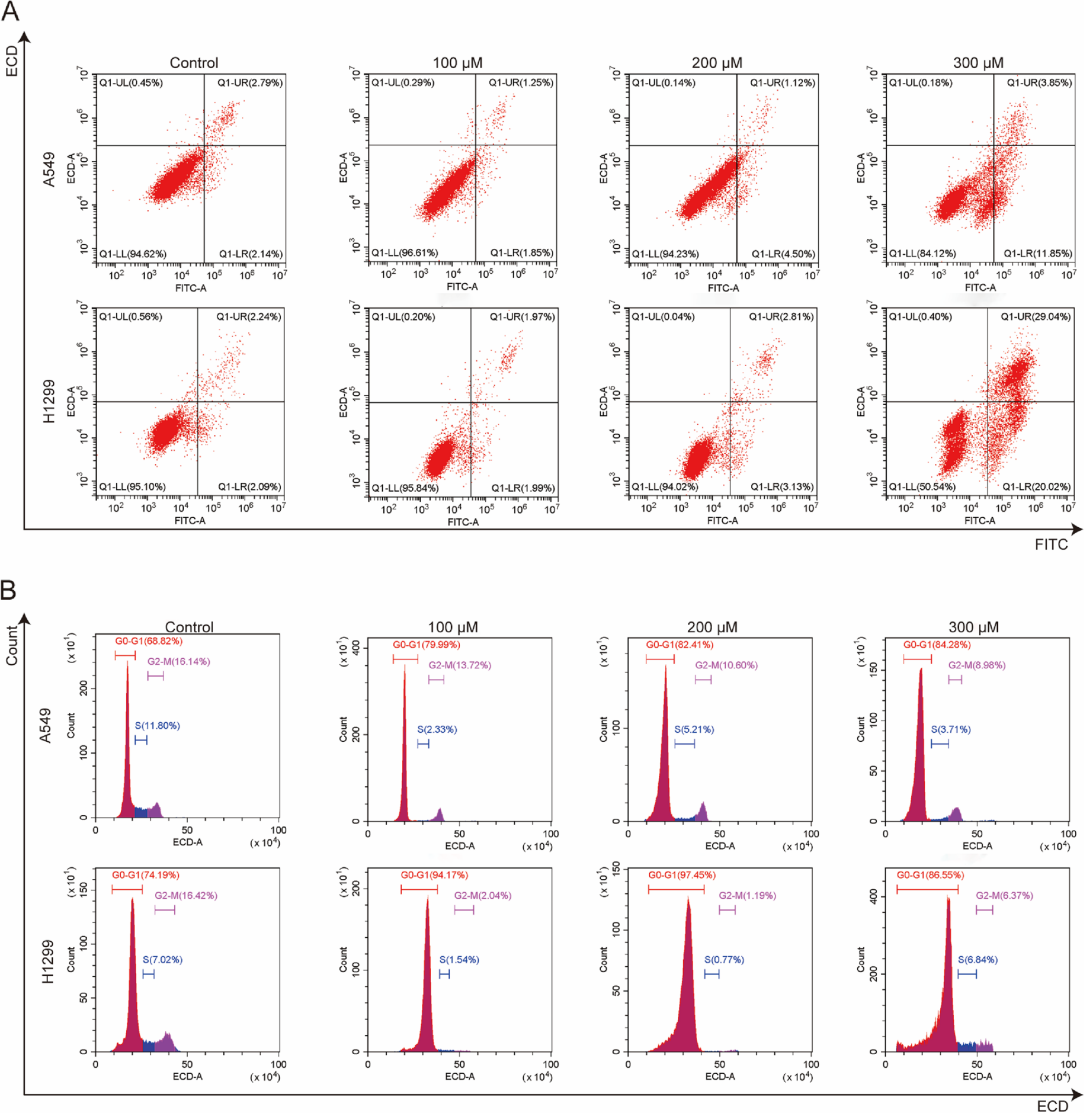
**A** Representative results of annexin V-FITC/PI staining after the treatment with diosbulbin C for 48 h. **B** Representative results of cell cycle after the treatment with diosbulbin C for 48 h

To determine the effect of diosbulbin C on cell apoptosis, we carried out an Annexin V-FITC dual staining assay with flow cytometry. As a result, we found that diosbulbin C can induce NSCLC cell apoptosis, however, only under relatively high concentrations (Fig. 3A). This result suggests that apoptosis induction may not be the predominant effect of diosbulbin C in NSCLC cells. Given the tight connection of cell cycle progression in cancer cells with cell proliferation, we then explored the impact of diosbulbin C on cell cycle in NSCLC cells. After exposing A549 and H1299 cells to 100 μM, 200 μM, and 300 μM of diosbulbin C for 48 h, the cell cycle was subsequently analyzed by flow cytometry. As a result, a significantly increased proportion of G0/G1 phase cells was observed in cells treated with diosbulbin C compared to the control group (Figs. 3B). The results demonstrated that diosbulbin C treatment potently induces G0/G1 phase cell cycle arrest and suppresses cell proliferation in NSCLC cells. Besides, we observed a relatively low proportion of G0/G1 phase cells in 300 μM of diosbulbin C treated group compared to 200 μM treatment, possibly due to the influence of significant cell death induced by high concentration of diosbulbin C (300 μM) in H1299 cells.

### AKT1, DHFR, and TYMS were identified as potential targets of diosbulbin C in NSCLC

**Fig. 4:**
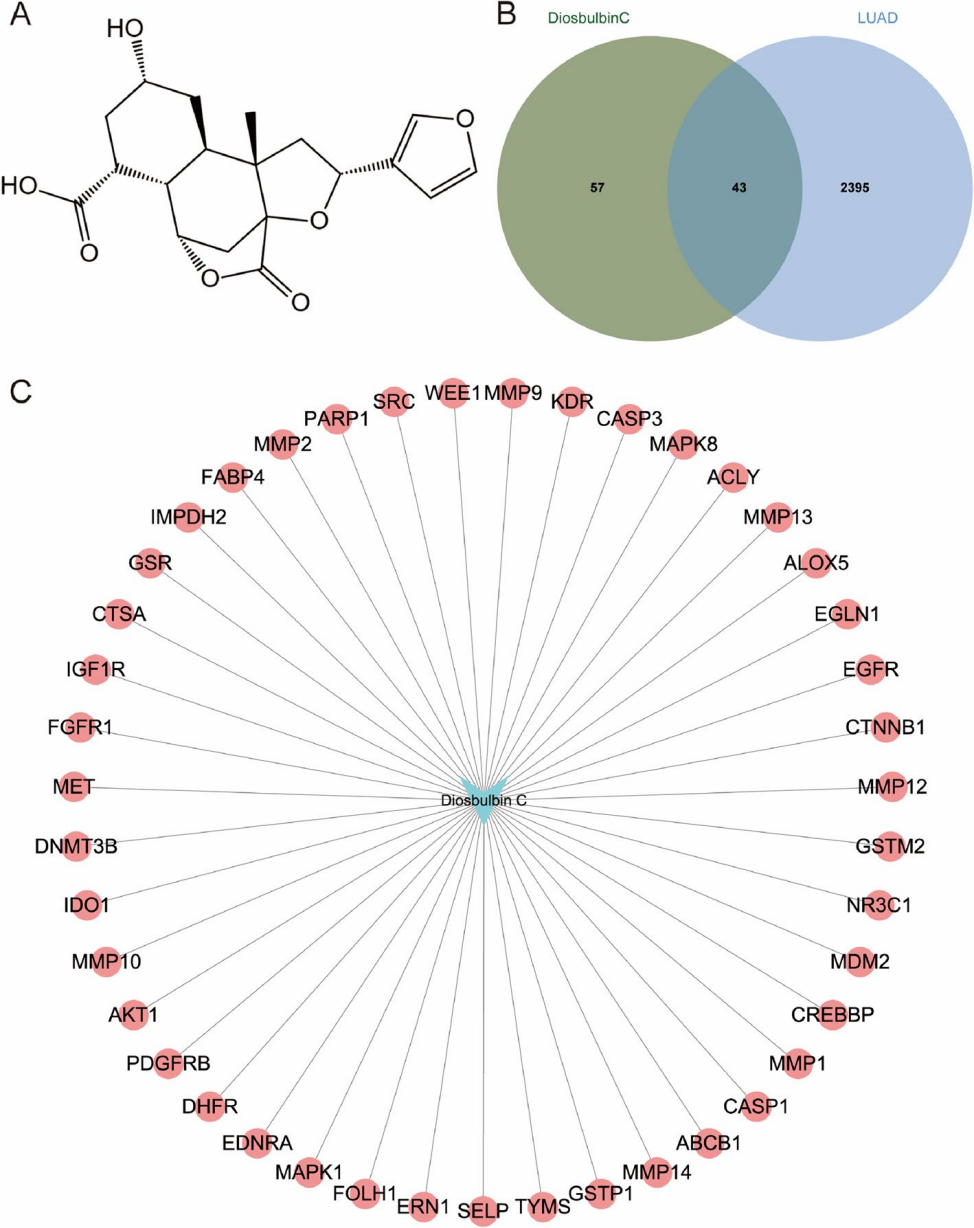
Potential target prediction of diosbulbin C in NSCLC. **A** The molecular structure of diosbulbin C. **B** The collective targets of diosbulbin C and lung cancer were obtained by Venn diagrams. **C** “Drug-target” network was constructed by cytoscape

Based on the molecular formula of the diosbulbin C (Figs. 4A), its 100 potential targets were obtained from the Swiss Target Prediction (Supplementary Table S1). DisGeNET database was then used to identify the potential therapeutic targets of diseases, which resulted in 2438 target genes in NSCLC (Supplementary Table S2). The collective targets of diosbulbin C and NSCLC were obtained by merging the target data using the jvenn online tool, generating 43 overlapping target genes (Fig. 4B). The obtained 43 collective targets were considered as the potential effective targets of diosbulbin C in NSCLC. To better show the interactions of diosbulbin C with its potential targets in NSCLC, we subsequently constructed a “drug-target” network. The interactions between diosbulbin C and the targets are shown in Fig. 4C, which presents 44 nodes and 43 edges in this network, suggesting the multiple interactions of diosbulbin C with the potential targets.

**Fig. 5:**
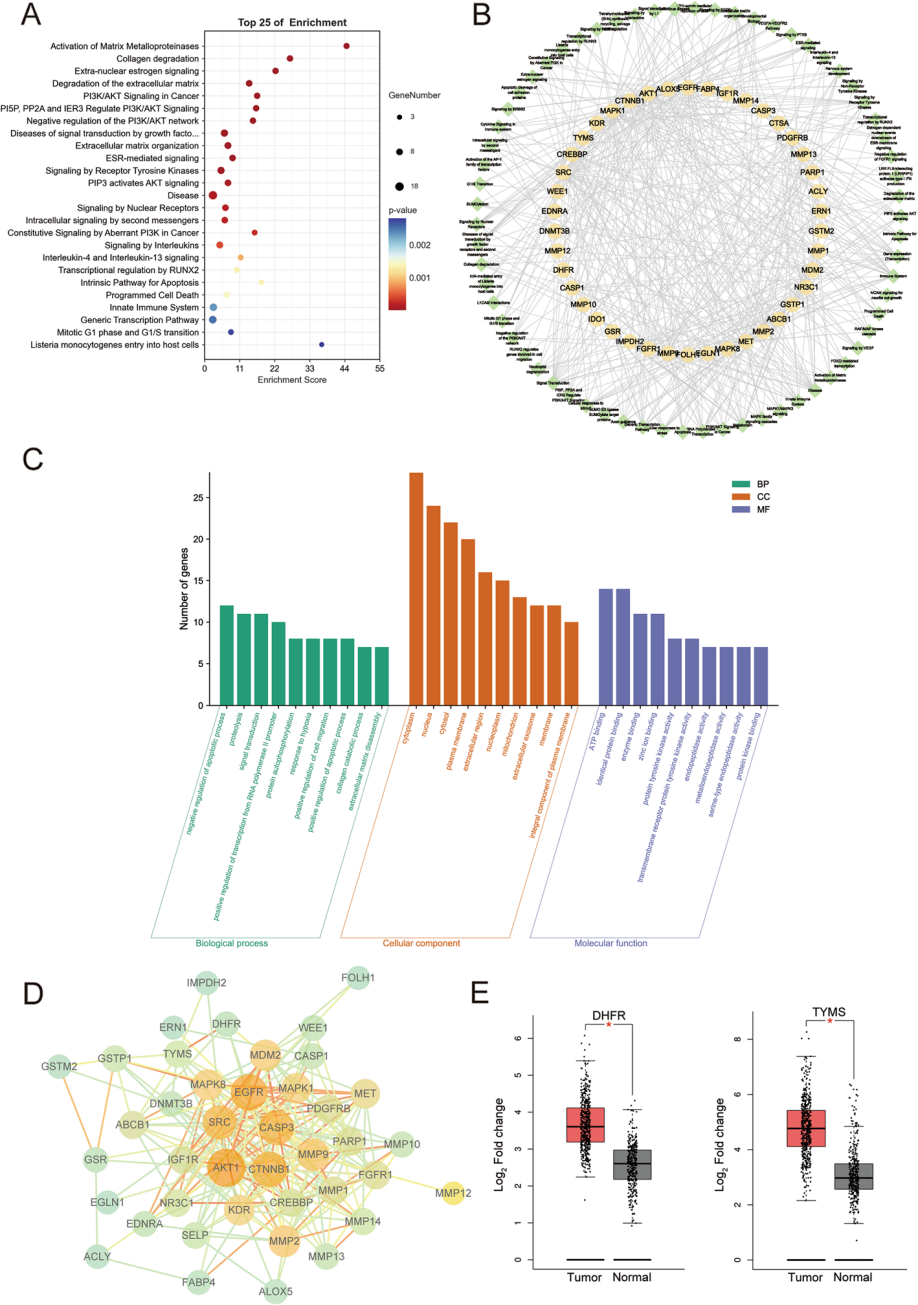
Results of Reactome pathway enrichment, GO analysis, protein–protein interaction, and target protein expressions. **A** The selected top 25 pathways obtained from Reactome pathway enrichment using DAVID are shown. **B** Network of drug “targets-pathways” was constructed. The orange square represents a target, and the green diamond represents a pathway. **C** The results of GO enrichment analysis of the potential targets are shown. **D** The protein–protein interaction network of the potential targets was generated using STRING. **E** The differential expressions of DHFR and TYMS in normal and NSCLC tissues obtained from GEPIA database are shown

To further explore the biological functions of the 43 target genes regulated by diosbulbin C in NSCLC and gain deep insights into the roles of diosbulbin C in biological processes against NSCLC, the Reactome enrichment and GO analysis of the obtained 43 target genes were performed using DAVID database. The results of Reactome enrichment analysis showed that the target genes were significantly enriched in 66 Reactome pathways. The top 25 remarkable enriched pathways are shown in Fig. 5A and Table 1. They were highly related to activation of matrix metalloproteinases, collagen degradation, extra-nuclear estrogen signaling, degradation of the extracellular matrix, immune system regulation, and PI3K/AKT signaling in cancer. In particular, *AKT1*, *DHFR*, and *TYMS* were mapped to the pathway of the mitotic G1 phase and G1/S transition and are closely related to cell cycle progression. To discover the core mechanism of the anti-NSCLC effect of diosbulbin C, all pathways and targets were subsequently subject to constructing a “target-pathway” network. As shown in Fig. 5B, the network comprises 108 nodes (42 marks and 66 signaling pathways) and 442 edges, and different node colors indicate the various targets and regulation pathways. Additionally, according to the GO analysis shown in Fig. 5C, the targets of diosbulbin C were mainly enriched in negative regulation of apoptotic process (GO:0043066), signal transduction (GO:0007165), and protein autophosphorylation (GO:0046777), associated with enzyme binding (GO:0019899), and protein tyrosine kinase activity (GO:0004713), etc. The above results suggest that diosbulbin C may exert its anticancer effect by directly targeting multiple targets associated with NSCLC and regulating the pathways involved in the biological processes.

**Table 1:** Top 25 representative pathways obtained from DAVID

Pathway	Count	*P*-value	Genes
Activation of Matrix Metalloproteinases	6	1.72E-07	*MMP14, MMP13, MMP1, MMP2, MMP9, MMP10*
Collagen degradation	7	1.75E-07	*MMP12, MMP14, MMP13, MMP1, MMP2, MMP9, MMP10*
Extra-nuclear estrogen signaling	7	5.32E-07	*SRC, MMP2, MAPK1, AKT1, MMP9, EGFR, IGF1R*
Degradation of the extracellular matrix	8	1.15E-06	*MMP12, MMP14, MMP13, MMP1, CASP3, MMP2, MMP9, MMP10*
PI3K/AKT Signaling in Cancer	7	3.14E-06	*PDGFRB, SRC, MDM2, AKT1, MET, EGFR, FGFR1*
PI5P, PP2A and IER3 Regulate PI3K/AKT Signaling	7	3.51E-06	*PDGFRB, SRC, MAPK1, AKT1, MET, EGFR, FGFR1*
Negative regulation of the PI3K/AKT network	7	5.10E-06	*PDGFRB, SRC, MAPK1, AKT1, MET, EGFR, FGFR1*
Diseases of signal transduction by growth factor receptors and second messengers	11	5.58E-06	*PDGFRB, CREBBP, SRC, MDM2, KDR, MAPK1, CTNNB1, AKT1, MET, EGFR, FGFR1*
Extracellular matrix organization	9	2.04E-05	*MMP12, MMP14, MMP13, MMP1, CASP3, MMP2, KDR, MMP9, MMP10*
ESR-mediated signaling	8	2.48E-05	*CREBBP, SRC, MMP2, MAPK1, AKT1, MMP9, EGFR, IGF1R*
Signaling by Receptor Tyrosine Kinases	11	2.81E-05	*PDGFRB, SRC, KDR, MAPK1, CTNNB1, AKT1, MET, MMP9, EGFR, FGFR1, IGF1R*
PIP3 activates AKT signaling	8	7.82E-05	*PDGFRB, SRC, MDM2, MAPK1, AKT1, MET, EGFR, FGFR1*
Disease	18	1.16E-04	*CTSA, PDGFRB, CREBBP, PARP1, SRC, NR3C1, EGFR, MAPK8, IMPDH2, MDM2, CASP1, KDR, DNMT3B, AKT1, MAPK1, CTNNB1, MET, FGFR1*
Signaling by Nuclear Receptors	8	1.59E-04	*CREBBP, SRC, MMP2, MAPK1, AKT1, MMP9, EGFR, IGF1R*
Intracellular signaling by second messengers	8	1.94E-04	*PDGFRB, SRC, MDM2, MAPK1, AKT1, MET, EGFR, FGFR1*
Constitutive Signaling by Aberrant PI3K in Cancer	5	2.51E-04	*PDGFRB, SRC, MET, EGFR, FGFR1*
Signaling by Interleukins	9	4.07E-04	*MAPK8, MMP1, ALOX5, CASP3, MMP2, CASP1, MAPK1, AKT1, MMP9*
Interleukin-4 and Interleukin-13 signaling	5	8.65E-04	*MMP1, ALOX5, MMP2, AKT1, MMP9*
Transcriptional regulation by RUNX2	5	1.32E-03	*MMP13, SRC, MAPK1, AKT1, NR3C1*
Intrinsic Pathway for Apoptosis	4	1.34E-03	*MAPK8, CASP3, MAPK1, AKT1*
Programmed Cell Death	6	1.40E-03	*MAPK8, CASP3, CASP1, MAPK1, CTNNB1, AKT1*
Innate Immune System	12	2.33E-03	*CTSA, ACLY, CREBBP, MAPK8, SRC, ALOX5, GSTP1, IMPDH2, CASP1, MAPK1, CTNNB1, MMP9*
Generic Transcription Pathway	13	2.52E-03	*CREBBP, PARP1, SRC, GSR, NR3C1, EGFR, MMP13, MDM2, CASP1, AKT1, MAPK1, CTNNB1, MET*
Mitotic G1 phase and G1/S transition	5	2.76E-03	*DHFR, WEE1, SRC, AKT1, TYMS*
Listeria monocytogenes entry into host cells	3	2.82E-03	*SRC, CTNNB1, MET*

To better visualize and quantify the cellular functions of those targets, the 43 potential targets of diosbulbin C in NSCLC were input into the STRING database to construct their protein–protein interactions (PPI). After processing the data by Cytoscape, 41 nodes and 228 edges were identified in the PPI network (Fig. 5D). Notably, *AKT1*, *DHFR*, and *TYMS* show 33, 5, and 9 edges, respectively. The proteins interacting with the above three proteins had a relatively high score, especially the score of *DHFR* interacting with *TYMS* was 0.999 (Supplementary Table S3), suggesting that *AKT1*, *DHFR*, and *TYMS* may be the key targets of diosbulbin C in NSCLC. Gene expression analysis of the target genes using the GEPIA online tool revealed significant overexpression of DHFR and TYMS in lung cancer tissues in comparison to the normal controls (*p* < 0.05) (Fig. 5E). The expression of *MMP2*, *FGFR1*, *PDGFRB*, *SELP*, *CASP1*, *GSTM2*, *ALOX5*, *KDR*, *FABP4*, *MET*, *MMP1*, *MMP12*, *MMP13*, and *MMP9* between lung cancer and normal tissues were also statistically different as analyzed using the GEPIA online tool (Supplementary Figure S2). They may also serve as active targets of diosbulbin C in NSCLC, which need further evaluation.

### Molecular docking analysis suggests the good binding affinity of diosbulbin C to AKT, DHFR, and TYMS proteins

**Fig. 6:**
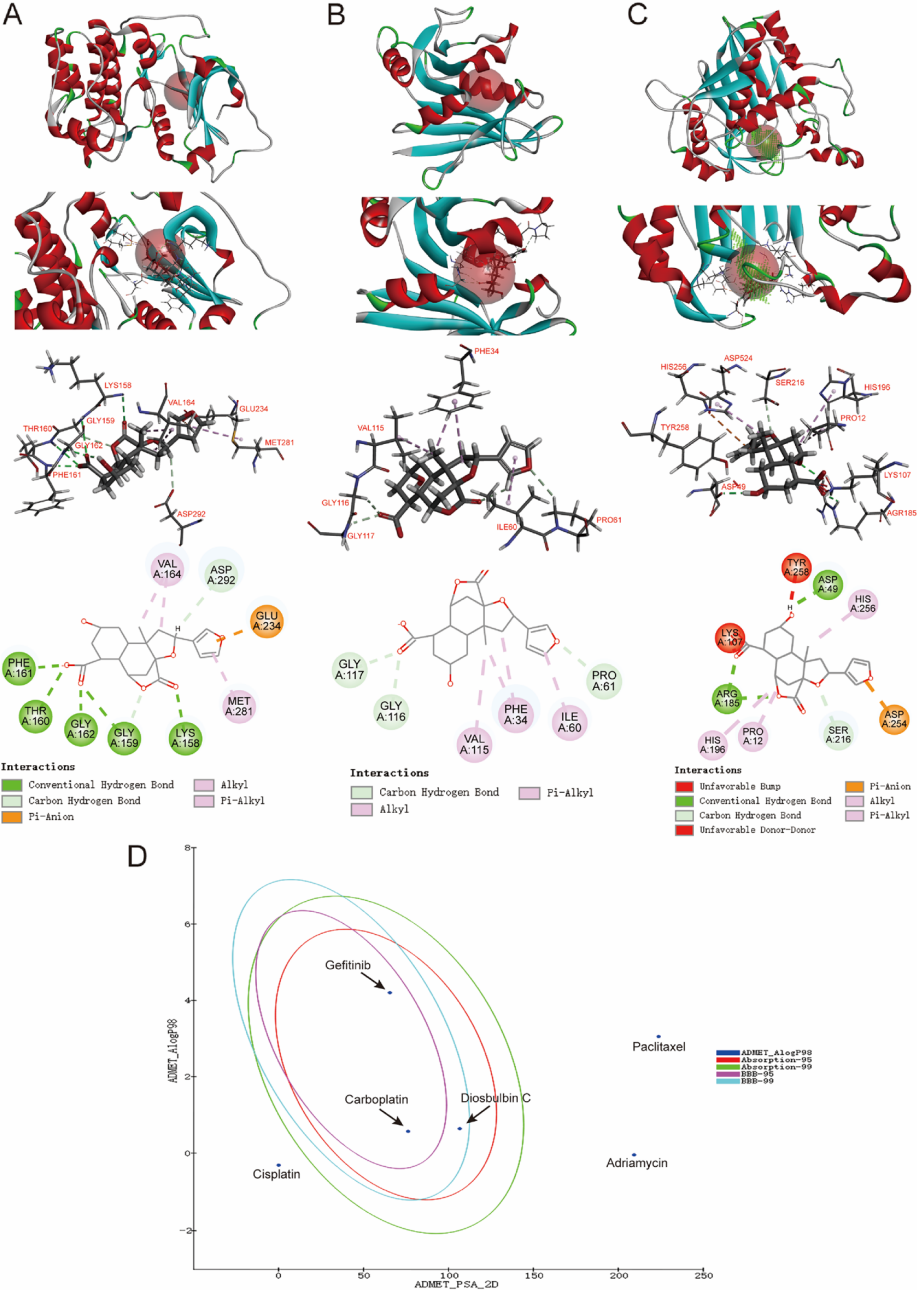
Molecular docking and ADMET of diosbulbin C. **A** Diosbulbin C exhibits high binding affinity to AKT1 protein via sites of Val164, Asp292, Glu234, Met281, Lys158, Gly159, Gly162, Thr160 and Phe161 through molecular docking analysis. **B** Diosbulbin C exhibits high binding affinity to DHFR protein via sites of Gly117, Gly116, Val115, Phe34, Ile60 and Pro61 through molecular docking analysis. **C** Diosbulbin C exhibits high binding affinity to TYMS protein via sites of Tyr258, Asp49, His256, Asp254, Ser216, Pro12, His196, Arg185 and Lys107 through molecular docking analysis. **D** ADMET Plot is plotted by ADMET_PSA_2D vs ADMET_AlogP98. The dark blue dots represent the AlogP98 of diosbulbin C. The red and green ellipses represent 95% and 99% confidence intervals of the human intestinal absorption model, respectively, and the rose red and light blue ellipses represent 95% and 99% confidence intervals of the blood–brain barrier permeability (BBB) model, respectively

Given the above analysis, three proteins (AKT, DHFR, and TYMS) and diosbulbin C were subsequently subjected to molecular docking analysis using Discovery Studio 2019 (DS). The PBD number of the receptor protein is AKT: 4gv1, DHFR: 1kmv, and TYMS: 3gh0, respectively. The result of molecular docking shows high binding affinities of diosbulbin C to AKT protein by the sites of Val164, Asp292, Glu234, Met281, Lys158, Gly159, Gly162, Thr160, and Phe161 with the -CDOCKER_Interaction_Energy of 49.1404 kcal/mole (Fig. 6A, Supplementary Table S4). Besides, diosbulbin C also shows a high binding affinity to DHFR and TYMS with the -CDOCKER_Interaction_Energy of 47.0942 and 72.9033 kcal/mole, respectively (Fig. 6B and C, Supplementary Table S4). Collectively, the molecular docking results revealed a good binding activity of diosbulbin C with AKT, DHFR, and TYMS proteins.

In the present study, AKT, DHFR and TYMS, three potential targets of diosbulbin C against NSCLC, were chosen to further analyze the stability of binding to diosbulbin C. The RMSD value represents the positional changes of the protein compared with the initial conformation during the simulation process. The results of MD simulation analysis were shown in Supplementary Figure S3. The systems of AKT, DHFR, TYMS and diosbulbin C were constantly fluctuating, suggesting that the systems were unstable and were changing constantly. However, the fluctuations of AKT and DHFR were relatively small compared to that of TYMS, suggesting the relatively stable binding of diosbulbin C to these targets.

### Diosbulbin C downregulates the expression/activation of AKT, DHFR, and TYMS in NSCLC cells

**Fig. 7:**
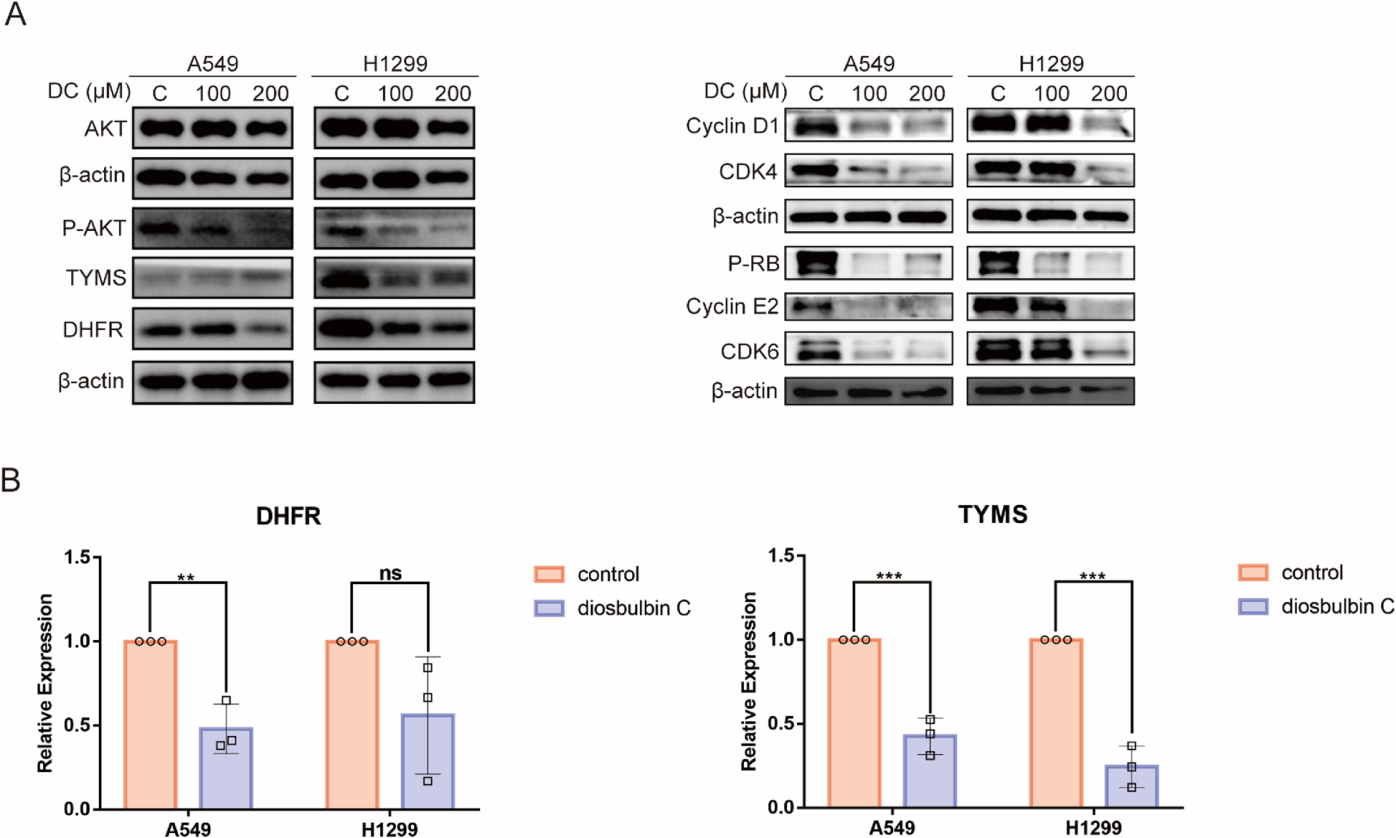
The expression of AKT, DHFR, and TYMS in NSCLC cells after the treatment with/without diosbulbin C. **A** The expressions of AKT, DHFR, TYMS, CDK4, CDK6, Cyclin D1, Cyclin E2, p-RB in A549 and H1299 cells were detected by western blotting after diosbulbin C treatment for 48 h. **B** The expressions of DHFR and TYMS were detected by qRT-PCR after diosbulbin C treatment for 48 h. Data are shown as mean ± SD, *n* = 3. ** *p* < 0.01, ***p < 0.001, ns: no significant difference

To further evaluate the effects of diosbulbin C on potential targets, we carried out the qRT-PCR and western blotting analysis. Considering the G0/G1 phase cell cycle arrest induced by diosbulbin C, the expression of AKT, p-AKT, TYMS, DHFR, CDK4, CDK6, Cyclin D1, Cyclin E2, and p-RB proteins, which were highly correlated with cell cycle progression, was analyzed using western blotting. As shown in Fig. 7A, the expression of p-AKT, TYMS, DHFR, CDK4, CDK6, Cyclin D1, Cyclin E2, and p-RB was significantly downregulated both in A549 and H1299 cells treated with diosbulbin C as compared to the untreated groups, further proving the effect of diosbulbin C on cell cycle progression in NSCLC. Besides, the expression of DHFR and TYMS was also analyzed using qRT-PCR, which shows significantly decreased expression in cells treated with diosbulbin C compared with the untreated group (Fig. 7B). These results strongly suggest that diosbulbin C may induce G0/G1 phase cell cycle arrest and inhibit NSCLC cell proliferation by downregulating the expression/activation of AKT, DHFR, and TYMS in NSCLC cells.

### Diosbulbin C exhibits satisfactory predicted ADMET properties

The prediction of ADMET properties of a drug is an essential method in contemporary drug design and drug screening. ADMET Descriptors and Filter by Lipinski tools which are available with DS, are employed to predict the ADMET properties of diosbulbin C and assess its drug-likeness. Property parameters, such as aqueous solubility, human intestinal absorption, blood–brain barrier (BBB) penetration, and hepatotoxicity, were generated from the analysis. According to the Lipinski Rule of Five (ROF), which is used to estimate drug-likeness, diosbulbin C exhibits satisfactory ADMET properties (Table 2 and Supplementary Figure S4). Firstly, as demonstrated in Fig. 6D, diosbulbin C is within the 99% confidence interval and is considered reliable for predictions. Diosbulbin C has a molecular weight of 362.374, which is suitable for being developed as a drug. Besides, the ALogP of diosbulbin C is predicted to be 0.639, suggesting a good solubility of diosbulbin C. The numbers of H-bond receptors and donors (six H-bond receptors and two H-bond donors in diosbulbin C) are also within the acceptable range, according to the ROF. Moreover, the levels of solubility, intestinal absorption, and BBB penetration are 3, 0, and 3, respectively, which indicates good aqueous solubility and intestinal absorption, however, low BBB penetration of diosbulbin C (Tables 3 and 4). Cytochrome P450s is a vital enzyme system for drug metabolism in liver. According to the analysis, diosbulbin C is not able to inhibit cytochrome P450 2D6, like most of the chemotherapeutic drugs in lung cancer treatments, such as cisplatin (Table 5). Notably, the result shown in Table 6 suggests no hepatotoxicity would be induced by diosbulbin C. Besides, plasma protein binding (PPB) of a drug can not only determine the diffusion or transport of the drug but also influence its action and efficacy. Our result shows a low plasma protein binding rate of diosbulbin C (Table 7). Moreover, diosbulbin C is predicted to be no mutagenicity and has a rat oral LD50 of 1.11 g/kg, further suggesting its suitability for drug development.

**Table 2:** The Lipinski Rule of Five of diosbulbin C obtained from the Lipinski tools in DS

Molecular_Weight	362.374
ALogP	0.639
Num_Rotatablebonds	2
Mocular_PolarSurfaceArea	106.2
Num_H_Acceptors	6
Num_H_Donnors	2

**Table 3:** Blood brain barrier penetration and human intestinal absorption prediction of diosbulbin C and reference drugs

Compound	BBB	BBB Level	Absorption Level	ALogP98	PSA_2D
Diosbulbin C	-1.644	3 (low)	0 (good absorption)	0.640	106.647
Cisplatin	-	4 (undefined)	2 (low absorption)	-0.311	0
Carboplatin	-1.184	3 (low)	0 (good absorption)	0.569	76.232
Gefitinib	0.109	1 (high)	0 (good absorption)	4.203	65.474
Paclitaxel	-	4 (undefined)	3 (very low absorption)	3.055	223.712
Adriamycin	-	4 (undefined)	3 (very low absorption)	-0.044	209.310

**Table 4:** Aqueous solubility prediction of diosbulbin C and reference drugs

Compound	Solubility	Solubility Level
Diosbulbin C	-2.779	3 (yes, good)
Cisplatin	0.776	5 (no, too soluble)
Carboplatin	-0.553	4 (yes, optimal)
Gefitinib	-5.561	2 (yes, low)
Paclitaxel	-3.515	3 (yes, good)
Adriamycin	-4.798	2 (yes, low)

**Table 5:** Cytochrome P450 2D6 inhibitor prediction of diosbulbin C and reference drugs

Compound	CYP2D6	Prediction	Applicability #MD	Applicability #Mdpvalue
Diosbulbin C	-8.41546	FALSE	13.0778	2.53E-04
Cisplatin	-3.65351	FALSE	24.1542	9.31E-17
Carboplatin	-4.16077	FALSE	16.8463	1.55E-08
Gefitinib	2.41414	TRUE	24.2807	6.84E-17
Paclitaxel	-9.84616	FALSE	12.6519	6.87E-04
Adriamycin	-8.00888	FALSE	18.2551	3.60E-10

**Table 6:** Hepatotoxicity prediction of diosbulbin C and reference drugs

Compound	Hepatotoxic	Prediction	Applicability #MD	Applicability #Mdpvalue
Diosbulbin C	-5.52164	FALSE	10.3491	0.0398
Cisplatin	0.891281	TRUE	9.01983	0.4470
Carboplatin	-4.24153	FALSE	7.78939	0.9326
Gefitinib	-3.99306	TRUE	14.2139	2.99E-09
Paclitaxel	-9.53069	FALSE	16.426	3.21E-15
Adriamycin	1.42634	TRUE	14.8384	7.86E-11

**Table 7:** Plasma protein binding (PPB) rate prediction of diosbulbin C and reference drugs

Compound	PPB	Prediction	Applicability #MD	Applicability #Mdpvalue
Diosbulbin C	-5.85853	FALSE	14.0422	8.61E-05
Cisplatin	-2.4272	FALSE	12.0232	0.0899
Carboplatin	-6.36358	FALSE	12.7016	0.0148
Gefitinib	2.80835	TRUE	18.2978	7.72E-17
Paclitaxel	19.7216	TRUE	15.1283	3.26E-07
Adriamycin	-44.2484	FALSE	12.3558	0.0397

## Discussion

Lung cancer is one of the leading causes of cancer deaths worldwide. NSCLC is the most common type of lung cancer. NSCLC patients are commonly diagnosed at an advanced stage and show a low postoperative survival rate. Surgical resection is the most effective method for treating non-small cell lung cancer, but the recurrence rate of tumors is high [25]. In addition to surgical excision, platinum-based chemotherapy, such as cisplatin (DDP) and carboplatin, is one of the first-line non-targeted drugs for treating human NSCLC [25]. However, the clinical outcomes of such drugs are frequently found to be limited due to the development of drug resistance as well as their severe adverse side effects [26]. Progress of targeted therapies and immunotherapies has certainly contributed to improving the survival of patients with NSCLC. However, only limited patients will benefit from these therapies. The requirement of developing novel therapeutic interventions against NSCLC is still urgent.

Traditional Chinese medicine, especially traditional Chinese herbal medicine (CHM), has been continually serving as a supplier of bioactive molecules in drug discovery. China is a major CHM country and has been using CHM for centuries in preventing and treating diseases [27]. In recent decades, the application of CHM is also increasingly popular worldwide. In the field of cancer therapy, CHM has been proved to be an excellent adjuvant therapy, particularly with functions of reducing side effects and complications during chemotherapy and radiotherapy [28–30]. Importantly, a growing body of research has demonstrated that quite a lot of CHMs exhibit excellent anti-cancer activities, and so as their bioactive components. For example, erianin, which is extracted from CHM *Dendrobium chrysotoxum* Lindl. was found to exert anticancer activities in various cancers via regulating multiple bioprocesses [31]. It can exert anti-hepatocellular carcinoma activity via inducing DNA damage and aberrant mitosis [32]. The bioactive compound Theabrownin, extracted from green tea, was demonstrated to be a potential anticancer agent by inducing cell apoptosis and cell cycle arrest in oligodendroglioma and Astrocytoma [33]. Besides, it was reported that ginkgetin extracted from Ginkgo biloba showed a synergistic effect with cisplatin in NSCLC by inducing ferroptosis [34]. Diosbulbin C is a bioactive molecule derived from traditional herbal medicine *Dioscorea bulbifera* L. Although *Dioscorea bulbifera* L. has been historically used in cancer therapy, the anticancer activity of its component diosbulbin C has yet to be investigated.

The cell cycle is mainly divided into G0/G1, S, G2 and M phases, which are controlled by different cyclin-dependent kinases (CDKs) and their functional cell cycle protein chaperones [35]. One of the hallmarks of cancer cells is uncontrolled cell proliferation [36]. CDK4 and CDK6 are key mediators of cellular entry into S-phase by initiating phosphorylation of the retinoblastoma (RB) protein family. Activation of the E2F transcription factors, cyclin E, and CDK2 expression leads to further phosphorylation of RB and ultimately cells entry into S phase [37]. Therefore, effective inhibition of CDK4/CDK6 expression can lead to cell cycle arrest, resulting in tumor growth suppression. It has been shown that paeonol induced colorectal cancer cells to arrest in the G0/G1 phase by inhibiting the Wnt/β-catenin signaling pathway [38], saikosaponin‑d inhibited the proliferation of prostate cancer cells through cell cycle arrest in G0/G1 phase [39]. In this study, we found that diosbulbin C could suppress lung cancer cell proliferation possibly by inducing G0/G1 phase cell cycle arrest.

In this study, we explored the anti-lung cancer activity of diosbulbin C for the first time. From the results of cytology experiments, we found that diosbulbin C can effectively inhibit the proliferation of NSCLC cells, possibly by inducing cell cycle arrest in G0/G1 phase. Besides, diosbulbin C shows relatively low cytotoxicity to normal cells (HELF). Through network pharmacology analysis, 43 potential targets of diosbulbin C in NSCLC were identified. Among them, *AKT1*, *DHFR*, and *TYMS* were highly enriched in the pathway of the mitotic G1 phase and G1/S transition in the cell cycle. Being a central node of many signaling pathways, the activated AKT regulates the functions of numerous downstream proteins that regulate cell survival, cell cycle progression, and cell growth [40]. It has long been identified as an oncogene in cancers [41], and targeting AKT could be a promising strategy in precision cancer therapy [42]. Several AKT inhibitors in clinical trials show promise in treating solid tumors, such as ipatasertib and capivasertib, particularly when combined with other chemotherapeutic drugs (clinicaltrials.gov, NTC02162719, NCT04341259, NCT04467801, NCT02162719, NCT01896531, NCT04742036, NCT05348577). Exploring natural AKT inhibitors from CHM can be valuable for cancer chemoprevention and therapy. Herein, we found that diosbulbin C can significantly inhibit the expression of AKT in NSCLC cells. The downregulated expression of AKT may thus contribute to the induction of cell cycle arrest and the suppressive effect of diosbulbin C on NSCLC cell proliferation. Besides, DHFR and TYMS are the other two potential targets that were downregulated by diosbulbin C in NSCLC cells. Zhao et al. have demonstrated that DHFR and TYMS are the canonical folate pathway genes that promote the proliferation of glioma cells, whereas loss of DHFR/TYMS increases apoptotic glioma cells [23]. Thus, suppression of DHFR and TYMS expression in cells may also confer the inhibition of cell growth and induction of cell death in NSCLC by diosbulbin C.

In addition, by performing the in silico ADMET properties prediction, we found that Diosbulbin C exhibits satisfactory ADMET properties with good water solubility and intestinal absorption and without hepatotoxicity. However, it should be noted that diosbulbin C is potentially carcinogenic, like most chemotherapeutic drugs [43]. Still, the presented study revealed the high potential of diosbulbin C in anti-lung cancer drug discovery. It may also serve as a compound skeleton for the development or synthesis of more effective anti-lung cancer drugs in the future.

## Conclusions

In conclusion, our study, for the first time, demonstrated the anticancer activity of diosbulbin C in NSCLC. There were 43 NSCLC-related targets of diosbulbin C being identified through network pharmacology, with three further being evaluated by molecular docking and qRT-PCR or western blotting due to their relatively more robust interactions in the PPI network and essential roles in cell cycle progression. Our results suggest that diosbulbin C induces cell cycle arrest and inhibits the proliferation of NSCLC cells, possibly by downregulating the expression/activation of AKT, DHFR, and TYMS.

### Supplementary Information

**Additional file 1: Figure S1.** The inhibitory effects of diosbulbin C on A549 and H1299 cells after 24 h and 48 h treatments. **Figure S2.** The differential expressions of CASP1, MET, GSTM2, ALOX5, KDR, FGFR1, SELP, FABP4, MMP1, MMP2, MMP9, MMP12, and MMP13 in normal and NSCLC tissue obtained from GEPIA database are shown. **Figure S3.** The MD simulation results of AKT, DHFR and TYMS with diosbulbin C. **Figure S4.** The diosbulbin C toxicity report demonstrated that it is predicted to be no mutagenicity. **Table S1.** One hundred predicted targets of diosbulbin C. **Table S2.** Two thousand four hundred thirty-eight potential therapeutic targets of human lung adenocarcinoma. Table S3. String interactions short of PPI. **Table S4.** The results of Molecular docking.

**Additional file 2. **

## Data Availability

All data generated or analyzed during this study are included in this published article and its supplementary information files.

## Abbreviations

LC: Lung cancer
NSCLC: Non-small cell lung cancer
SCLC: Small cell lung cancer
TCM: Traditional Chinese Medicine
GO: Gene Oncology
PPI: Protein–protein interaction
DS: Discovery Studio
CST: Cell Signaling Technology
ADMET: Absorption, distribution, metabolism, excretion, toxicology
BBB: Blood–brain barrier
ROF: Lipinski Rule of Five
PPB: Plasma protein binding
DDP: Cisplatin
CHM: Chinese herbal medicine
IC50: Half-maximal inhibitory concentration
