# Supplementary material for: Diosbulbin C, a novel active ingredient in Dioscorea bulbifera L. extract, inhibits lung cancer cell proliferation by inducing G0/G1 phase cell cycle arrest
Source: BMC Complement Med Ther. 2023 Dec 4;23:436. doi: 10.1186/s12906-023-04245-9 (PMC10694954; doi:10.1186/s12906-023-04245-9)
Supplement: Supplementary file 1 — Additional file 1: Figure S1. The inhibitory effects of diosbulbin C on A549 and H1299 cells after 24 h and 48 h treatments. Figure S2. The differential expressions of CASP1, MET, GSTM2, ALOX5, KDR, FGFR1, SELP, FABP4, MMP1, MMP2, MMP9, MMP12, and MMP13 in normal and NSCLC tissue obtained from GEPIA database are shown. Figure S3. The MD simulation results of AKT, DHFR and TYMS with diosbulbin C. Figure S4. The diosbulbin C toxicity report demonstrated that it is predicted to be no mutagenicity. Table S1. One hundred predicted targets of diosbulbin C. Table S2. Two thousand four hundred thirty-eight potential therapeutic targets of human lung adenocarcinoma. Table S3. String interactions short of PPI. Table S4. The results of Molecular docking. [file 12906_2023_4245_MOESM1_ESM.pdf]

Diosbulbin C, a novel active ingredient in *Dioscorea bulbifera* L. extract, inhibits lung cancer cell proliferation by inducing G0/G1 phase cell cycle arrest

Zhiyu Zhu[1], Yanfen Liu[1], Jiangping Zeng[1], Shuyi Ren[1], Lu Wei[1], Fei Wang[1], Xiaoyu Sun[1], Yufei Huang[1], Haiyang Jiang[1], Xinbing Sui[1,2], Weiwei Jin[2*], Lijun Jin[3*], Xueni Sun[1,2*]

[1] School of Pharmacy, Key Laboratory of Elemene Class Anti-Cancer Chinese Medicines; Engineering Laboratory of Development and Application of Traditional Chinese Medicines; Collaborative Innovation Center of Traditional Chinese Medicines of Zhejiang Province, Hangzhou Normal University, Hangzhou, Zhejiang 311121, China

[2] Department of Gastrointestinal & Pancreatic Surgery, Key Laboratory of Gastroenterology of Zhejiang Province, Zhejiang Provincial People's Hospital, People's Hospital of Hangzhou Medical College, Hangzhou, Zhejiang 310014, China

[3] Department of Traditional Chinese Medicine, Hangzhou Shangcheng District People's Hospital, Hangzhou, China

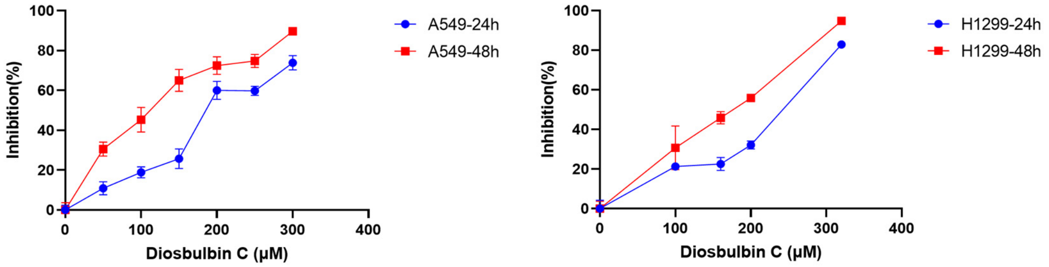

Figure S1. The inhibitory effects of diosbulbin C on A549 and H1299 cells after 24 h and 48 h treatments.

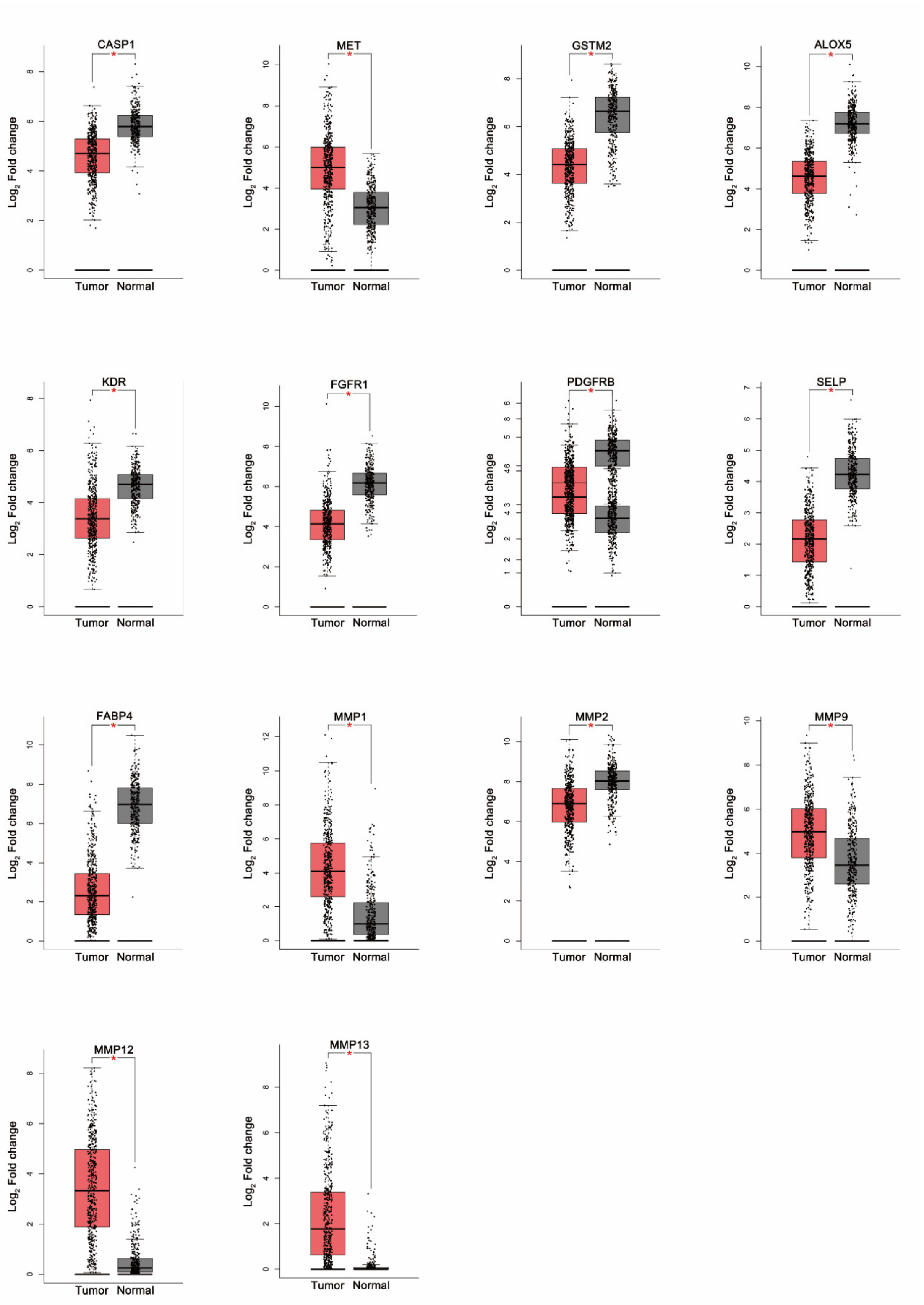

Figure S2. The differential expressions of CASP1, MET, GSTM2, ALOX5, KDR, FGFR1, SELP, FABP4, MMP1, MMP2, MMP9, MMP12, and MMP13 in normal and NSCLC tissue obtained from GEPIA database are shown.

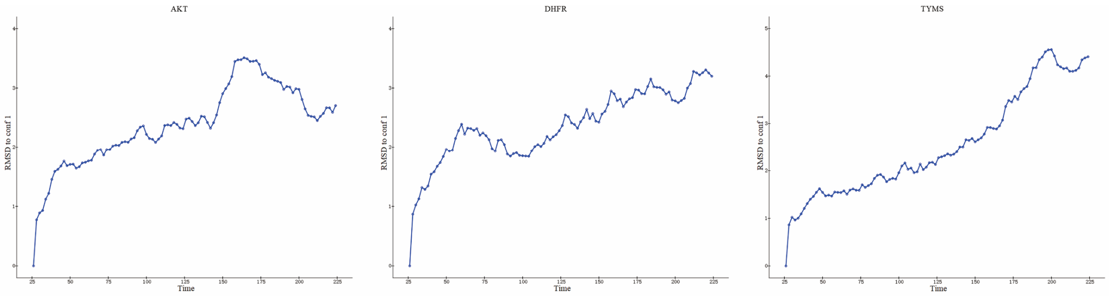

Figure S3. The MD simulation results of AKT, DHFR and TYMS with diosbulbin C.

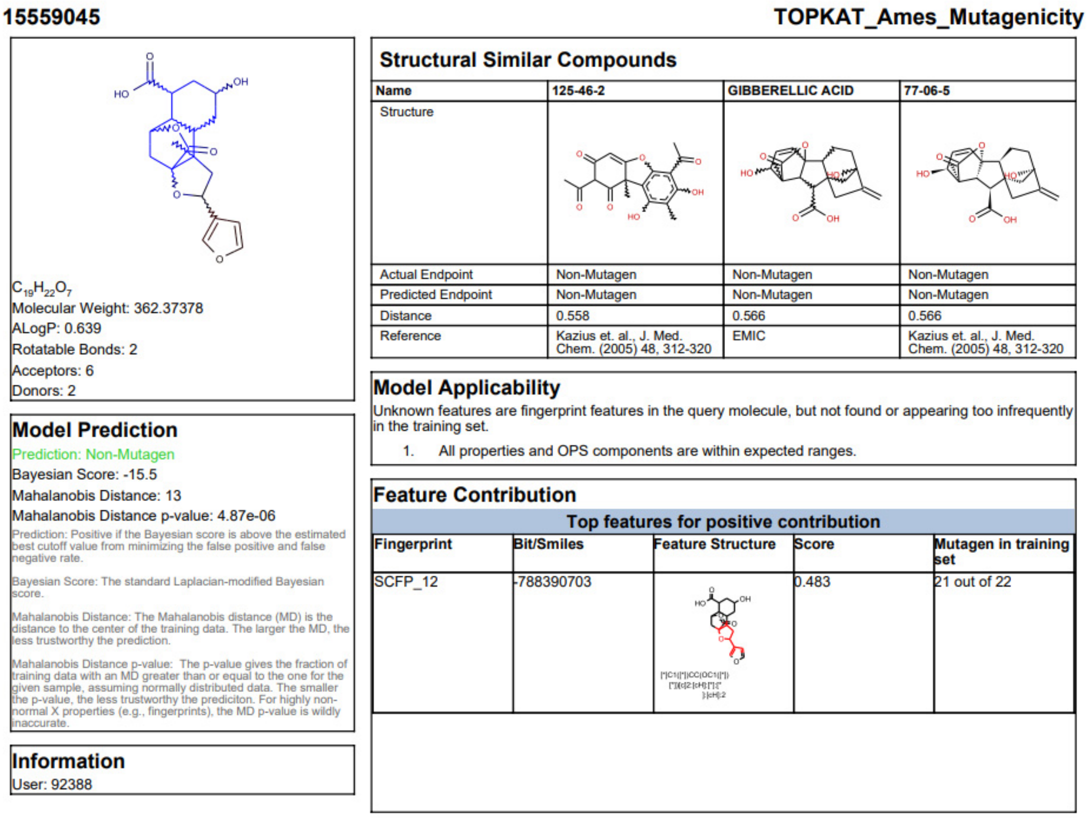

15559045 **TOPKAT_Ames_Mutagenicity**

$C_{19}H_{22}O_7$
Molecular Weight: 362.37378
ALogP: 0.639
Rotatable Bonds: 2
Acceptors: 6
Donors: 2

Structural Similar Compounds

Name	125-46-2	GIBBERELLIC ACID	77-06-5
Structure			
Actual Endpoint	Non-Mutagen	Non-Mutagen	Non-Mutagen
Predicted Endpoint	Non-Mutagen	Non-Mutagen	Non-Mutagen
Distance	0.558	0.566	0.566
Reference	Kazius et. al., J. Med. Chem. (2005) 48, 312-320	EMIC	Kazius et. al., J. Med. Chem. (2005) 48, 312-320

Model Prediction
Prediction: Non-Mutagen
Bayesian Score: -15.5
Mahalanobis Distance: 13
Mahalanobis Distance p-value: 4.87e-06

Prediction: Positive if the Bayesian score is above the estimated best cutoff value from minimizing the false positive and false negative rate.

Bayesian Score: The standard Laplacian-modified Bayesian score.

Mahalanobis Distance: The Mahalanobis distance (MD) is the distance to the center of the training data. The larger the MD, the less trustworthy the prediction.

Mahalanobis Distance p-value: The p-value gives the fraction of training data with an MD greater than or equal to the one for the given sample, assuming normally distributed data. The smaller the p-value, the less trustworthy the prediciton. For highly non-normal X properties (e.g., fingerprints), the MD p-value is wildly inaccurate.

Model Applicability
Unknown features are fingerprint features in the query molecule, but not found or appearing too infrequently in the training set.

1. All properties and OPS components are within expected ranges.

Feature Contribution

Top features for positive contribution				
Fingerprint	Bit/Smiles	Feature Structure	Score	Mutagen in training set
SCFP_12	-788390703		0.483	21 out of 22

Information
User: 92388

Figure S4. The diosbulbin C toxicity report demonstrated that it is predicted to be no mutagenicity.

Table S1. 100 predicted targets of diosbulbin C.

Number	Target	Common name
1	Kappa Opioid receptor	OPRK1
2	Delta opioid receptor	OPRD1
3	Mu opioid receptor	OPRM1
4	Glutathione reductase	GSR
5	AMP deaminase 3	AMPD3
6	AMP deaminase 2	AMPD2
7	Inosine-5'-monophosphate dehydrogenase 2	IMPDH2
8	P-selectin	SELP
9	Free fatty acid receptor 1	FFAR1
10	Leukocyte adhesion molecule-1	SELL
11	Selectin E	SELE
12	Aldose reductase	AKR1B1
13	Leukotriene B4 receptor 1	LTB4R
14	Thromboxane-A synthase	TBXAS1
15	HMG-CoA reductase	HMGCR
16	Neprilysin	MME
17	P-glycoprotein 1	ABCB1
18	Serine/threonine-protein kinase AKT	AKT1
19	Indoleamine 2,3-dioxygenase	IDO1
20	Matrix metalloproteinase 13	MMP13
21	Matrix metalloproteinase 12	MMP12
22	Casein kinase II alpha	CSNK2A1
23	Leukotriene A4 hydrolase	LTA4H
24	Cyclooxygenase-1	PTGS1
25	CREB-binding protein/p53	CREBBP
26	Solute carrier family 22 member 12	SLC22A12
27	LanC-like protein 2	LANCL2
28	Angiotensin-converting enzyme	ACE
29	ATP-citrate synthase	ACLY
30	Serotonin transporter	SLC6A4
31	Caspase-3	CASP3
32	Glucocorticoid receptor	NR3C1
33	Fructose-1,6-bisphosphatase	FBP1
34	Endothelin-converting enzyme 1	ECE1
35	Serine/threonine-protein kinase WEE1	WEE1
36	Integrin alpha-4/beta-1	ITGB1 ITGA4
37	Platelet-derived growth factor receptor beta	PDGFRB
38	GABA-A receptor; alpha-1/beta-2/gamma-2	GABRA1 GABRB2 GABRG2
39	Vascular endothelial growth factor receptor 2	KDR
40	Membrane metallo-endopeptidase-like 1	MMEL1
41	Fibroblast growth factor receptor 1	FGFR1

42	Inosine-5'-monophosphate dehydrogenase 1	IMPDH1
43	p53-binding protein Mdm-2	MDM2
44	Epidermal growth factor receptor erbB1	EGFR
45	c-Jun N-terminal kinase 1	MAPK8
46	Tyrosine-protein kinase SRC	SRC
47	Lysosomal protective protein	CTSA
48	Liver glycogen phosphorylase	PYGL
49	Poly [ADP-ribose] polymerase-1	PARP1
50	Aminopeptidase N	ANPEP
51	AMP deaminase 1	AMPD1
52	G protein-coupled receptor 44	PTGDR2
53	Carboxypeptidase A1	CPA1
54	Glutathione S-transferase Pi	GSTP1
55	Glutathione S-transferase Mu 2	GSTM2
56	Intercellular adhesion molecule (ICAM-1), Integrin alpha-L/beta-2	ITGAL ICAM1 ITGB2
57	Matrix metalloproteinase 2	MMP2
58	Hydroxyacid oxidase 2	HAO2
59	Phosphodiesterase 5A	PDE5A
60	Serotonin 2b (5-HT2b) receptor	HTR2B
61	Interleukin-1 receptor-associated kinase 4	IRAK4
62	MAP kinase ERK2	MAPK1
63	Axin1/beta-catenin	CTNNB1
64	Prostanoid EP4 receptor	PTGER4
65	Insulin-like growth factor I receptor	IGF1R
66	Tyrosine-protein kinase TIE-2	TEK
67	Carbonic anhydrase II	CA2
68	Carbonic anhydrase I	CA1
69	Carbonic anhydrase XIV	CA14
70	Carbonic anhydrase IV	CA4
71	Carbonic anhydrase XIII	CA13
72	Carbonic anhydrase VA	CA5A
73	Thymidylate synthase	TYMS
74	Dihydrofolate reductase	DHFR
75	Fatty acid binding protein adipocyte	FABP4
76	Matrix metalloproteinase 3	MMP3
77	Hepatocyte growth factor receptor	MET
78	Endothelin receptor ET-A	EDNRA
79	MAP kinase signal-integrating kinase 2	MKNK2
80	Prostaglandin I2 synthase	PTGIS
81	Serine/threonine-protein kinase ILK-1	ILK
82	P2X purinoceptor 3	P2RX3
83	dCTP pyrophosphatase 1	DCTPP1
84	Caspase-1	CASP1

85	Matrix metalloproteinase 9	MMP9
86	Matrix metalloproteinase 1	MMP1
87	Matrix metalloproteinase 14	MMP14
88	Matrix metalloproteinase 8	MMP8
89	Lysine-specific demethylase 4A	KDM4A
90	Egl nine homolog 1	EGLN1
91	DNA (cytosine-5)-methyltransferase 3B	DNMT3B
92	Glutamate carboxypeptidase II	FOLH1
93	Arachidonate 5-lipoxygenase	ALOX5
94	Dopamine transporter	SLC6A3
95	Serine/threonine-protein kinase/endoribonuclease IRE1	ERN1
96	Calpain 1	CAPN1
97	Matrix metalloproteinase 10	MMP10
98	Purine nucleoside phosphorylase	PNP
99	Aldo-keto-reductase family 1 member C3	AKR1C3
100	Lysine-specific demethylase 4D	KDM4D

Table S2. 2438 potential therapeutic targets of human lung adenocarcinoma.

Number	Gene_Full_Name	Gene
1	epidermal growth factor receptor	EGFR
2	ALK receptor tyrosine kinase	ALK
3	KRAS proto-oncogene, GTPase	KRAS
4	ROS proto-oncogene 1, receptor tyrosine kinase	ROS1
5	B-Raf proto-oncogene, serine/threonine kinase	BRAF
6	serine/threonine kinase 11	STK11
7	fibroblast growth factor receptor 3	FGFR3
8	mitogen-activated protein kinase kinase 1	MAP2K1
9	catenin beta 1	CTNNB1
10	erb-b2 receptor tyrosine kinase 2	ERBB2
11	HRas proto-oncogene, GTPase	HRAS
12	MYC proto-oncogene, bHLH transcription factor	MYC
13	phosphatidylinositol-4,5-bisphosphate 3-kinase catalytic subunit alpha	PIK3CA
14	tumor protein p63	TP63
15	CLPTM1 like	CLPTM1L
16	fibroblast growth factor receptor 2	FGFR2
17	Raf-1 proto-oncogene, serine/threonine kinase	RAF1
18	NRAS proto-oncogene, GTPase	NRAS
19	BRCA1 associated protein 1	BAP1
20	APC regulator of WNT signaling pathway	APC
21	A-Raf proto-oncogene, serine/threonine kinase	ARAF
22	SOS Ras/Rac guanine nucleotide exchange factor 1	SOS1
23	aryl hydrocarbon receptor	AHR

24	EPH receptor A5	EPHA5
25	ERCC excision repair 1, endonuclease non-catalytic subunit	ERCC1
26	AKT serine/threonine kinase 1	AKT1
27	EMAP like 4	EML4
28	ATM serine/threonine kinase	ATM
29	tumor protein p53	TP53
30	transcription termination factor 1	TTF1
31	thymidylate synthetase	TYMS
32	kelch like ECH associated protein 1	KEAP1
33	mitogen-activated protein kinase 14	MAPK14
34	stratifin	SFN
35	8-oxoguanine DNA glycosylase	OGG1
36	SRY-box transcription factor 2	SOX2
37	kinase insert domain receptor	KDR
38	X-ray repair cross complementing 5	XRCC5
39	enolase 1	ENO1
40	forkhead box O3	FOXO3
41	apurinic/apyrimidinic endodeoxyribonuclease 1	APEX1
42	fibroblast growth factor receptor 4	FGFR4
43	surfactant protein C	SFTPC
44	WNK lysine deficient protein kinase 1	WNK1
45	cyclin dependent kinase inhibitor 2B	CDKN2B
46	CREB binding protein	CREBBP
47	large tumor suppressor kinase 2	LATS2
48	annexin A1	ANXA1
49	heat shock protein family B (small) member 1	HSPB1
50	aquaporin 4	AQP4
51	ribosomal protein SA	RPSA
52	mutL homolog 1	MLH1
53	ribosomal protein L36a	RPL36A
54	serine hydroxymethyltransferase 1	SHMT1
55	superoxide dismutase 2	SOD2
56	tyrosine kinase non receptor 2	TNK2
57	cytochrome P450 family 2 subfamily A member 13	CYP2A13
58	EPH receptor A3	EPHA3
59	glyceraldehyde-3-phosphate dehydrogenase	GAPDH
60	annexin A5	ANXA5
61	lactate dehydrogenase A	LDHA
62	nucleolin	NCL
63	neurotrophic receptor tyrosine kinase 2	NTRK2
64	ATR serine/threonine kinase	ATR
65	RAS p21 protein activator 1	RASA1
66	DNA topoisomerase II alpha	TOP2A
67	XPC complex subunit, DNA damage recognition and repair factor	XPC

68	TNF superfamily member 10	TNFSF10
69	apolipoprotein B mRNA editing enzyme catalytic subunit 3B	APOBEC3B
70	Fas activated serine/threonine kinase	FASTK
71	endoplasmic reticulum protein 29	ERP29
72	E1A binding protein p300	EP300
73	EPH receptor A8	EPHA8
74	endoplasmic reticulum to nucleus signaling 1	ERN1
75	fatty acid synthase	FASN
76	fms related receptor tyrosine kinase 1	FLT1
77	protein kinase D2	PRKD2
78	Rho GTPase activating protein 35	ARHGAP35
79	phosphoserine aminotransferase 1	PSAT1
80	helicase, lymphoid specific	HELLS
81	heterogeneous nuclear ribonucleoprotein A1	HNRNPA1
82	JunB proto-oncogene, AP-1 transcription factor subunit	JUNB
83	potassium inwardly rectifying channel subfamily J member 4	KCNJ4
84	arginase 1	ARG1
85	karyopherin subunit alpha 2	KPNA2
86	lamin A/C	LMNA
87	neurotrophic receptor tyrosine kinase 3	NTRK3
88	discoidin domain receptor tyrosine kinase 2	DDR2
89	golgi membrane protein 1	GOLM1
90	cap methyltransferase 2	CMTR2
91	protein kinase, DNA-activated, catalytic subunit	PRKDC
92	replication factor C subunit 4	RFC4
93	ribonucleotide reductase regulatory subunit M2	RRM2
94	MNX1 antisense RNA 1 (head to head)	MNX1-AS1
95	Kruppel like factor 5	KLF5
96	thymidine kinase 1	TK1
97	triosephosphate isomerase 1	TPI1
98	titin	TTN
99	N-alpha-acetyltransferase 10, NatA catalytic subunit	NAA10
100	unc-51 like autophagy activating kinase 1	ULK1
101	membrane spanning 4-domains A1	MS4A1
102	serum/glucocorticoid regulated kinase 2	SGK2
103	RNA terminal phosphate cyclase like 1	RCL1
104	nucleophosmin/nucleoplasmin 3	NPM3
105	translocase of outer mitochondrial membrane 40	TOMM40
106	ATP synthase peripheral stalk subunit d	ATP5PD
107	NOP56 ribonucleoprotein	NOP56
108	polo like kinase 2	PLK2
109	nudix hydrolase 21	NUDT21
110	TATA-box binding protein associated factor 1 like	TAF1L
111	transient receptor potential cation channel subfamily M member 6	TRPM6

112	casein kinase 1 epsilon	CSNK1E
113	adenosylhomocysteinase	AHCY
114	eukaryotic translation initiation factor 2B subunit alpha	EIF2B1
115	fibrillarin	FBL
116	albumin	ALB
117	acyl-CoA synthetase long chain family member 4	ACSL4
118	aldolase, fructose-bisphosphate A	ALDOA
119	chaperonin containing TCP1 subunit 5	CCT5
120	ribosome biogenesis regulator 1 homolog	RRS1
121	ribosomal protein L13a	RPL13A
122	phosphatidylinositol glycan anchor biosynthesis class N	PIGN
123	arachidonate 5-lipoxygenase	ALOX5
124	arachidonate 12-lipoxygenase, 12R type	ALOX12B
125	X-ray repair cross complementing 6	XRCC6
126	phosphoribosylglycinamide formyltransferase, phosphoribosylglycinamide synthetase, phosphoribosylaminoimidazole synthetase	GART
127	translocase of inner mitochondrial membrane 10	TIMM10
128	ribosomal protein S6 kinase C1	RPS6KC1
129	eukaryotic translation initiation factor 2 alpha kinase 1	EIF2AK1
130	ribosomal protein S6 kinase A6	RPS6KA6
131	serine/threonine kinase 39	STK39
132	G protein subunit gamma 11	GNG11
133	kinase suppressor of ras 2	KSR2
134	basic leucine zipper and W2 domains 2	BZW2
135	RAN guanine nucleotide release factor	RANGRF
136	mutS homolog 6	MSH6
137	guanylate cyclase 2F, retinal	GUCY2F
138	hydroxysteroid 17-beta dehydrogenase 10	HSD17B10
139	hexokinase 1	HK1
140	hexokinase 2	HK2
141	high mobility group box 2	HMGB2
142	apolipoprotein A1	APOA1
143	inosine monophosphate dehydrogenase 2	IMPDH2
144	insulin receptor related receptor	INSRR
145	eukaryotic translation initiation factor 3 subunit E	EIF3E
146	interleukin 1 receptor associated kinase 1	IRAK1
147	interleukin 1 receptor associated kinase 2	IRAK2
148	importin 5	IPO5
149	mitogen-activated protein kinase kinase kinase 15	MAP3K15
150	lamin B1	LMNB1
151	MOS proto-oncogene, serine/threonine kinase	MOS
152	mutS homolog 3	MSH3
153	ribosomal protein L10a	RPL10A
154	NIMA related kinase 1	NEK1

155	nucleophosmin 1	NPM1
156	pterin-4 alpha-carbinolamine dehydratase 1	PCBD1
157	RNA polymerase I and III subunit D	POLR1D
158	FKBP prolyl isomerase 11	FKBP11
159	TAO kinase 3	TAOK3
160	tripartite motif containing 33	TRIM33
161	NOP58 ribonucleoprotein	NOP58
162	GAR1 ribonucleoprotein	GAR1
163	protein phosphatase 3 catalytic subunit alpha	PPP3CA
164	spermatid perinuclear RNA binding protein	STRBP
165	SCY1 like pseudokinase 2	SCYL2
166	eukaryotic translation initiation factor 2 alpha kinase 2	EIF2AK2
167	peter pan homolog	PPAN
168	p21 (RAC1) activated kinase 5	PAK5
169	aldehyde dehydrogenase 18 family member A1	ALDH18A1
170	ribosomal protein L27a	RPL27A
171	mitochondrial ribosomal protein L12	MRPL12
172	ribonucleotide reductase catalytic subunit M1	RRM1
173	SATB homeobox 1	SATB1
174	serine racemase	SRR
175	glucosamine-phosphate N-acetyltransferase 1	GNPNAT1
176	mitochondrial ribosomal protein S5	MRPS5
177	solute carrier family 15 member 2	SLC15A2
178	solute carrier family 19 member 1	SLC19A1
179	SWI/SNF related, matrix associated, actin dependent regulator of chromatin subfamily c member 1	SMARCC1
180	small nuclear ribonucleoprotein polypeptide G	SNRPG
181	spermidine synthase	SRM
182	aurora kinase C	AURKC
183	transferrin	TF
184	uridine-cytidine kinase 2	UCK2
185	vav guanine nucleotide exchange factor 1	VAV1
186	importin 4	IPO4
187	cysteine and glycine rich protein 3	CSRP3
188	acidic nuclear phosphoprotein 32 family member A	ANP32A
189	calcium/calmodulin dependent protein kinase IV	CAMK4
190	capping actin protein, gelsolin like	CAPG
191	H2B clustered histone 4	H2BC4
192	glutamate rich WD repeat containing 1	GRWD1
193	DOT1 like histone lysine methyltransferase	DOT1L
194	myosin XVIIIB	MYO18B
195	protein serine kinase H2	PSKH2
196	solute carrier family 4 member 4	SLC4A4
197	tyrosine kinase non receptor 1	TNK1

198	poly(A) binding protein cytoplasmic 4	PABPC4
199	succinate-CoA ligase GDP-forming subunit beta	SUCLG2
200	eukaryotic translation initiation factor 2B subunit delta	EIF2B4
201	eukaryotic translation initiation factor 2B subunit epsilon	EIF2B5
202	large tumor suppressor kinase 1	LATS1
203	small nucleolar RNA, C/D box 22	SNORD22
204	sideroflexin 1	SFXN1
205	solute carrier family 4 member 7	SLC4A7
206	apoptosis associated tyrosine kinase	AATK
207	STE20 like kinase	SLK
208	translocase of inner mitochondrial membrane 8A	TIMM8A
209	cell adhesion molecule 1	CADM1
210	cadherin 1	CDH1
211	forkhead box P1	FOXP1
212	MRE11 homolog, double strand break repair nuclease	MRE11
213	cyclin dependent kinase inhibitor 2A	CDKN2A
214	gap junction protein beta 2	GJB2
215	glucose-6-phosphate isomerase	GPI
216	insulin like growth factor 2 receptor	IGF2R
217	nuclear factor, erythroid 2 like 2	NFE2L2
218	H1.10 linker histone	H1-10
219	neuregulin 1	NRG1
220	cyclin dependent kinase 4	CDK4
221	cholinergic receptor nicotinic alpha 5 subunit	CHRNA5
222	SMAD family member 4	SMAD4
223	F-box and WD repeat domain containing 7	FBXW7
224	cholinergic receptor nicotinic alpha 3 subunit	CHRNA3
225	BRCA2 DNA repair associated	BRCA2
226	U2 small nuclear RNA auxiliary factor 1	U2AF1
227	CDKN2B antisense RNA 1	CDKN2B-AS1
228	DnaJ heat shock protein family (Hsp40) member B4	DNAJB4
229	bromodomain PHD finger transcription factor	BPTF
230	GNAS complex locus	GNAS
231	isocitrate dehydrogenase (NADP(+)) 1	IDH1
232	butyrophilin like 2	BTNL2
233	protein tyrosine phosphatase non-receptor type 11	PTPN11
234	cholinergic receptor nicotinic beta 4 subunit	CHRNB4
235	hydroxylysine kinase	HYKK
236	DNA methyltransferase 3 alpha	DNMT3A
237	EPH receptor B1	EPHB1
238	discoidin, CUB and LCCL domain containing 1	DCBLD1
239	major histocompatibility complex, class I, A	HLA-A
240	iron responsive element binding protein 2	IREB2
241	interferon regulatory factor 4	IRF4

242	keratin 8	KRT8
243	ras homolog family member A	RHOA
244	surfactant associated 2	SFTA2
245	notch receptor 4	NOTCH4
246	transcription factor 19	TCF19
247	activin A receptor type 1B	ACVR1B
248	SH2B adaptor protein 3	SH2B3
249	cancer susceptibility 11	CASC11
250	LMCD1 antisense RNA 1	LMCD1-AS1
251	EGFR antisense RNA 1	EGFR-AS1
252	MSH5-SAPCD1 readthrough (NMD candidate)	MSH5-SAPCD1
253	actin related protein 2	ACTR2
254	FOXP4 antisense RNA 1	FOXP4-AS1
255	FRY microtubule binding protein	FRY
256	family with sequence similarity 13 member A	FAM13A
257	long intergenic non-protein coding RNA 824	LINC00824
258	TSBP1 and BTNL2 antisense RNA 1	TSBP1-AS1
259	myelin protein zero like 2	MPZL2
260	HLA complex group 9	HCG9
261	long intergenic non-protein coding RNA 1414	LINC01414
262	long intergenic non-protein coding RNA 1828	LINC01828
263	Yes associated protein 1	YAP1
264	C1D nuclear receptor corepressor	C1D
265	CEA cell adhesion molecule 5	CEACAM5
266	long intergenic non-protein coding RNA 2392	LINC02392
267	long intergenic non-protein coding RNA 1891	LINC01891
268	testis expressed basic protein 1	TSBP1
269	mortality factor 4 like 1	MORF4L1
270	RAB31, member RAS oncogene family	RAB31
271	tripartite motif containing 31	TRIM31
272	ADAM metallopeptidase with thrombospondin type 1 motif 7	ADAMTS7
273	bromodomain adjacent to zinc finger domain 1A	BAZ1A
274	Ras association domain family member 1	RASSF1
275	urocortin 3	UCN3
276	collagen type IV alpha 5 chain	COL4A5
277	mucin like 3	MUCL3
278	vesicle transport through interaction with t-SNAREs 1A	VTI1A
279	tektin 5	TEKT5
280	NIMA related kinase 10	NEK10
281	cytochrome P450 family 2 subfamily A member 6	CYP2A6
282	cytochrome P450 family 21 subfamily A member 2	CYP21A2
283	protein phosphatase 1 regulatory subunit 18	PPP1R18
284	epidermal growth factor	EGF

285	myelin protein zero like 3	MPZL3
286	family with sequence similarity 227 member B	FAM227B
287	EPH receptor B2	EPHB2
288	erb-b2 receptor tyrosine kinase 3	ERBB3
289	estrogen receptor 1	ESR1
290	enhancer of zeste 2 polycomb repressive complex 2 subunit	EZH2
291	glutamate rich 6B	ERICH6B
292	fibroblast growth factor 7	FGF7
293	RALY heterogeneous nuclear ribonucleoprotein	RALY
294	ATP binding cassette subfamily F member 1	ABCF1
295	forkhead box M1	FOXM1
296	single-strand-selective monofunctional uracil-DNA glycosylase 1	SMUG1
297	gamma-aminobutyric acid type B receptor subunit 1	GABBR1
298	GA binding protein transcription factor subunit alpha	GABPA
299	galactokinase 2	GALK2
300	adenylate kinase 5	AK5
301	glucosylceramidase beta pseudogene 1	GBAP1
302	Ras association domain family member 3	RASSF3
303	BRISC and BRCA1 A complex member 1	BABAM1
304	CD274 molecule	CD274
305	hepatocyte growth factor	HGF
306	zinc ribbon domain containing 1	ZNRD1
307	hypoxia inducible factor 1 subunit alpha	HIF1A
308	major histocompatibility complex, class I, B	HLA-B
309	major histocompatibility complex, class II, DQ alpha 1	HLA-DQA1
310	major histocompatibility complex, class II, DQ alpha 2	HLA-DQA2
311	insulin like growth factor 1 receptor	IGF1R
312	interleukin 6	IL6
313	mitoguardin 1	MIGA1
314	potassium voltage-gated channel subfamily H member 1	KCNH1
315	metastasis associated lung adenocarcinoma transcript 1	MALAT1
316	kinesin family member 5B	KIF5B
317	ribosomal protein L18 pseudogene 11	RPL18P11
318	long intergenic non-protein coding RNA 243	LINC00243
319	microRNA 145	MIR145
320	microRNA 21	MIR21
321	HLA complex group 18	HCG18
322	HLA complex group 17	HCG17
323	MDM2 proto-oncogene	MDM2
324	MET proto-oncogene, receptor tyrosine kinase	MET
325	matrix metallopeptidase 2	MMP2
326	matrix metallopeptidase 9	MMP9
327	mutS homolog 5	MSH5
328	methylthioadenosine phosphorylase	MTAP

329	cytochrome c oxidase subunit II	COX2
330	metaxin 1	MTX1
331	mucin 1, cell surface associated	MUC1
332	notch receptor 1	NOTCH1
333	dynactin subunit 4	DCTN4
334	programmed cell death 1	PDCD1
335	cyclin dependent kinase 12	CDK12
336	phosphatidylinositol-4,5-bisphosphate 3-kinase catalytic subunit beta	PIK3CB
337	phosphatidylinositol-4,5-bisphosphate 3-kinase catalytic subunit delta	PIK3CD
338	phosphatidylinositol-4,5-bisphosphate 3-kinase catalytic subunit gamma	PIK3CG
339	GIPC PDZ domain containing family member 2	GIPC2
340	protein phosphatase 2 scaffold subunit Aalpha	PPP2R1A
341	renalase, FAD dependent amine oxidase	RNLS
342	apolipoprotein M	APOM
343	mitogen-activated protein kinase 1	MAPK1
344	mitogen-activated protein kinase kinase 7	MAP2K7
345	beta-2-microglobulin	B2M
346	phosphatase and tensin homolog	PTEN
347	prostaglandin-endoperoxide synthase 2	PTGS2
348	Pvt1 oncogene	PVT1
349	RAD51 paralog B	RAD51B
350	cyclin D1	CCND1
351	BCL2 apoptosis regulator	BCL2
352	ret proto-oncogene	RET
353	complement factor B	CFB
354	ataxin 2	ATXN2
355	long intergenic non-protein coding RNA 1829	LINC01829
356	solute carrier family 2 member 1	SLC2A1
357	secreted phosphoprotein 1	SPP1
358	signal transducer and activator of transcription 3	STAT3
359	HNF1 homeobox B	HNF1B
360	telomerase RNA component	TERC
361	telomerase reverse transcriptase	TERT
362	transforming growth factor beta 1	TGFB1
363	NK2 homeobox 1	NKX2-1
364	tumor necrosis factor	TNF
365	tenascin XB	TNXB
366	complement C2	C2
367	vascular endothelial growth factor A	VEGFA
368	ring finger protein 39	RNF39
369	zinc ribbon domain containing 1 antisense, pseudogene	ZNRD1ASP
370	solute carrier family 2 member 10	SLC2A10
371	caspase 3	CASP3
372	USH1 protein network component harmonin binding protein 1	USHBP1

373	tubulin alpha 1c	TUBA1C
374	prominin 1	PROM1
375	serine/threonine kinase 19	STK19
376	LIM domain binding 2	LDB2
377	synaptogyrin 2	SYNGR2
378	Cdk5 and Abl enzyme substrate 1	CABLES1
379	nuclear assembly factor 1 ribonucleoprotein	NAF1
380	napsin A aspartic peptidase	NAPSA
381	CD44 molecule (Indian blood group)	CD44
382	podoplanin	PDPN
383	MT-CO2 pseudogene 12	MTCO2P12
384	cytochrome P450 family 1 subfamily A member 1	CYP1A1
385	microRNA 195	MIR195
386	ATP binding cassette subfamily B member 1	ABCB1
387	zinc finger E-box binding homeobox 1	ZEB1
388	vimentin	VIM
389	caveolin 1	CAV1
390	CRK proto-oncogene, adaptor protein	CRK
391	ELAV like RNA binding protein 2	ELAVL2
392	erythrocyte membrane protein band 4.1 like 3	EPB41L3
393	mechanistic target of rapamycin kinase	MTOR
394	glutathione S-transferase mu 1	GSTM1
395	interferon gamma	IFNG
396	Janus kinase 2	JAK2
397	SMAD family member 3	SMAD3
398	activator of HSP90 ATPase activity 1	AHSA1
399	CEA cell adhesion molecule 3	CEACAM3
400	follistatin like 1	FSTL1
401	NAD(P)H quinone dehydrogenase 1	NQO1
402	endothelial PAS domain protein 1	EPAS1
403	estrogen receptor 2	ESR2
404	fibroblast growth factor receptor 1	FGFR1
405	ring finger protein 19A, RBR E3 ubiquitin protein ligase	RNF19A
406	DNA polymerase delta interacting protein 2	POLDIP2
407	SET domain containing 2, histone lysine methyltransferase	SETD2
408	insulin like growth factor 1	IGF1
409	microRNA 200b	MIR200B
410	microRNA 210	MIR210
411	mucin 4, cell surface associated	MUC4
412	peroxisome proliferator activated receptor gamma	PPARG
413	BMI1 proto-oncogene, polycomb ring finger	BMI1
414	twist family bHLH transcription factor 1	TWIST1
415	Leptin, serum levels of	LEPQTL1
416	aminoacyl tRNA synthetase complex interacting multifunctional protein 2	AIMP2

417	GRB2 related adaptor protein 2	GRAP2
418	COMMD3-BMI1 readthrough	COMMD3-BMI1
419	RNA binding motif protein 5	RBM5
420	caudal type homeobox 2	CDX2
421	CEA cell adhesion molecule 7	CEACAM7
422	checkpoint kinase 1	CHEK1
423	H3 histone pseudogene 10	H3P10
424	poly(ADP-ribose) polymerase 1	PARP1
425	advanced glycosylation end-product specific receptor	AGER
426	E2F transcription factor 1	E2F1
427	epithelial cell transforming 2	ECT2
428	folate receptor alpha	FOLR1
429	GATA binding protein 3	GATA3
430	heat shock protein 90 alpha family class A member 1	HSP90AA1
431	MET transcriptional regulator MACC1	MACC1
432	C-X-C motif chemokine ligand 8	CXCL8
433	keratin 7	KRT7
434	galectin 1	LGALS1
435	microRNA 125a	MIR125A
436	microRNA 183	MIR183
437	microRNA 30a	MIR30A
438	microRNA 34a	MIR34A
439	claudin 18	CLDN18
440	polo like kinase 1	PLK1
441	AXL receptor tyrosine kinase	AXL
442	pregnancy specific beta-1-glycoprotein 2	PSG2
443	actin beta	ACTB
444	S100 calcium binding protein A9	S100A9
445	RNA binding motif protein 10	RBM10
446	RUNX family transcription factor 3	RUNX3
447	claudin 2	CLDN2
448	cyclin dependent kinase inhibitor 1A	CDKN1A
449	clusterin	CLU
450	dachshund family transcription factor 1	DACH1
451	E2F transcription factor 3	E2F3
452	eukaryotic translation initiation factor 4E	EIF4E
453	fibroblast growth factor 9	FGF9
454	glypican 5	GPC5
455	interleukin 17A	IL17A
456	indoleamine 2,3-dioxygenase 1	IDO1
457	keratin 19	KRT19
458	stathmin 1	STMN1
459	microRNA 126	MIR126

460	microRNA 150	MIR150
461	microRNA 155	MIR155
462	microRNA 198	MIR198
463	microRNA 205	MIR205
464	achaete-scute family bHLH transcription factor 1	ASCL1
465	forkhead box P3	FOXP3
466	pyruvate kinase M1/2	PKM
467	POU class 5 homeobox 1	POU5F1
468	mitogen-activated protein kinase 8	MAPK8
469	Rac family small GTPase 1	RAC1
470	C-C motif chemokine ligand 2	CCL2
471	surfactant protein B	SFTPB
472	BRCA1 DNA repair associated	BRCA1
473	signal transducer and activator of transcription 1	STAT1
474	aurora kinase A	AURKA
475	ADAM metallopeptidase domain 17	ADAM17
476	transforming growth factor beta receptor 2	TGFBR2
477	programmed cell death 1 ligand 2	PDCD1LG2
478	claudin 1	CLDN1
479	BCL2 like 11	BCL2L11
480	tripartite motif containing 28	TRIM28
481	centromere protein A	CENPA
482	insulin like growth factor 2 mRNA binding protein 1	IGF2BP1
483	antizyme inhibitor 2	AZIN2
484	cytotoxic T-lymphocyte associated protein 4	CTLA4
485	cytochrome P450 family 24 subfamily A member 1	CYP24A1
486	early growth response 1	EGR1
487	ERCC excision repair 2, TFIIH core complex helicase subunit	ERCC2
488	AKT serine/threonine kinase 2	AKT2
489	aldehyde dehydrogenase 1 family member A1	ALDH1A1
490	fibroblast growth factor 2	FGF2
491	dickkopf WNT signaling pathway inhibitor 1	DKK1
492	ubiquitin specific peptidase 22	USP22
493	sirtuin 1	SIRT1
494	GLI family zinc finger 1	GLI1
495	histone deacetylase 1	HDAC1
496	heme oxygenase 1	HMOX1
497	forkhead box A2	FOXA2
498	homeobox A10	HOXA10
499	homeobox B9	HOXB9
500	intercellular adhesion molecule 1	ICAM1
501	glutamate decarboxylase like 1	GADL1
502	interleukin 1 beta	IL1B
503	lin-28 homolog B	LIN28B

504	lipocalin 2	LCN2
505	mucin 21, cell surface associated	MUC21
506	microRNA 142	MIR142
507	microRNA 144	MIR144
508	microRNA 203a	MIR203A
509	microRNA 29b-1	MIR29B1
510	microRNA 29b-2	MIR29B2
511	microRNA 31	MIR31
512	microRNA 34b	MIR34B
513	epithelial cell adhesion molecule	EPCAM
514	SMAD family member 2	SMAD2
515	mesoderm specific transcript	MEST
516	O-6-methylguanine-DNA methyltransferase	MGMT
517	ATP binding cassette subfamily C member 1	ABCC1
518	macrophage scavenger receptor 1	MSR1
519	platelet and endothelial cell adhesion molecule 1	PECAM1
520	protein kinase C alpha	PRKCA
521	mitogen-activated protein kinase 3	MAPK3
522	aldo-keto reductase family 1 member B10	AKR1B10
523	C-C motif chemokine ligand 5	CCL5
524	endogenous retrovirus group K member 6, envelope	ERVK-6
525	surfactant protein A1	SFTPA1
526	SWI/SNF related, matrix associated, actin dependent regulator of chromatin, subfamily a, member 4	SMARCA4
527	microRNA 623	MIR623
528	transforming growth factor alpha	TGFA
529	TIMP metallopeptidase inhibitor 1	TIMP1
530	vascular endothelial growth factor C	VEGFC
531	ezrin	EZR
532	X-ray repair cross complementing 1	XRCC1
533	small cell cancer of the lung	SCLC1
534	caspase 8	CASP8
535	long intergenic non-protein coding RNA 467	LINC00467
536	hepatitis A virus cellular receptor 2	HAVCR2
537	family with sequence similarity 83 member A	FAM83A
538	methyl-CpG binding domain protein 2	MBD2
539	docking protein 2	DOK2
540	G protein-coupled receptor class C group 5 member A	GPRC5A
541	mucin 16, cell surface associated	MUC16
542	ATP binding cassette subfamily G member 2 (Junior blood group)	ABCG2
543	growth differentiation factor 15	GDF15
544	zinc finger E-box binding homeobox 2	ZEB2
545	TTN antisense RNA 1	TTN-AS1
546	long intergenic non-protein coding RNA 707	LINC00707

547	dynamin 1 like	DNM1L
548	cyclin dependent kinase inhibitor 1B	CDKN1B
549	APC regulator of WNT signaling pathway 2	APC2
550	protein arginine methyltransferase 5	PRMT5
551	peroxiredoxin 4	PRDX4
552	anterior gradient 2, protein disulphide isomerase family member	AGR2
553	solute carrier family 34 member 2	SLC34A2
554	periostin	POSTN
555	cleavage and polyadenylation specific factor 4	CPSF4
556	SUB1 regulator of transcription	SUB1
557	interleukin 24	IL24
558	cytoskeleton regulator RNA	CYTOR
559	ribosomal protein lateral stalk subunit P0 pseudogene 2	RPLP0P2
560	contactin 1	CNTN1
561	claudin 3	CLDN3
562	claudin 7	CLDN7
563	CRK like proto-oncogene, adaptor protein	CRKL
564	S100 calcium binding protein A16	S100A16
565	crystallin gamma D	CRYGD
566	adrenoceptor beta 2	ADRB2
567	FEZF1 antisense RNA 1	FEZF1-AS1
568	cytochrome P450 family 2 subfamily E member 1	CYP2E1
569	cytochrome P450 family 19 subfamily A member 1	CYP19A1
570	dihydropyrimidine dehydrogenase	DPYD
571	dual specificity phosphatase 6	DUSP6
572	nuclear receptor subfamily 0 group B member 1	NR0B1
573	enolase 2	ENO2
574	protein tyrosine kinase 2 beta	PTK2B
575	TPX2 microtubule nucleation factor	TPX2
576	TBC1 domain family member 9	TBC1D9
577	ABL proto-oncogene 1, non-receptor tyrosine kinase	ABL1
578	GATA binding protein 1	GATA1
579	interleukin 37	IL37
580	hematopoietic prostaglandin D synthase	HPGDS
581	ATPase family AAA domain containing 2	ATAD2
582	glycogen synthase kinase 3 beta	GSK3B
583	glutathione S-transferase theta 1	GSTT1
584	high mobility group box 1	HMGB1
585	high mobility group AT-hook 1	HMGA1
586	hyaluronan mediated motility receptor	HMMR
587	forkhead box A1	FOXA1
588	hepatocyte nuclear factor 4 alpha	HNF4A
589	homeobox A11	HOXA11
590	heat shock protein family A (Hsp70) member 5	HSPA5

591	baculoviral IAP repeat containing 5	BIRC5
592	inhibitor of DNA binding 1, HLH protein	ID1
593	interleukin 10	IL10
594	interleukin 11	IL11
595	interferon regulatory factor 3	IRF3
596	Janus kinase 1	JAK1
597	lysyl oxidase	LOX
598	microRNA 141	MIR141
599	microRNA 182	MIR182
600	microRNA 206	MIR206
601	microRNA 214	MIR214
602	microRNA 29c	MIR29C
603	microRNA 34c	MIR34C
604	microRNA 93	MIR93
605	microRNA 99a	MIR99A
606	MCL1 apoptosis regulator, BCL2 family member	MCL1
607	matrix metallopeptidase 12	MMP12
608	microRNA 326	MIR326
609	mucin 5AC, oligomeric mucus/gel-forming	MUC5AC
610	CEA cell adhesion molecule 6	CEACAM6
611	neural precursor cell expressed, developmentally down-regulated 9	NEDD9
612	notch receptor 2	NOTCH2
613	notch receptor 3	NOTCH3
614	receptor tyrosine kinase like orphan receptor 1	ROR1
615	oncostatin M	OSM
616	inhibitor of growth family member 4	ING4
617	progesterone receptor	PGR
618	ERBB receptor feedback inhibitor 1	ERRFI1
619	anillin actin binding protein	ANLN
620	protein phosphatase 2 phosphatase activator	PTPA
621	maternally expressed 3	MEG3
622	protein kinase C zeta	PRKCZ
623	transmembrane serine protease 4	TMPRSS4
624	solute carrier family 12 member 9	SLC12A9
625	S100 calcium binding protein A14	S100A14
626	microRNA 451a	MIR451A
627	microRNA 511	MIR511
628	protein tyrosine kinase 7 (inactive)	PTK7
629	cellular inhibitor of PP2A	CIP2A
630	paxillin	PXN
631	pyrroline-5-carboxylate reductase 1	PYCR1
632	RAD9 checkpoint clamp component A	RAD9A
633	MOK protein kinase	MOK
634	regenerating family member 1 alpha	REG1A

635	RELA proto-oncogene, NF-kB subunit	RELA
636	ATP binding cassette subfamily E member 1	ABCE1
637	microRNA 486-1	MIR486-1
638	S100 calcium binding protein A4	S100A4
639	syndecan 2	SDC2
640	POU class 5 homeobox 1 pseudogene 3	POU5F1P3
641	POU class 5 homeobox 1 pseudogene 4	POU5F1P4
642	golgi reassembly stacking protein 1	GORASP1
643	CUB domain containing protein 1	CDCP1
644	X antigen family member 1B	XAGE1B
645	X antigen family member 1A	XAGE1A
646	snail family transcriptional repressor 1	SNAI1
647	SRY-box transcription factor 5	SOX5
648	SRY-box transcription factor 9	SOX9
649	SRC proto-oncogene, non-receptor tyrosine kinase	SRC
650	basigin (Ok blood group)	BSG
651	synaptotagmin 1	SYT1
652	tafazzin	TAZ
653	microRNA 608	MIR608
654	transferrin receptor	TFRC
655	tumor protein p73	TP73
656	tumor protein, translationally-controlled 1	TPT1
657	surfactant protein A2	SFTPA2
658	ubiquitin conjugating enzyme E2 I	UBE2I
659	tyrosine 3-monooxygenase/tryptophan 5-monooxygenase activation protein zeta	YWHAZ
660	C-X-C motif chemokine receptor 4	CXCR4
661	CD276 molecule	CD276
662	kinesin family member 18A	KIF18A
663	Rho GTPase activating protein 24	ARHGAP24
664	caspase 9	CASP9
665	ADAM metallopeptidase domain 9	ADAM9
666	cholecystokinin	CCK
667	lysine acetyltransferase 2B	KAT2B
668	CD40 ligand	CD40LG
669	CD74 molecule	CD74
670	cell division cycle 20	CDC20
671	mitofusin 2	MFN2
672	major vault protein	MVP
673	N-acetyltransferase 2	NAT2
674	adenosine deaminase	ADA
675	cadherin 2	CDH2
676	HOX transcript antisense RNA	HOTAIR
677	microRNA 944	MIR944

678	CD24 molecule	CD24
679	Polymicrogyria, bilateral occipital	BOP
680	microRNA 1297	MIR1297
681	microRNA 1290	MIR1290
682	TMED7-TICAM2 readthrough	TMED7-TICAM2
683	long intergenic non-protein coding RNA 578	LINC00578
684	endogenous retrovirus group K member 20	ERVK-20
685	HECT, UBA and WWE domain containing E3 ubiquitin protein ligase 1	HUWE1
686	protein tyrosine phosphatase receptor type U	PTPRU
687	EGF like repeats and discoidin domains 3	EDIL3
688	cyclin dependent kinase 2	CDK2
689	flotillin 1	FLOT1
690	mesothelin	MSLN
691	cyclin dependent kinase 2 associated protein 2	CDK2AP2
692	ZFPM2 antisense RNA 1	ZFPM2-AS1
693	long intergenic non-protein coding RNA 1419	LINC01419
694	N-myc downstream regulated 1	NDRG1
695	transforming acidic coiled-coil containing protein 3	TACC3
696	calcium and integrin binding 1	CIB1
697	zinc ribbon domain containing 2	ZNRD2
698	mitochondrial ribosomal protein L28	MRPL28
699	sperm associated antigen 5	SPAG5
700	lung adenocarcinoma associated transcript 1	LUADT1
701	insulin like growth factor 2 mRNA binding protein 3	IGF2BP3
702	dynactin subunit 6	DCTN6
703	heparanase	HPSE
704	ADAM metallopeptidase domain 28	ADAM28
705	cytoskeleton associated protein 4	CKAP4
706	KCNQ1 opposite strand/antisense transcript 1	KCNQ1OT1
707	COP9 signalosome subunit 5	COPS5
708	endogenous retrovirus group K member 32, envelope	ERVK-32
709	SRY-box transcription factor 30	SOX30
710	ribonuclease P/MRP subunit p14	RPP14
711	serine/threonine kinase receptor associated protein	STRAP
712	checkpoint kinase 2	CHEK2
713	Ras association domain family member 8	RASSF8
714	Kruppel like factor 8	KLF8
715	chromobox 3	CBX3
716	cholinergic receptor nicotinic alpha 4 subunit	CHRNA4
717	NLR family pyrin domain containing 3	NLRP3
718	cytoglobin	CYGB
719	H3 histone pseudogene 9	H3P9
720	H3 histone pseudogene 8	H3P8

721	H3 histone pseudogene 23	H3P23
722	aneurysm, intracranial berry 1	ANIB1
723	C-C motif chemokine receptor 7	CCR7
724	collagen type I alpha 2 chain	COL1A2
725	Kruppel like factor 6	KLF6
726	solute carrier organic anion transporter family member 6A1	SLCO6A1
727	cytochrome c oxidase subunit 8A	COX8A
728	claudin 4	CLDN4
729	carbamoyl-phosphate synthase 1	CPS1
730	cellular retinoic acid binding protein 2	CRABP2
731	kinesin family member 18B	KIF18B
732	cancer/testis antigen 1B	CTAG1B
733	cellular communication network factor 2	CCN2
734	catenin delta 1	CTNND1
735	catenin delta 2	CTNND2
736	cathepsin D	CTSD
737	xyloside xylosyltransferase 1	XXYLT1
738	cytochrome P450 family 1 subfamily A member 2	CYP1A2
739	cytochrome P450 family 1 subfamily B member 1	CYP1B1
740	cytochrome P450 family 2 subfamily B member 6	CYP2B6
741	cell division cycle associated 2	CDCA2
742	FMR1 neighbor	FMR1NB
743	progestin and adipoQ receptor family member 7	PAQR7
744	damage specific DNA binding protein 1	DDB1
745	2,4-dienoyl-CoA reductase 1	DECR1
746	iodothyronine deiodinase 3	DIO3
747	DNA methyltransferase 1	DNMT1
748	atrophin 1	ATN1
749	dual specificity phosphatase 4	DUSP4
750	sphingosine-1-phosphate receptor 1	S1PR1
751	E74 like ETS transcription factor 3	ELF3
752	adenylate kinase 4	AK4
753	EPH receptor B6	EPHB6
754	erb-b2 receptor tyrosine kinase 4	ERBB4
755	estrogen related receptor beta	ESRRB
756	EYA transcriptional coactivator and phosphatase 2	EYA2
757	activated leukocyte cell adhesion molecule	ALCAM
758	F2R like trypsin receptor 1	F2RL1
759	aldehyde dehydrogenase 3 family member A1	ALDH3A1
760	flap structure-specific endonuclease 1	FEN1
761	vascular endothelial growth factor D	VEGFD
762	flotillin 2	FLOT2
763	jumonji domain containing 6, arginine demethylase and lysine hydroxylase	JMJD6
764	fms related receptor tyrosine kinase 4	FLT4

765	fibronectin 1	FN1
766	zinc finger protein, FOG family member 2	ZFPM2
767	folate receptor beta	FOLR2
768	preferentially expressed antigen in melanoma	PRAME
769	caspase 14	CASP14
770	solute carrier family 7 member 11	SLC7A11
771	cancer/testis antigen 1A	CTAG1A
772	fucosyltransferase 2	FUT2
773	fucosyltransferase 4	FUT4
774	surfactant associated 3	SFTA3
775	RAD54 homolog B	RAD54B
776	BRMS1 transcriptional repressor and anoikis regulator	BRMS1
777	abnormal spindle microtubule assembly	ASPM
778	WW domain containing transcription regulator 1	WWTR1
779	pre-mRNA processing factor 31	PRPF31
780	growth arrest specific 6	GAS6
781	DiGeorge syndrome critical region gene 5	DGCR5
782	phosphoglycerate dehydrogenase	PHGDH
783	GATA binding protein 6	GATA6
784	1,4-alpha-glucan branching enzyme 1	GBE1
785	gap junction protein alpha 1	GJA1
786	ATPase secretory pathway Ca2+ transporting 1	ATP2C1
787	gap junction protein beta 1	GJB1
788	dickkopf WNT signaling pathway inhibitor 3	DKK3
789	glypican 3	GPC3
790	TOX high mobility group box family member 3	TOX3
791	GLI family zinc finger 2	GLI2
792	angiogenin	ANG
793	nuclear paraspeckle assembly transcript 1	NEAT1
794	angiopoietin 1	ANGPT1
795	C-X-C motif chemokine ligand 17	CXCL17
796	sperm-tail PG-rich repeat containing 4	STPG4
797	repulsive guidance molecule BMP co-receptor b	RGMB
798	G protein-coupled receptor kinase 6	GRK6
799	glutathione peroxidase 2	GPX2
800	growth factor receptor bound protein 2	GRB2
801	tribbles pseudokinase 2	TRIB2
802	late endosomal/lysosomal adaptor, MAPK and MTOR activator 2	LAMTOR2
803	Kruppel like factor 15	KLF15
804	nuclear transport factor 2 like export factor 1	NXT1
805	ubiquitin like with PHD and ring finger domains 1	UHRF1
806	gastrin releasing peptide	GRP
807	gelsolin	GSN
808	ubiquilin 1	UBQLN1

809	granzyme B	GZMB
810	complement factor H related 1	CFHR1
811	endogenous retrovirus group W member 1, envelope	ERVW-1
812	celiac disease 2	CELIAC2
813	homeobox A9	HOXA9
814	homeobox B2	HOXB2
815	homeobox B3	HOXB3
816	homeobox B7	HOXB7
817	heat shock protein family A (Hsp70) member 4	HSPA4
818	X-linked inhibitor of apoptosis	XIAP
819	heat shock protein family B (small) member 2	HSPB2
820	hypertrichosis 2 (generalized, congenital)	HTC2
821	inhibitor of DNA binding 3, HLH protein	ID3
822	inhibitor of DNA binding 4, HLH protein	ID4
823	interferon alpha inducible protein 27	IFI27
824	apolipoprotein E	APOE
825	insulin like growth factor 2	IGF2
826	insulin like growth factor binding protein 3	IGFBP3
827	insulin like growth factor binding protein 7	IGFBP7
828	adenine phosphoribosyltransferase	APRT
829	toll like receptor adaptor molecule 2	TICAM2
830	Fas cell surface death receptor	FAS
831	inhibitor of nuclear factor kappa B kinase subunit beta	IKBKB
832	interleukin 2	IL2
833	interleukin 9	IL9
834	C-X-C motif chemokine receptor 2	CXCR2
835	interleukin 12 receptor subunit beta 2	IL12RB2
836	aquaporin 3 (Gill blood group)	AQP3
837	inhibin subunit beta A	INHBA
838	insulin receptor substrate 1	IRS1
839	androgen receptor	AR
840	Jun proto-oncogene, AP-1 transcription factor subunit	JUN
841	tripartite motif containing 23	TRIM23
842	glutathione S-transferase kappa 1	GSTK1
843	CD82 molecule	CD82
844	keratin 5	KRT5
845	lactate dehydrogenase B	LDHB
846	galectin 3	LGALS3
847	galectin 4	LGALS4
848	lysyl oxidase like 2	LOXL2
849	LIM domain containing preferred translocation partner in lipoma	LPP
850	long intergenic non-protein coding RNA 1194	LINC01194
851	lumican	LUM
852	microRNA let-7c	MIRLET7C

853	microRNA 100	MIR100
854	microRNA 106a	MIR106A
855	microRNA 134	MIR134
856	microRNA 136	MIR136
857	microRNA 137	MIR137
858	microRNA 140	MIR140
859	microRNA 143	MIR143
860	microRNA 146a	MIR146A
861	microRNA 15a	MIR15A
862	microRNA 17	MIR17
863	microRNA 186	MIR186
864	microRNA 192	MIR192
865	microRNA 204	MIR204
866	microRNA 22	MIR22
867	microRNA 221	MIR221
868	microRNA 222	MIR222
869	microRNA 224	MIR224
870	microRNA 33a	MIR33A
871	arrestin beta 1	ARRB1
872	minichromosome maintenance complex component 4	MCM4
873	menin 1	MEN1
874	microfibril associated protein 1	MFAP1
875	marker of proliferation Ki-67	MKI67
876	nuclear receptor subfamily 3 group C member 2	NR3C2
877	matrix metallopeptidase 10	MMP10
878	matrix metallopeptidase 11	MMP11
879	matrix metallopeptidase 14	MMP14
880	matrix metallopeptidase 15	MMP15
881	myeloperoxidase	MPO
882	long intergenic non-protein coding RNA 857	LINC00857
883	microRNA 135b	MIR135B
884	microRNA 339	MIR339
885	microRNA 372	MIR372
886	mutS homolog 2	MSH2
887	moesin	MSN
888	methylenetetrahydrofolate reductase	MTHFR
889	microsomal triglyceride transfer protein	MTTP
890	methylmalonyl-CoA mutase	MMUT
891	myosin binding protein H	MYBPH
892	MYCL proto-oncogene, bHLH transcription factor	MYCL
893	NADH:ubiquinone oxidoreductase subunit C2	NDUFC2
894	NEDD4 E3 ubiquitin protein ligase	NEDD4
895	SUMO1 pseudogene 3	SUMO1P3
896	NIMA related kinase 2	NEK2

897	neurofibromin 1	NF1
898	neurofibromin 2	NF2
899	Y-box binding protein 1	YBX1
900	neurotrophic receptor tyrosine kinase 1	NTRK1
901	neurotensin receptor 1	NTSR1
902	microRNA 375	MIR375
903	microRNA 381	MIR381
904	microRNA 423	MIR423
905	microRNA 425	MIR425
906	p21 (RAC1) activated kinase 1	PAK1
907	parkin RBR E3 ubiquitin protein ligase	PRKN
908	taste 2 receptor member 13	TAS2R13
909	F11 receptor	F11R
910	transmembrane p24 trafficking protein 7	TMED7
911	splicing factor 3b subunit 6	SF3B6
912	GINS complex subunit 2	GINS2
913	pyruvate dehydrogenase kinase 4	PDK4
914	solute carrier family 25 member 3	SLC25A3
915	serpin family A member 1	SERPINA1
916	prolactin induced protein	PIP
917	phospholipase A2 group IIA	PLA2G2A
918	LDL receptor related protein 1B	LRP1B
919	syntrophin gamma 1	SNTG1
920	RNA binding motif protein 47	RBM47
921	IMP U3 small nucleolar ribonucleoprotein 3	IMP3
922	MIR31 host gene	MIR31HG
923	microRNA 429	MIR429
924	protein kinase AMP-activated catalytic subunit alpha 1	PRKAA1
925	protein kinase AMP-activated catalytic subunit alpha 2	PRKAA2
926	protein kinase AMP-activated non-catalytic subunit beta 1	PRKAB1
927	protein kinase cAMP-dependent type I regulatory subunit alpha	PRKAR1A
928	phosphoprotein membrane anchor with glycosphingolipid microdomains 1	PAG1
929	protein kinase C iota	PRKCI
930	actin related protein 10	ACTR10
931	PDZ binding kinase	PBK
932	LIM domain only 3	LMO3
933	myeloid derived growth factor	MYDGF
934	brain expressed X-linked 4	BEX4
935	C-C motif chemokine ligand 28	CCL28
936	resistin	RETN
937	atypical chemokine receptor 3	ACKR3
938	poly(rC) binding protein 4	PCBP4
939	proteasome 26S subunit, non-ATPase 2	PSMD2
940	proteasome 26S subunit, non-ATPase 9	PSMD9

941	actin related protein 3B	ACTR3B
942	microRNA 363	MIR363
943	parathyroid hormone like hormone	PTHLH
944	protein tyrosine kinase 2	PTK2
945	pleiotrophin	PTN
946	tribbles pseudokinase 3	TRIB3
947	protein tyrosine phosphatase receptor type G	PTPRG
948	Rac family small GTPase 3	RAC3
949	RAD52 homolog, DNA repair protein	RAD52
950	RNA binding motif protein 3	RBM3
951	opsin 1, long wave sensitive	OPN1LW
952	radixin	RDX
953	RELB proto-oncogene, NF-kB subunit	RELB
954	Ras like without CAAX 1	RIT1
955	Rho associated coiled-coil containing protein kinase 1	ROCK1
956	retinoid isomerohydrolase RPE65	RPE65
957	brain derived neurotrophic factor	BDNF
958	S100 calcium binding protein A2	S100A2
959	S100 calcium binding protein B	S100B
960	secretin	SCT
961	C-C motif chemokine ligand 19	CCL19
962	C-X-C motif chemokine ligand 5	CXCL5
963	PR/SET domain 16	PRDM16
964	selectin P	SELP
965	secreted frizzled related protein 1	SFRP1
966	serine and arginine rich splicing factor 1	SRSF1
967	interleukin 25	IL25
968	S-phase kinase associated protein 2	SKP2
969	MARCKS like 1	MARCKSL1
970	Fanconi renotubular syndrome	FRTS1
971	actin related protein 3C	ACTR3C
972	SWI/SNF related, matrix associated, actin dependent regulator of chromatin, subfamily a, member 1	SMARCA1
973	SWI/SNF related, matrix associated, actin dependent regulator of chromatin, subfamily a, member 2	SMARCA2
974	serine/threonine kinase 33	STK33
975	synuclein alpha	SNCA
976	sterol O-acyltransferase 1	SOAT1
977	SRY-box transcription factor 4	SOX4
978	Sp1 transcription factor	SP1
979	serine peptidase inhibitor Kazal type 1	SPINK1
980	serum response factor	SRF
981	signal transducer and activator of transcription 6	STAT6
982	spleen associated tyrosine kinase	SYK

983	transgelin	TAGLN
984	microRNA 411	MIR411
985	T cell receptor beta variable 20/OR9-2 (non-functional)	TRBV20OR9-2
986	transcription factor AP-2 alpha	TFAP2A
987	thrombomodulin	THBD
988	thrombospondin 1	THBS1
989	TIAM Rac1 associated GEF 1	TIAM1
990	TIMP metallopeptidase inhibitor 2	TIMP2
991	TLE family member 1, transcriptional corepressor	TLE1
992	toll like receptor 4	TLR4
993	TNF receptor associated factor 6	TRAF6
994	TSC complex subunit 1	TSC1
995	TSC complex subunit 2	TSC2
996	mucin 5B, oligomeric mucus/gel-forming	MUC5B
997	long intergenic non-protein coding RNA 460	LINC00460
998	small ubiquitin like modifier 1	SUMO1
999	secretoglobin family 1A member 1	SCGB1A1
1000	UDP glucuronosyltransferase family 2 member B17	UGT2B17
1001	ubiquitin specific peptidase 4	USP4
1002	UV radiation resistance associated	UVRAG
1003	vasoactive intestinal peptide receptor 1	VIPR1
1004	X inactive specific transcript	XIST
1005	ZFP36 ring finger protein	ZFP36
1006	discoidin domain receptor tyrosine kinase 1	DDR1
1007	WD repeat domain 77	WDR77
1008	basic helix-loop-helix family member e41	BHLHE41
1009	calbindin 2	CALB2
1010	centromere protein U	CENPU
1011	Nanog homeobox	NANOG
1012	Wnt ligand secretion mediator	WLS
1013	coiled-coil domain containing 6	CCDC6
1014	tet methylcytosine dioxygenase 1	TET1
1015	PPP1R2C family member C	PPP1R2C
1016	FOS like 1, AP-1 transcription factor subunit	FOSL1
1017	collagen type XVIII alpha 1 chain	COL18A1
1018	high mobility group AT-hook 2	HMGA2
1019	actinin alpha 4	ACTN4
1020	nectin cell adhesion molecule 4	NECTIN4
1021	MIA SH3 domain containing	MIA
1022	nuclear receptor coactivator 3	NCOA3
1023	sestrin 2	SESN2
1024	phospholipase A2 group X	PLA2G10
1025	speckle type BTB/POZ protein	SPOP

1026	HORMA domain containing 1	HORMAD1
1027	trichoplein keratin filament binding	TCHP
1028	HOP homeobox	HOPX
1029	PHD finger protein 5A	PHF5A
1030	R-spondin 3	RSPO3
1031	major facilitator superfamily domain containing 2A	MFSD2A
1032	tankyrase	TNKS
1033	beclin 1	BECN1
1034	ATP binding cassette subfamily C member 3	ABCC3
1035	membrane bound transcription factor peptidase, site 1	MBTPS1
1036	delta like non-canonical Notch ligand 1	DLK1
1037	Wnt family member 3A	WNT3A
1038	heat shock protein family B (small) member 3	HSPB3
1039	selenium binding protein 1	SELENBP1
1040	suppressor of cytokine signaling 3	SOCS3
1041	protein regulator of cytokinesis 1	PRC1
1042	claudin 10	CLDN10
1043	ubiquitin specific peptidase 14	USP14
1044	ubiquitin specific peptidase 8	USP8
1045	regucalcin	RGN
1046	programmed cell death 5	PDCD5
1047	Kruppel like factor 4	KLF4
1048	slit guidance ligand 2	SLIT2
1049	adiponectin, C1Q and collagen domain containing	ADIPOQ
1050	CD80 molecule	CD80
1051	QKI, KH domain containing RNA binding	QKI
1052	mitogen-activated protein kinase kinase kinase kinase 4	MAP4K4
1053	CD34 molecule	CD34
1054	CD68 molecule	CD68
1055	histone deacetylase 9	HDAC9
1056	cyclin dependent kinase 1	CDK1
1057	cadherin 3	CDH3
1058	LIN28B antisense RNA 1	LIN28B-AS1
1059	microRNA 888	MIR888
1060	microRNA 873	MIR873
1061	microRNA 216b	MIR216B
1062	microRNA 938	MIR938
1063	microRNA 708	MIR708
1064	microRNA 297	MIR297
1065	microRNA 365a	MIR365A
1066	TMPO antisense RNA 1	TMPO-AS1
1067	FAM83H antisense RNA 1 (head to head)	FAM83H-AS1
1068	MIR29B2 and MIR29C host gene	MIR29B2CHG
1069	SOD2 overlapping transcript 1	SOD2-OT1

1070	histone deacetylase 6	HDAC6
1071	FAM83A antisense RNA 1	FAM83A-AS1
1072	inhibitory synaptic factor family member 2B	INSYN2B
1073	ACTA2 antisense RNA 1	ACTA2-AS1
1074	WASH and IL9R antisense RNA 2	WASIR2
1075	long intergenic non-protein coding RNA 1512	LINC01512
1076	HOXA cluster antisense RNA 3	HOXA-AS3
1077	programmed cell death 6	PDCD6
1078	Trichoepithelioma, multiple familial, 2	MFT2
1079	trophinin associated protein	TROAP
1080	long intergenic non-protein coding RNA 941	LINC00941
1081	LOXL1 antisense RNA 1	LOXL1-AS1
1082	microRNA 1286	MIR1286
1083	microRNA 1275	MIR1275
1084	microRNA 1914	MIR1914
1085	microRNA 1205	MIR1205
1086	microRNA 1827	MIR1827
1087	microRNA 1293	MIR1293
1088	microRNA 1236	MIR1236
1089	PCNA antisense RNA 1	PCNA-AS1
1090	HOXA distal transcript antisense RNA	HOTTIP
1091	microRNA 3941	MIR3941
1092	microRNA 3662	MIR3662
1093	microRNA 3613	MIR3613
1094	NKX2-1 antisense RNA 1	NKX2-1-AS1
1095	HOXA transcript antisense RNA, myeloid-specific 1	HOTAIRM1
1096	occludin	OCLN
1097	prostate cancer associated transcript 6	PCAT6
1098	long intergenic non-protein coding RNA 1354	LINC01354
1099	long intergenic non-protein coding RNA 665	LINC00665
1100	MHC class I polypeptide-related sequence A	MICA
1101	SOX21 antisense divergent transcript 1	SOX21-AS1
1102	long intergenic non-protein coding RNA 968	LINC00968
1103	RPL17-C18orf32 readthrough	RPL17-C18orf32
1104	ubiquitin like modifier activating enzyme 2	UBA2
1105	microRNA 4513	MIR4513
1106	microRNA 4677	MIR4677
1107	microRNA 4746	MIR4746
1108	nuclear receptor subfamily 1 group H member 3	NR1H3
1109	SPRY4 intronic transcript 1	SPRY4-IT1
1110	microRNA 5587	MIR5587
1111	fibrinogen silencer binding protein	FSBP
1112	HOXA10 antisense RNA	HOXA10-AS

1113	kinesin family member 20A	KIF20A
1114	NPTN intronic transcript 1	NPTN-IT1
1115	leucine rich pentatricopeptide repeat containing	LRPPRC
1116	PDZK1 interacting protein 1	PDZK1IP1
1117	bladder cancer associated transcript 1	BLACAT1
1118	teneurin transmembrane protein 1	TENM1
1119	colon cancer associated transcript 2	CCAT2
1120	HMMR antisense RNA 1	HMMR-AS1
1121	teashirt zinc finger homeobox 1	TSHZ1
1122	cyclin dependent kinase 5	CDK5
1123	tripartite motif containing 13	TRIM13
1124	cyclin dependent kinase 6	CDK6
1125	gephyrin	GPHN
1126	Rab9 effector protein with kelch motifs	RABEPK
1127	microRNA 6839	MIR6839
1128	microRNA 6077	MIR6077
1129	microRNA 6783	MIR6783
1130	iroquois homeobox 5	IRX5
1131	salt inducible kinase 1B (putative)	SIK1B
1132	adenosine deaminase RNA specific	ADAR
1133	LanC like 1	LANCL1
1134	cyclin dependent kinase inhibitor 3	CDKN3
1135	DLC1 Rho GTPase activating protein	DLC1
1136	adenosine deaminase RNA specific B1	ADARB1
1137	interferon induced transmembrane protein 3	IFITM3
1138	craniofacial development protein 1	CFDP1
1139	C-type lectin domain containing 10A	CLEC10A
1140	follistatin	FST
1141	homeobox B13	HOXB13
1142	CCAAT enhancer binding protein alpha	CEBPA
1143	semaphorin 4B	SEMA4B
1144	CCAAT enhancer binding protein beta	CEBPB
1145	amyloid beta precursor protein binding protein 2	APPBP2
1146	long intergenic non-protein coding RNA 2310	LINC02310
1147	long intergenic non-protein coding RNA 1703	LINC01703
1148	long intergenic non-protein coding RNA 1614	LINC01614
1149	HIV-1 Tat interactive protein 2	HTATIP2
1150	olfactomedin 4	OLFM4
1151	C-X-C motif chemokine ligand 13	CXCL13
1152	SIVA1 apoptosis inducing factor	SIVA1
1153	chaperonin containing TCP1 subunit 4	CCT4
1154	poly(A) binding protein interacting protein 1	PAIP1
1155	centromere protein E	CENPE
1156	RNA, 7SL, cytoplasmic 494, pseudogene	RN7SL494P

1157	KH RNA binding domain containing, signal transduction associated 1	KHDRBS1
1158	C-X-C motif chemokine receptor 6	CXCR6
1159	delta like canonical Notch ligand 3	DLL3
1160	cofilin 1	CFL1
1161	DNA polymerase theta	POLQ
1162	prostaglandin E synthase 3	PTGES3
1163	polo like kinase 4	PLK4
1164	SIX homeobox 2	SIX2
1165	mitogen-activated protein kinase kinase kinase 2	MAP3K2
1166	cell adhesion molecule L1 like	CHL1
1167	nestin	NES
1168	long intergenic non-protein coding RNA 1852	LINC01852
1169	CF transmembrane conductance regulator	CFTR
1170	gap junction protein beta 6	GJB6
1171	protein phosphatase 1 regulatory subunit 17	PPP1R17
1172	progesterone receptor membrane component 1	PGRMC1
1173	leukocyte immunoglobulin like receptor B1	LILRB1
1174	HLA complex P5	HCP5
1175	neuromedin U	NMU
1176	WD repeat domain 3	WDR3
1177	PPARG coactivator 1 alpha	PPARGC1A
1178	MALT1 paracaspase	MALT1
1179	urotensin 2	UTS2
1180	ectodysplasin A receptor	EDAR
1181	ralA binding protein 1	RALBP1
1182	peroxiredoxin 3	PRDX3
1183	BTG anti-proliferation factor 3	BTG3
1184	small nucleolar RNA, C/D box 138	SNORD138
1185	stress induced phosphoprotein 1	STIP1
1186	EBNA1 binding protein 2	EBNA1BP2
1187	transmembrane p24 trafficking protein 10	TMED10
1188	fermitin family member 2	FERMT2
1189	COP9 signalosome subunit 6	COPS6
1190	inner membrane mitochondrial protein	IMMT
1191	kinesin family member 2C	KIF2C
1192	GLI pathogenesis related 1	GLIPR1
1193	receptor interacting serine/threonine kinase 3	RIPK3
1194	WW domain containing E3 ubiquitin protein ligase 2	WWP2
1195	chromodomain helicase DNA binding protein 4	CHD4
1196	protein tyrosine phosphatase receptor type T	PTPRT
1197	HERV-H LTR-associating 2	HHLA2
1198	SRY-box transcription factor 21	SOX21
1199	plakophilin 3	PKP3
1200	polypeptide N-acetylgalactosaminyltransferase 6	GALNT6

1201	egl-9 family hypoxia inducible factor 2	EGLN2
1202	caveolae associated protein 3	CAVIN3
1203	MUC5B antisense RNA 1	MUC5B-AS1
1204	ubiquitin specific peptidase 18	USP18
1205	interleukin 17F	IL17F
1206	long intergenic non-protein coding RNA 2605	LINC02605
1207	Parkinsonism associated deglycase	PARK7
1208	V-set and immunoglobulin domain containing 4	VSIG4
1209	Opa interacting protein 5	OIP5
1210	monoglyceride lipase	MGLL
1211	solute carrier family 35 member A4	SLC35A4
1212	cholinergic receptor nicotinic alpha 7 subunit	CHRNA7
1213	MIR155 host gene	MIR155HG
1214	component of inhibitor of nuclear factor kappa B kinase complex	CHUK
1215	cytokine inducible SH2 containing protein	CISH
1216	H3 histone pseudogene 17	H3P17
1217	H3 histone pseudogene 28	H3P28
1218	collagen triple helix repeat containing 1	CTHRC1
1219	basic leucine zipper ATF-like transcription factor 2	BATF2
1220	forkhead box P4	FOXP4
1221	secretoglobin family 3A member 2	SCGB3A2
1222	chloride voltage-gated channel 3	CLCN3
1223	ANTXR cell adhesion molecule 2	ANTXR2
1224	leucine rich repeat kinase 2	LRRK2
1225	chymase 1	CMA1
1226	tudor domain containing 9	TDRD9
1227	adenylosuccinate synthase 1	ADSS1
1228	perilipin 2	PLIN2
1229	C-C motif chemokine receptor 3	CCR3
1230	C-C motif chemokine receptor 4	CCR4
1231	C-C motif chemokine receptor 5 (gene/pseudogene)	CCR5
1232	C-C motif chemokine receptor 6	CCR6
1233	musashi RNA binding protein 2	MSI2
1234	alcohol dehydrogenase 1C (class I), gamma polypeptide	ADH1C
1235	cannabinoid receptor 1	CNR1
1236	ciliary neurotrophic factor receptor	CNTFR
1237	diencephalon/mesencephalon homeobox 1	DMBX1
1238	collagen type I alpha 1 chain	COL1A1
1239	long intergenic non-protein coding RNA 628	LINC00628
1240	collagen type III alpha 1 chain	COL3A1
1241	Kruppel like factor 17	KLF17
1242	chromosome 20 open reading frame 85	C20orf85
1243	collagen type V alpha 1 chain	COL5A1
1244	TATA-box binding protein associated factor 8	TAF8

1245	collagen type X alpha 1 chain	COL10A1
1246	collagen type XI alpha 1 chain	COL11A1
1247	odd-skipped related transcription factor 1	OSR1
1248	OCIA domain containing 2	OCIAD2
1249	mitogen-activated protein kinase kinase kinase 8	MAP3K8
1250	cytochrome c oxidase subunit 7A1	COX7A1
1251	adenosine A2a receptor	ADORA2A
1252	ceruloplasmin	CP
1253	carboxypeptidase A3	CPA3
1254	carboxypeptidase M	CPM
1255	coproporphyrinogen oxidase	CPOX
1256	cAMP responsive element binding protein 1	CREB1
1257	collapsin response mediator protein 1	CRMP1
1258	eukaryotic translation initiation factor 5A like 1	EIF5AL1
1259	colony stimulating factor 1 receptor	CSF1R
1260	layilin	LAYN
1261	E2F transcription factor 7	E2F7
1262	long intergenic non-protein coding RNA 355	LINC00355
1263	cystatin SN	CST1
1264	cystatin E/M	CST6
1265	zinc finger protein 560	ZNF560
1266	adrenoceptor alpha 1A	ADRA1A
1267	zinc finger protein 569	ZNF569
1268	C-terminal binding protein 1	CTBP1
1269	interleukin 23 receptor	IL23R
1270	chitinase, acidic pseudogene 2	CHIAP2
1271	salt inducible kinase 1	SIK1
1272	cytoskeleton associated protein 2 like	CKAP2L
1273	ITPRIP like 1	ITPRIPL1
1274	adrenoceptor alpha 2B	ADRA2B
1275	cathepsin E	CTSE
1276	phospholipase B1	PLB1
1277	regulator of microtubule dynamics 2	RMDN2
1278	cathepsin L	CTSL
1279	cut like homeobox 1	CUX1
1280	CXADR Ig-like cell adhesion molecule	CXADR
1281	shisa family member 3	SHISA3
1282	adrenoceptor beta 1	ADRB1
1283	CYLD lysine 63 deubiquitinase	CYLD
1284	long intergenic non-protein coding RNA 1600	LINC01600
1285	angiomotin	AMOT
1286	phosphatidylethanolamine binding protein 4	PEBP4
1287	cytochrome P450 family 3 subfamily A member 4	CYP3A4
1288	cytochrome P450 family 3 subfamily A member 5	CYP3A5

1289	Cbl proto-oncogene like 2	CBLL2
1290	DAB adaptor protein 2	DAB2
1291	diacylglycerol kinase alpha	DGKA
1292	solute carrier family 5 member 8	SLC5A8
1293	G protein-coupled receptor 180	GPR180
1294	death associated protein	DAP
1295	C-type lectin domain containing 14A	CLEC14A
1296	death associated protein kinase 1	DAPK1
1297	chromosome 18 open reading frame 54	C18orf54
1298	deoxycytidine kinase	DCK
1299	decorin	DCN
1300	SAS-6 centriolar assembly protein	SASS6
1301	aldo-keto reductase family 1 member C2	AKR1C2
1302	growth arrest and DNA damage inducible alpha	GADD45A
1303	TLE family member 5, transcriptional modulator	TLE5
1304	DExH-box helicase 9	DHX9
1305	DEAH-box helicase 15	DHX15
1306	DEAD-box helicase 53	DDX53
1307	zinc finger protein 384	ZNF384
1308	deoxyguanosine kinase	DGUOK
1309	dihydrofolate reductase	DHFR
1310	dihydrolipoamide dehydrogenase	DLD
1311	DNA methyltransferase 3 beta	DNMT3B
1312	dipeptidyl peptidase 4	DPP4
1313	angiotensinogen	AGT
1314	desmoglein 3	DSG3
1315	angiotensin II receptor type 1	AGTR1
1316	E2F transcription factor 4	E2F4
1317	ATP binding cassette subfamily A member 1	ABCA1
1318	lysophosphatidic acid receptor 1	LPAR1
1319	sphingosine-1-phosphate receptor 3	S1PR3
1320	endothelin 3	EDN3
1321	endothelin receptor type A	EDNRA
1322	endothelin receptor type B	EDNRB
1323	eukaryotic translation elongation factor 1 alpha 2	EEF1A2
1324	ephrin A1	EFNA1
1325	ephrin B2	EFNB2
1326	multiple EGF like domains 8	MEGF8
1327	phospholipid phosphatase 4	PLPP4
1328	EPH receptor A2	EPHA2
1329	eukaryotic translation initiation factor 4E binding protein 1	EIF4EBP1
1330	eukaryotic translation initiation factor 4 gamma 2	EIF4G2
1331	eukaryotic translation initiation factor 5	EIF5
1332	eukaryotic translation initiation factor 5A	EIF5A

1333	elastase, neutrophil expressed	ELANE
1334	tetratricopeptide repeat domain 21A	TTC21A
1335	long intergenic non-protein coding RNA 466	LINC00466
1336	ETS transcription factor ELK1	ELK1
1337	gastrokine 2	GKN2
1338	lipase H	LIPH
1339	emerin	EMD
1340	sterile alpha motif domain containing 14	SAMD14
1341	RAD52 motif containing 1	RDM1
1342	cortactin	CTTN
1343	empty spiracles homeobox 2	EMX2
1344	KH RNA binding domain containing, signal transduction associated 2	KHDRBS2
1345	adenylate kinase 1	AK1
1346	tubulin beta class I	TUBB
1347	serine protease 55	PRSS55
1348	vacuolar ATPase assembly factor VMA21	VMA21
1349	adenylate kinase 2	AK2
1350	EPH receptor A1	EPHA1
1351	EPH receptor A4	EPHA4
1352	erythropoietin receptor	EPOR
1353	nuclear receptor subfamily 2 group F member 6	NR2F6
1354	epiregulin	EREG
1355	EYA transcriptional coactivator and phosphatase 4	EYA4
1356	surfactant associated 1, lncRNA	SFTA1P
1357	ATP binding cassette subfamily A member 3	ABCA3
1358	estrogen related receptor alpha	ESRRA
1359	ETS proto-oncogene 1, transcription factor	ETS1
1360	ETS proto-oncogene 2, transcription factor	ETS2
1361	ETS variant transcription factor 4	ETV4
1362	ETS variant transcription factor 5	ETV5
1363	coagulation factor III, tissue factor	F3
1364	fatty acid amide hydrolase	FAAH
1365	fatty acid binding protein 4	FABP4
1366	aldehyde dehydrogenase 2 family member	ALDH2
1367	FA complementation group C	FANCC
1368	mediator complex subunit 19	MED19
1369	enkurin, TRPC channel interacting protein	ENKUR
1370	fibulin 2	FBLN2
1371	aldehyde dehydrogenase 1 family member A3	ALDH1A3
1372	Fc fragment of IgE receptor Ig	FCER1G
1373	alkB homolog 3, alpha-ketoglutaratedependent dioxygenase	ALKBH3
1374	HOXA11 antisense RNA	HOXA11-AS
1375	family with sequence similarity 83 member B	FAM83B
1376	FER tyrosine kinase	FER

1377	fibrinogen alpha chain	FGA
1378	fibroblast growth factor 4	FGF4
1379	fibrinogen like 1	FGL1
1380	fragile histidine triad diadenosine triphosphatase	FHIT
1381	four and a half LIM domains 1	FHL1
1382	integrin subunit alpha 11	ITGA11
1383	vasohibin 1	VASH1
1384	NLR family pyrin domain containing 1	NLRP1
1385	SEC31 homolog A, COPII coat complex component	SEC31A
1386	zinc fingers and homeoboxes 2	ZHX2
1387	calsyntenin 1	CLSTN1
1388	RUN and FYVE domain containing 3	RUFY3
1389	multimerin 1	MMRN1
1390	activating transcription factor 6	ATF6
1391	selenophosphate synthetase 2	SEPHS2
1392	RAB3 GTPase activating protein catalytic subunit 1	RAB3GAP1
1393	forkhead box C1	FOXC1
1394	apoptotic chromatin condensation inducer 1	ACIN1
1395	Fas apoptotic inhibitory molecule 2	FAIM2
1396	lysine demethylase 4B	KDM4B
1397	PH domain and leucine rich repeat protein phosphatase 2	PHLPP2
1398	WD and tetratricopeptide repeats 1	WDTC1
1399	golgi associated, gamma adaptin ear containing, ARF binding protein 2	GGA2
1400	senataxin	SETX
1401	endoplasmic reticulum protein 44	ERP44
1402	forkhead box O1	FOXO1
1403	signal induced proliferation associated 1 like 3	SIPA1L3
1404	filamin A	FLNA
1405	lipin 1	LPIN1
1406	disco interacting protein 2 homolog A	DIP2A
1407	F-box and leucine rich repeat protein 7	FBXL7
1408	sulfatase 1	SULF1
1409	fms related receptor tyrosine kinase 3	FLT3
1410	nucleoporin 210	NUP210
1411	PH domain and leucine rich repeat protein phosphatase 1	PHLPP1
1412	OTU deubiquitinase 3	OTUD3
1413	WW and C2 domain containing 1	WWC1
1414	F-box and WD repeat domain containing 11	FBXW11
1415	ATM interactor	ATMIN
1416	Sad1 and UNC84 domain containing 1	SUN1
1417	zinc finger protein 629	ZNF629
1418	UFM1 specific ligase 1	UFL1
1419	dicer 1, ribonuclease III	DICER1
1420	sirtuin 3	SIRT3

1421	solute carrier family 44 member 1	SLC44A1
1422	folate hydrolase 1	FOLH1
1423	cardiotrophin like cytokine factor 1	CLCF1
1424	Fos proto-oncogene, AP-1 transcription factor subunit	FOS
1425	FosB proto-oncogene, AP-1 transcription factor subunit	FOSB
1426	folylpolyglutamate synthase	FPGS
1427	dimethylarginine dimethylaminohydrolase 1	DDAH1
1428	opsin 3	OPN3
1429	Cbl proto-oncogene C	CBLC
1430	LDOC1 regulator of NFKB signaling	LDOC1
1431	small nucleolar RNA host gene 1	SNHG1
1432	SH3 domain binding protein 4	SH3BP4
1433	arachidonate 5-lipoxygenase activating protein	ALOX5AP
1434	kinesin family member 4A	KIF4A
1435	claudin 15	CLDN15
1436	fragile site, aphidicolin type, common, fra(16)(q23.2)	FRA16D
1437	arachidonate 15-lipoxygenase type B	ALOX15B
1438	frizzled related protein	FRZB
1439	FUS RNA binding protein	FUS
1440	fucosyltransferase 1 (H blood group)	FUT1
1441	fucosyltransferase 3 (Lewis blood group)	FUT3
1442	RPTOR independent companion of MTOR complex 2	RICTOR
1443	glucose-6-phosphate dehydrogenase	G6PD
1444	cancer susceptibility 2	CASC2
1445	gamma-aminobutyric acid type A receptor subunit alpha3	GABRA3
1446	proprotein convertase subtilisin/kexin type 9	PCSK9
1447	glycerol kinase 5	GK5
1448	glutamate decarboxylase 1	GAD1
1449	SAM pointed domain containing ETS transcription factor	SPDEF
1450	kallikrein related peptidase 5	KLK5
1451	ariadne RBR E3 ubiquitin protein ligase 1	ARIH1
1452	mitochondrial tRNA translation optimization 1	MTO1
1453	anti-silencing function 1A histone chaperone	ASF1A
1454	armadillo repeat containing 8	ARMC8
1455	tripartite motif containing 58	TRIM58
1456	polypeptide N-acetylgalactosaminyltransferase 3	GALNT3
1457	chloride intracellular channel 4	CLIC4
1458	SAM and HD domain containing deoxynucleoside triphosphate triphosphohydrolase 1	SAMHD1
1459	nectin cell adhesion molecule 3	NECTIN3
1460	adhesion G protein-coupled receptor A2	ADGRA2
1461	SH2B adaptor protein 1	SH2B1
1462	EGF like domain multiple 6	EGFL6
1463	RNA exonuclease 2	REXO2

1464	golgi reassembly stacking protein 2	GORASP2
1465	acyl-CoA thioesterase 11	ACOT11
1466	Adolescent idiopathic scoliosis	IS1
1467	DDB1 and CUL4 associated factor 4	DCAF4
1468	GATA binding protein 2	GATA2
1469	GATA binding protein 4	GATA4
1470	F-box protein 22	FBXO22
1471	MYC binding protein	MYCBP
1472	guanylate binding protein 1	GBP1
1473	gastrulation brain homeobox 2	GBX2
1474	LIM homeobox 6	LHX6
1475	pleckstrin 2	PLEK2
1476	regulator of G protein signaling 17	RGS17
1477	growth differentiation factor 2	GDF2
1478	gremlin 1, DAN family BMP antagonist	GREM1
1479	variable charge X-linked	VCX
1480	GTP binding protein overexpressed in skeletal muscle	GEM
1481	beta-1,4-galactosyltransferase 1	B4GALT1
1482	growth hormone receptor	GHR
1483	gastric inhibitory polypeptide	GIP
1484	ABL proto-oncogene 2, non-receptor tyrosine kinase	ABL2
1485	fibroblast growth factor 22	FGF22
1486	forkhead box D3	FOXD3
1487	NADPH oxidase 1	NOX1
1488	linker for activation of T cells	LAT
1489	UTP25 small subunit processor component	UTP25
1490	staphylococcal nuclease and tudor domain containing 1	SND1
1491	lysosomal associated membrane protein 3	LAMP3
1492	MDM2 binding protein	MTBP
1493	dickkopf WNT signaling pathway inhibitor 4	DKK4
1494	dickkopf WNT signaling pathway inhibitor 2	DKK2
1495	AF4/FMR2 family member 4	AFF4
1496	argonaute RISC catalytic component 2	AGO2
1497	interleukin 17C	IL17C
1498	interleukin 17B	IL17B
1499	galactosidase beta 1	GLB1
1500	glutamate-cysteine ligase catalytic subunit	GCLC
1501	ubiquitin conjugating enzyme E2 S	UBE2S
1502	pre-mRNA processing factor 19	PRPF19
1503	RAB guanine nucleotide exchange factor 1	RABGEF1
1504	malonyl-CoA-acyl carrier protein transacylase	MCAT
1505	small G protein signaling modulator 3	SGSM3
1506	glutaminase	GLS
1507	glutaredoxin	GLRX

1508	amylase alpha 1A	AMY1A
1509	GDP-mannose 4,6-dehydratase	GMDS
1510	amylase alpha 1B	AMY1B
1511	amylase alpha 1C	AMY1C
1512	glutamic-oxaloacetic transaminase 1	GOT1
1513	glypican 1	GPC1
1514	SBF2 antisense RNA 1	SBF2-AS1
1515	H19 imprinted maternally expressed transcript	H19
1516	long intergenic non-protein coding RNA 485	LINC00485
1517	HNF1A antisense RNA 1	HNF1A-AS1
1518	FBXL19 antisense RNA 1	FBXL19-AS1
1519	long intergenic non-protein coding RNA 324	LINC00324
1520	laminin subunit alpha 1	LAMA1
1521	LY6/PLAUR domain containing 5	LYPD5
1522	zinc finger protein 763	ZNF763
1523	WD repeat domain 62	WDR62
1524	R-spondin 1	RSPO1
1525	long intergenic non-protein coding RNA 1270	LINC01270
1526	long intergenic non-protein coding RNA 319	LINC00319
1527	delta like canonical Notch ligand 1	DLL1
1528	tumor suppressor candidate 7	TUSC7
1529	coiled-coil domain containing 66	CCDC66
1530	DLX6 antisense RNA 1	DLX6-AS1
1531	transmembrane p24 trafficking protein 10 pseudogene 1	TMED10P1
1532	G protein-coupled receptor 42 (gene/pseudogene)	GPR42
1533	tripartite motif containing 59	TRIM59
1534	glutathione peroxidase 4	GPX4
1535	MCTS1 re-initiation and release factor	MCTS1
1536	nuclear receptor subfamily 3 group C member 1	NR3C1
1537	syntaxin binding protein 6	STXBP6
1538	drosha ribonuclease III	DROSHA
1539	bromodomain containing 7	BRD7
1540	glutathione-disulfide reductase	GSR
1541	glutathione S-transferase mu 2	GSTM2
1542	glutathione S-transferase mu 4	GSTM4
1543	glutathione S-transferase pi 1	GSTP1
1544	ubiquitin specific peptidase 25	USP25
1545	Obg like ATPase 1	OLA1
1546	inducible T cell costimulator	ICOS
1547	guanylate kinase 1	GUK1
1548	glucuronidase beta	GUSB
1549	eukaryotic elongation factor 2 kinase	EEF2K
1550	regulator of MON1-CCZ1	RMC1
1551	long intergenic non-protein coding RNA 312	LINC00312

1552	mitochondrial rRNA methyltransferase 2	MRM2
1553	LDL receptor related protein 12	LRP12
1554	T-box transcription factor 21	TBX21
1555	sperm protein associated with the nucleus, X-linked, family member A1	SPANXA1
1556	hemoglobin subunit epsilon 1	HBE1
1557	histone deacetylase 2	HDAC2
1558	heparin binding growth factor	HDGF
1559	complement factor H	CFH
1560	annexin A6	ANXA6
1561	HIC ZBTB transcriptional repressor 1	HIC1
1562	huntingtin interacting protein 1	HIP1
1563	major histocompatibility complex, class II, DQ beta 1	HLA-DQB1
1564	high mobility group box 3	HMGB3
1565	apoptotic peptidase activating factor 1	APAF1
1566	heterogeneous nuclear ribonucleoprotein D	HNRNPD
1567	heterogeneous nuclear ribonucleoprotein K	HNRNPK
1568	homeobox A cluster	HOXA@
1569	homeobox A3	HOXA3
1570	homeobox A4	HOXA4
1571	homeobox A5	HOXA5
1572	homeobox A13	HOXA13
1573	homeobox B4	HOXB4
1574	homeobox C10	HOXC10
1575	homeobox C13	HOXC13
1576	homeobox D cluster	HOXD@
1577	haptoglobin	HP
1578	hypoxanthine phosphoribosyltransferase 1	HPRT1
1579	heat shock protein family A (Hsp70) member 1A	HSPA1A
1580	heat shock protein family A (Hsp70) member 1B	HSPA1B
1581	heat shock protein family A (Hsp70) member 8	HSPA8
1582	heat shock protein family D (Hsp60) member 1	HSPD1
1583	keratin associated protein 8-1	KRTAP8-1
1584	S100 calcium binding protein A7A	S100A7A
1585	interferon regulatory factor 8	IRF8
1586	armadillo like helical domain containing 1	ARMH1
1587	R-spondin 2	RSPO2
1588	brain expressed X-linked 5	BEX5
1589	V-set and immunoglobulin domain containing 1	VSIG1
1590	isocitrate dehydrogenase (NAD(+)) 3 catalytic subunit alpha	IDH3A
1591	complement factor I	CFI
1592	interferon induced protein with tetratricopeptide repeats 2	IFIT2
1593	interferon alpha 2	IFNA2
1594	interferon alpha and beta receptor subunit 1	IFNAR1
1595	RNA binding motif protein Y-linked family 2 member D, pseudogene	RBMY2DP

1596	immunoglobulin binding protein 1	IGBP1
1597	SOX2 overlapping transcript	SOX2-OT
1598	serine incorporator 2	SERINC2
1599	Prader Willi/Angelman region RNA 4	PWAR4
1600	insulin like growth factor binding protein 2	IGFBP2
1601	insulin like growth factor binding protein 4	IGFBP4
1602	amyloid beta precursor protein	APP
1603	immunoglobulin kappa constant	IGKC
1604	kallikrein related peptidase 3	KLK3
1605	interleukin 2 receptor subunit alpha	IL2RA
1606	interleukin 4	IL4
1607	interleukin 7	IL7
1608	interleukin 7 receptor	IL7R
1609	aquaporin 1 (Colton blood group)	AQP1
1610	aquaporin 5	AQP5
1611	C-X-C motif chemokine ligand 10	CXCL10
1612	insulin like 4	INSL4
1613	insulin receptor	INSR
1614	integrin subunit alpha 6	ITGA6
1615	interferon regulatory factor 1	IRF1
1616	interferon stimulated exonuclease gene 20	ISG20
1617	integrin subunit beta 1	ITGB1
1618	integrin subunit beta 2	ITGB2
1619	inositol-trisphosphate 3-kinase A	ITPKA
1620	jagged canonical Notch ligand 2	JAG2
1621	JunD proto-oncogene, AP-1 transcription factor subunit	JUND
1622	amphiregulin	AREG
1623	family with sequence similarity 111 member B	FAM111B
1624	chromosome 5 open reading frame 34	C5orf34
1625	agrin	AGRN
1626	potassium two pore domain channel subfamily K member 3	KCNK3
1627	potassium calcium-activated channel subfamily M alpha 1	KCNMA1
1628	potassium voltage-gated channel subfamily Q member 1	KCNQ1
1629	RNA binding motif protein Y-linked family 1 member D	RBMY1D
1630	kinesin family member 2A	KIF2A
1631	KIT proto-oncogene, receptor tyrosine kinase	KIT
1632	kininogen 1	KNG1
1633	karyopherin subunit beta 1	KPNB1
1634	keratin 6A	KRT6A
1635	keratin 6B	KRT6B
1636	keratin 16	KRT16
1637	small nucleolar RNA host gene 5	SNHG5
1638	long intergenic non-protein coding RNA 222	LINC00222
1639	keratin 17	KRT17

1640	transmembrane protein 189	TMEM189
1641	TMEM189-UBE2V1 readthrough	TMEM189-UBE2V1
1642	ras homolog family member B	RHOB
1643	C-C motif chemokine ligand 4 like 1	CCL4L1
1644	calpain 8	CAPN8
1645	ladinin 1	LAD1
1646	acetyl-CoA acetyltransferase 2	ACAT2
1647	laminin subunit alpha 2	LAMA2
1648	laminin subunit alpha 3	LAMA3
1649	laminin subunit gamma 2	LAMC2
1650	LCK proto-oncogene, Src family tyrosine kinase	LCK
1651	lactate dehydrogenase C	LDHC
1652	low density lipoprotein receptor	LDLR
1653	leptin receptor	LEPR
1654	Rho GDP dissociation inhibitor alpha	ARHGDIA
1655	LIF receptor subunit alpha	LIFR
1656	long intergenic non-protein coding RNA 2167	LINC02167
1657	FOXF1 adjacent non-coding developmental regulatory RNA	FENDRR
1658	HOXD antisense growth-associated long non-coding RNA	HAGLR
1659	loricrin cornified envelope precursor protein	LORICRIN
1660	lysyl oxidase like 1	LOXL1
1661	lipoprotein(a)	LPA
1662	lipoprotein lipase	LPL
1663	lymphoid restricted membrane protein	LRMP
1664	LDL receptor related protein 1	LRP1
1665	limbic system associated membrane protein	LSAMP
1666	lymphotoxin alpha	LTA
1667	LYN proto-oncogene, Src family tyrosine kinase	LYN
1668	microRNA let-7e	MIRLET7E
1669	microRNA let-7f-1	MIRLET7F1
1670	microRNA 122	MIR122
1671	microRNA 127	MIR127
1672	microRNA 130a	MIR130A
1673	microRNA 139	MIR139
1674	microRNA 148a	MIR148A
1675	microRNA 149	MIR149
1676	microRNA 154	MIR154
1677	microRNA 15b	MIR15B
1678	microRNA 191	MIR191
1679	microRNA 193a	MIR193A
1680	microRNA 197	MIR197
1681	microRNA 20a	MIR20A
1682	microRNA 200c	MIR200C

1683	microRNA 216a	MIR216A
1684	microRNA 217	MIR217
1685	arrestin 3	ARR3
1686	tumor associated calcium signal transducer 2	TACSTD2
1687	microRNA 218-1	MIR218-1
1688	microRNA 223	MIR223
1689	microRNA 23b	MIR23B
1690	microRNA 25	MIR25
1691	microRNA 27a	MIR27A
1692	microRNA 29a	MIR29A
1693	microRNA 296	MIR296
1694	microRNA 30c-2	MIR30C2
1695	microRNA 30e	MIR30E
1696	microRNA 320a	MIR320A
1697	microRNA 95	MIR95
1698	microRNA 96	MIR96
1699	microRNA 98	MIR98
1700	mitotic arrest deficient 2 like 1	MAD2L1
1701	arrestin beta 2	ARRB2
1702	SMAD family member 5	SMAD5
1703	SMAD family member 6	SMAD6
1704	MAF bZIP transcription factor G	MAFG
1705	MAGE family member A2	MAGEA2
1706	microtubule associated protein 4	MAP4
1707	myoglobin	MB
1708	minichromosome maintenance complex component 2	MCM2
1709	minichromosome maintenance complex component 5	MCM5
1710	CD46 molecule	CD46
1711	midkine	MDK
1712	MDM4 regulator of p53	MDM4
1713	mitogen-activated protein kinase kinase kinase 3	MAP3K3
1714	melanotransferrin	MELTF
1715	secretoglobin family 2A member 2	SCGB2A2
1716	KIT ligand	KITLG
1717	microsomal glutathione S-transferase 1	MGST1
1718	midline 1	MID1
1719	major intrinsic protein of lens fiber	MIP
1720	matrix metallopeptidase 1	MMP1
1721	matrix metallopeptidase 7	MMP7
1722	matrix metallopeptidase 13	MMP13
1723	molybdenum cofactor synthesis 2	MOCS2
1724	CD200 molecule	CD200
1725	mannose receptor C-type 1	MRC1
1726	PRDM16 divergent transcript	PRDM16-DT

1727	zyg-11 family member A, cell cycle regulator	ZYG11A
1728	myocardial infarction associated transcript	MIAT
1729	fatty acyl-CoA reductase 2 pseudogene 1	FAR2P1
1730	basic charge Y-linked 2B	BPY2B
1731	basic charge Y-linked 2C	BPY2C
1732	microRNA 148b	MIR148B
1733	microRNA 338	MIR338
1734	microRNA 340	MIR340
1735	microRNA 370	MIR370
1736	microRNA 373	MIR373
1737	microRNA 374a	MIR374A
1738	aspartate beta-hydroxylase	ASPH
1739	macrophage stimulating 1	MST1
1740	metallothionein 1G	MT1G
1741	nudix hydrolase 1	NUDT1
1742	NADH dehydrogenase, subunit 5 (complex I)	ND5
1743	NADH dehydrogenase, subunit 6 (complex I)	ND6
1744	mucin 6, oligomeric mucus/gel-forming	MUC6
1745	mutY DNA glycosylase	MUTYH
1746	MX dynamin like GTPase 2	MX2
1747	MYB proto-oncogene like 2	MYBL2
1748	MYCN proto-oncogene, bHLH transcription factor	MYCN
1749	MYD88 innate immune signal transduction adaptor	MYD88
1750	growth arrest and DNA damage inducible beta	GADD45B
1751	myogenic differentiation 1	MYOD1
1752	activating transcription factor 4	ATF4
1753	nibrin	NBN
1754	neural cell adhesion molecule 1	NCAM1
1755	nuclear cap binding protein subunit 1	NCBP1
1756	ATP citrate lyase	ACLY
1757	developmentally regulated GTP binding protein 1	DRG1
1758	NEDD8 ubiquitin like modifier	NEDD8
1759	neural EGFL like 2	NELL2
1760	neogenin 1	NEO1
1761	neuronal differentiation 1	NEUROD1
1762	nuclear factor of activated T cells 2	NFATC2
1763	nuclear factor, interleukin 3 regulated	NFIL3
1764	nuclear factor I X	NFIX
1765	neuronatin	NNAT
1766	NME/NM23 nucleoside diphosphate kinase 1	NME1
1767	nitric oxide synthase 2	NOS2
1768	nitric oxide synthase 3	NOS3
1769	neurotensin	NTS
1770	CDGSH iron sulfur domain 2	CISD2

1771	microRNA 361	MIR361
1772	microRNA 378a	MIR378A
1773	microRNA 379	MIR379
1774	microRNA 382	MIR382
1775	microRNA 383	MIR383
1776	OCA2 melanosomal transmembrane protein	OCA2
1777	ornithine decarboxylase 1	ODC1
1778	oxidized low density lipoprotein receptor 1	OLR1
1779	opioid binding protein/cell adhesion molecule like	OPCML
1780	SIX homeobox 6	SIX6
1781	orosomucoid 1	ORM1
1782	orthodenticle homeobox 2	OTX2
1783	pyrimidinergic receptor P2Y6	P2RY6
1784	RGMB antisense RNA 1	RGMB-AS1
1785	phosphatidylethanolamine binding protein 1	PEBP1
1786	progestagen associated endometrial protein	PAEP
1787	platelet activating factor acetylhydrolase 1b regulatory subunit 1	PAFAH1B1
1788	NADPH oxidase 4	NOX4
1789	deleted in esophageal cancer 1	DELEC1
1790	peroxiredoxin 1	PRDX1
1791	serpin family E member 1	SERPINE1
1792	serpin family B member 2	SERPINB2
1793	interleukin 22	IL22
1794	gem nuclear organelle associated protein 4	GEMIN4
1795	peptidylglycine alpha-amidating monooxygenase	PAM
1796	pro-apoptotic WT1 regulator	PAWR
1797	paired box 6	PAX6
1798	T cell receptor associated transmembrane adaptor 1	TRAT1
1799	familial cortical myoclonic tremor with epilepsy 1	FCMTE1
1800	protocadherin 7	PCDH7
1801	NADH:ubiquinone oxidoreductase subunit A13	NDUFA13
1802	SH3 domain containing GRB2 like, endophilin B1	SH3GLB1
1803	proliferating cell nuclear antigen	PCNA
1804	regulator of microtubule dynamics 1	RMDN1
1805	pericentrin	PCNT
1806	heat shock protein family A (Hsp70) member 14	HSPA14
1807	CDKN2A divergent transcript	CDKN2A-DT
1808	cysteine rich transmembrane BMP regulator 1	CRIM1
1809	MAPKAPK5 antisense RNA 1	MAPKAPK5-AS1
1810	toll like receptor 7	TLR7
1811	ATP synthase F1 subunit delta	ATP5F1D
1812	golgi associated kinase 1B	GASK1B
1813	placenta associated 8	PLAC8

1814	phosducin	PDC
1815	programmed cell death 2	PDCD2
1816	zinc finger and BTB domain containing 7A	ZBTB7A
1817	WT1 antisense RNA	WT1-AS
1818	ubiquitin C-terminal hydrolase L5	UCHL5
1819	phosphodiesterase 3A	PDE3A
1820	phosphodiesterase 4D	PDE4D
1821	inositol-3-phosphate synthase 1	ISYNA1
1822	variable charge X-linked 3A	VCX3A
1823	denticleless E3 ubiquitin protein ligase homolog	DTL
1824	anaphase promoting complex subunit 11	ANAPC11
1825	vesicle trafficking 1	VTA1
1826	sirtuin 6	SIRT6
1827	glycerophosphodiester phosphodiesterase 1	GDE1
1828	platelet derived growth factor receptor beta	PDGFRB
1829	ERGIC and golgi 3	ERGIC3
1830	pyruvate dehydrogenase kinase 1	PDK1
1831	pyruvate dehydrogenase kinase 2	PDK2
1832	peptidyl-tRNA hydrolase 2	PTRH2
1833	mitochondrial pyruvate carrier 1	MPC1
1834	cleavage and polyadenylation specific factor 3	CPSF3
1835	SIX homeobox 4	SIX4
1836	6-phosphofructo-2-kinase/fructose-2,6-biphosphatase 3	PFKFB3
1837	profilin 1	PFN1
1838	Pim-1 proto-oncogene, serine/threonine kinase	PIM1
1839	peptidylprolyl cis/trans isomerase, NIMA-interacting 1	PIN1
1840	paired like homeodomain 1	PITX1
1841	paired like homeodomain 2	PITX2
1842	polycystin 2, transient receptor potential cation channel	PKD2
1843	PBX/knotted 1 homeobox 1	PKNOX1
1844	plakophilin 2	PKP2
1845	phospholipase A2 group IB	PLA2G1B
1846	PLAG1 like zinc finger 2	PLAGL2
1847	plasminogen activator, urokinase receptor	PLAUR
1848	BAF chromatin remodeling complex subunit BCL11A	BCL11A
1849	interleukin 17D	IL17D
1850	plectin	PLEC
1851	procollagen-lysine,2-oxoglutarate 5-dioxygenase 2	PLOD2
1852	plastin 1	PLS1
1853	PTOV1 extended AT-hook containing adaptor protein	PTOV1
1854	phosphomannomutase 2	PMM2
1855	acid phosphatase 5, tartrate resistant	ACP5
1856	receptor interacting serine/threonine kinase 4	RIPK4
1857	toll like receptor 9	TLR9

1858	podocalyxin like	PODXL
1859	DNA polymerase epsilon 2, accessory subunit	POLE2
1860	SRY-box transcription factor 18	SOX18
1861	TERF2 interacting protein	TERF2IP
1862	proline rich 13	PRR13
1863	keratin 20	KRT20
1864	POU class 2 homeobox associating factor 1	POU2AF1
1865	POU class 2 homeobox 2	POU2F2
1866	POU class 3 homeobox 2	POU3F2
1867	DNA damage inducible transcript 4	DDIT4
1868	erythrocyte membrane protein band 4.1 like 4B	EPB41L4B
1869	egl-9 family hypoxia inducible factor 1	EGLN1
1870	inorganic pyrophosphatase 1	PPA1
1871	UDP glucuronosyltransferase family 1 member A3	UGT1A3
1872	transmembrane protein 106B	TMEM106B
1873	cardiolipin synthase 1	CRLS1
1874	mediator complex subunit 1	MED1
1875	XIAP associated factor 1	XAF1
1876	cathepsin A	CTSA
1877	peptidylprolyl isomerase A	PPIA
1878	tRNA isopentenyltransferase 1	TRIT1
1879	zinc finger CCHC-type containing 10	ZCCHC10
1880	epithelial splicing regulatory protein 1	ESRP1
1881	alkB homolog 5, RNA demethylase	ALKBH5
1882	non-SMC condensin II complex subunit G2	NCAPG2
1883	ring finger protein 43	RNF43
1884	YTH N6-methyladenosine RNA binding protein 1	YTHDF1
1885	tescalcin	TESC
1886	taurine up-regulated 1	TUG1
1887	ANKRD40 C-terminal like	ANKRD40CL
1888	solute carrier family 52 member 1	SLC52A1
1889	protein phosphatase 1 regulatory subunit 8	PPP1R8
1890	protein phosphatase 2 catalytic subunit alpha	PPP2CA
1891	centrosomal protein 55	CEP55
1892	regulator of microtubule dynamics 3	RMDN3
1893	ATPase family AAA domain containing 3A	ATAD3A
1894	proton activated chloride channel 1	PACC1
1895	cancer susceptibility 1	CASC1
1896	lysosomal protein transmembrane 4 beta	LAPTM4B
1897	minichromosome maintenance 10 replication initiation factor	MCM10
1898	familial cortical myoclonic tremor with epilepsy 2	FCMTE2
1899	microRNA 448	MIR448
1900	microRNA 449a	MIR449A
1901	serglycin	SRGN

1902	SRY-box transcription factor 6	SOX6
1903	SPT20 homolog, SAGA complex component	SUPT20H
1904	zinc finger CCHC-type containing 8	ZCCHC8
1905	fermitin family member 1	FERMT1
1906	kinesin family member 16B	KIF16B
1907	ArfGAP with SH3 domain, ankyrin repeat and PH domain 3	ASAP3
1908	DEP domain containing 1	DEPDC1
1909	syntabulin	SYBU
1910	zinc finger protein 692	ZNF692
1911	hypoxia inducible factor 1 subunit alpha inhibitor	HIF1AN
1912	centrosomal protein 72	CEP72
1913	protein kinase C delta	PRKCD
1914	lysine demethylase 3A	KDM3A
1915	ELL associated factor 2	EAF2
1916	acyl-CoA thioesterase 13	ACOT13
1917	brain expressed X-linked 1	BEX1
1918	protein kinase D1	PRKD1
1919	regulator of chromosome condensation 2	RCC2
1920	sulfatase 2	SULF2
1921	mitogen-activated protein kinase 4	MAPK4
1922	mitogen-activated protein kinase 9	MAPK9
1923	mitogen-activated protein kinase kinase 3	MAP2K3
1924	protocadherin gamma subfamily B, 6	PCDHGB6
1925	prolactin	PRL
1926	prion protein	PRNP
1927	zinc finger protein 253	ZNF253
1928	KIAA1217	KIAA1217
1929	gastrokine 1	GKN1
1930	alpha-2-glycoprotein 1, zinc-binding	AZGP1
1931	methyltransferase like 3	METTL3
1932	serine protease 3	PRSS3
1933	HtrA serine peptidase 1	HTRA1
1934	proteinase 3	PRTN3
1935	pleckstrin and Sec7 domain containing	PSD
1936	proteasome 20S subunit alpha 7	PSMA7
1937	latexin	LXN
1938	prostate transmembrane protein, androgen induced 1	PMEPA1
1939	aryl hydrocarbon receptor nuclear translocator like 2	ARNTL2
1940	mitochondrial fission factor	MFF
1941	OTU deubiquitinase 7B	OTUD7B
1942	FAM20C golgi associated secretory pathway kinase	FAM20C
1943	kinesin family member 15	KIF15
1944	choline phosphotransferase 1	CHPT1
1945	catenin beta interacting protein 1	CTNNBIP1

1946	PEST proteolytic signal containing nuclear protein	PCNP
1947	TP53 induced glycolysis regulatory phosphatase	TIGAR
1948	calcium/calmodulin dependent protein kinase ID	CAMK1D
1949	golgi associated PDZ and coiled-coil motif containing	GOPC
1950	proteasome 26S subunit, non-ATPase 7	PSMD7
1951	secreted LY6/PLAUR domain containing 1	SLURP1
1952	pellino E3 ubiquitin protein ligase 1	PELI1
1953	adhesion G protein-coupled receptor G6	ADGRG6
1954	TP73 antisense RNA 1	TP73-AS1
1955	differentiation antagonizing non-protein coding RNA	DANCR
1956	BAG cochaperone 1	BAG1
1957	activation induced cytidine deaminase	AICDA
1958	ras homolog family member J	RHOJ
1959	microRNA 20b	MIR20B
1960	microRNA 18b	MIR18B
1961	SPC25 component of NDC80 kinetochore complex	SPC25
1962	microRNA 452	MIR452
1963	microRNA 409	MIR409
1964	microRNA 410	MIR410
1965	microRNA 485	MIR485
1966	microRNA 432	MIR432
1967	microRNA 496	MIR496
1968	microRNA 497	MIR497
1969	microRNA 519b	MIR519B
1970	microRNA 520c	MIR520C
1971	microRNA 519d	MIR519D
1972	microRNA 499a	MIR499A
1973	microRNA 503	MIR503
1974	microRNA 506	MIR506
1975	solute carrier family 12 member 5	SLC12A5
1976	extended synaptotagmin 2	ESYT2
1977	AT-rich interaction domain 1B	ARID1B
1978	myocardin related transcription factor B	MRTFB
1979	kinase D interacting substrate 220	KIDINS220
1980	G protein-coupled receptor 158	GPR158
1981	mindbomb E3 ubiquitin protein ligase 1	MIB1
1982	TAO kinase 1	TAOK1
1983	twinfilin actin binding protein 1	TWF1
1984	synaptotagmin 13	SYT13
1985	SH3 domain containing ring finger 1	SH3RF1
1986	cell cycle and apoptosis regulator 2	CCAR2
1987	protein tyrosine phosphatase non-receptor type 13	PTPN13
1988	protein tyrosine phosphatase receptor type B	PTPRB
1989	protein tyrosine phosphatase receptor type C	PTPRC

1990	protein tyrosine phosphatase receptor type H	PTPRH
1991	BCL2 associated X, apoptosis regulator	BAX
1992	PVR cell adhesion molecule	PVR
1993	ovo like zinc finger 2	OVOL2
1994	RAB5A, member RAS oncogene family	RAB5A
1995	RAB27B, member RAS oncogene family	RAB27B
1996	RAD51 recombinase	RAD51
1997	actin alpha 2, smooth muscle	ACTA2
1998	butyrylcholinesterase	BCHE
1999	RAP1A, member of RAS oncogene family	RAP1A
2000	Ras protein specific guanine nucleotide releasing factor 1	RASGRF1
2001	angiotensin I converting enzyme 2	ACE2
2002	RB binding protein 8, endonuclease	RBBP8
2003	RB transcriptional corepressor like 2	RBL2
2004	leucine rich repeat containing G protein-coupled receptor 6	LGR6
2005	RNA binding motif protein Y-linked family 1 member A1	RBMY1A1
2006	recoverin	RCVRN
2007	regenerating family member 1 beta	REG1B
2008	replication factor C subunit 3	RFC3
2009	BCL2 like 2	BCL2L2
2010	RNA component of mitochondrial RNA processing endoribonuclease	RMRP
2011	solute carrier family 5 member 7	SLC5A7
2012	potassium two pore domain channel subfamily K member 15	KCNK15
2013	migration and invasion inhibitory protein	MIIP
2014	growth arrest specific 5	GAS5
2015	small nucleolar RNA, C/D box 15A	SNORD15A
2016	TNF receptor superfamily member 17	TNFRSF17
2017	roundabout guidance receptor 1	ROBO1
2018	ribosomal protein L17	RPL17
2019	ribosomal protein L19	RPL19
2020	ribosomal protein S6	RPS6
2021	ribosomal protein S6 kinase A1	RPS6KA1
2022	thyroid carcinoma, Hurthle cell	HCC
2023	microRNA 483	MIR483
2024	small nucleolar RNA, H/ACA box 41	SNORA41
2025	ribosomal protein S6 kinase B1	RPS6KB1
2026	ribosomal protein S15a	RPS15A
2027	ribosomal protein S19	RPS19
2028	bradykinin receptor B1	BDKRB1
2029	RRAD, Ras related glycolysis inhibitor and calcium channel regulator	RRAD
2030	RAS related	RRAS
2031	ras responsive element binding protein 1	RREB1
2032	bradykinin receptor B2	BDKRB2
2033	rhotekin	RTKN

2034	S100 calcium binding protein A1	S100A1
2035	S100 calcium binding protein A7	S100A7
2036	S100 calcium binding protein A8	S100A8
2037	S100 calcium binding protein A11	S100A11
2038	S100 calcium binding protein P	S100P
2039	ataxin 1	ATXN1
2040	serpin family B member 3	SERPINB3
2041	serpin family B member 4	SERPINB4
2042	stearoyl-CoA desaturase	SCD
2043	biglycan	BGN
2044	C-C motif chemokine ligand 1	CCL1
2045	C-C motif chemokine ligand 4	CCL4
2046	C-C motif chemokine ligand 18	CCL18
2047	C-C motif chemokine ligand 20	CCL20
2048	C-C motif chemokine ligand 21	CCL21
2049	BCL2 interacting killer	BIK
2050	syndecan binding protein	SDCBP
2051	C-X-C motif chemokine ligand 12	CXCL12
2052	HEAT repeat containing 6	HEATR6
2053	NOP2/Sun RNA methyltransferase 3	NSUN3
2054	PR/SET domain 1	PRDM1
2055	cell death inducing DFFA like effector c	CIDEC
2056	kinesin family member 13A	KIF13A
2057	p53 apoptosis effector related to PMP22	PERP
2058	cadherin related 23	CDH23
2059	tenomodulin	TNMD
2060	V-set immunoregulatory receptor	VSIR
2061	selenoprotein P	SELENOP
2062	non-SMC condensin I complex subunit G	NCAPG
2063	mitogen-activated protein kinase kinase 4	MAP2K4
2064	small nucleolar RNA host gene 6	SNHG6
2065	SET nuclear proto-oncogene	SET
2066	serine and arginine rich splicing factor 2	SRSF2
2067	nuclear receptor binding SET domain protein 1	NSD1
2068	colorectal neoplasia differentially expressed	CRNDE
2069	surfactant protein D	SFTPD
2070	autophagy related 3	ATG3
2071	inverted formin, FH2 and WH2 domain containing	INF2
2072	serum/glucocorticoid regulated kinase 1	SGK1
2073	small glutamine rich tetratricopeptide repeat containing alpha	SGTA
2074	cytoplasmic polyadenylation element binding protein 1	CPEB1
2075	long intergenic non-protein coding RNA 607	LINC00607
2076	IGF2BP2 antisense RNA 1	IGF2BP2-AS1
2077	SPANX family member C	SPANXC

2078	SET and MYND domain containing 3	SMYD3
2079	fibronectin type III domain containing 3B	FNDC3B
2080	siah E3 ubiquitin protein ligase 2	SIAH2
2081	non-coding RNA activated by DNA damage	NORAD
2082	DEP domain containing MTOR interacting protein	DEPTOR
2083	purinergic receptor P2Y12	P2RY12
2084	SIM bHLH transcription factor 1	SIM1
2085	SIX homeobox 1	SIX1
2086	SIX homeobox 3	SIX3
2087	SKI proto-oncogene	SKI
2088	mitochondrial ribosomal protein L41	MRPL41
2089	bone morphogenetic protein 2	BMP2
2090	S-phase kinase associated protein 1	SKP1
2091	PTEN induced kinase 1	PINK1
2092	reticulon 4 receptor	RTN4R
2093	solute carrier family 1 member 5	SLC1A5
2094	solute carrier family 2 member 3	SLC2A3
2095	solute carrier family 2 member 5	SLC2A5
2096	bone morphogenetic protein 4	BMP4
2097	solute carrier family 3 member 2	SLC3A2
2098	solute carrier family 5 member 2	SLC5A2
2099	solute carrier family 5 member 5	SLC5A5
2100	cytosolic arginine sensor for mTORC1 subunit 1	CASTOR1
2101	CXADR pseudogene 1	CXADRP1
2102	zymogen granule protein 16	ZG16
2103	solute carrier family 11 member 1	SLC11A1
2104	solute carrier family 12 member 1	SLC12A1
2105	solute carrier family 12 member 2	SLC12A2
2106	solute carrier family 12 member 3	SLC12A3
2107	solute carrier family 16 member 1	SLC16A1
2108	slit guidance ligand 3	SLIT3
2109	sarcolipin	SLN
2110	snail family transcriptional repressor 2	SNAI2
2111	BMX non-receptor tyrosine kinase	BMX
2112	Ly6/neurotoxin 1	LYNX1
2113	SWI/SNF related, matrix associated, actin dependent regulator of chromatin, subfamily d, member 1	SMARCD1
2114	microRNA 545	MIR545
2115	superoxide dismutase 1	SOD1
2116	BCL2 family apoptosis regulator BOK	BOK
2117	SRY-box transcription factor 10	SOX10
2118	sperm associated antigen 4	SPAG4
2119	secreted protein acidic and cysteine rich	SPARC
2120	SPG7 matrix AAA peptidase subunit, paraplegin	SPG7

2121	SPARC (osteonectin), cwcv and kazal like domains proteoglycan 1	SPOCK1
2122	small proline rich protein 1B	SPRR1B
2123	bactericidal permeability increasing protein	BPI
2124	spectrin beta, non-erythrocytic 1	SPTBN1
2125	SRSF protein kinase 1	SRPK1
2126	SRSF protein kinase 2	SRPK2
2127	somatostatin receptor 4	SSTR4
2128	signal transducer and activator of transcription 2	STAT2
2129	signal transducer and activator of transcription 5A	STAT5A
2130	signal transducer and activator of transcription 5B	STAT5B
2131	stanniocalcin 1	STC1
2132	bombesin receptor subtype 3	BRS3
2133	vesicle associated membrane protein 2	VAMP2
2134	T-box transcription factor 2	TBX2
2135	T-box transcription factor 5	TBX5
2136	transcription factor 4	TCF4
2137	microRNA 33b	MIR33B
2138	microRNA 532	MIR532
2139	microRNA 552	MIR552
2140	microRNA 576	MIR576
2141	microRNA 584	MIR584
2142	microRNA 589	MIR589
2143	microRNA 590	MIR590
2144	microRNA 592	MIR592
2145	microRNA 605	MIR605
2146	microRNA 620	MIR620
2147	microRNA 622	MIR622
2148	microRNA 629	MIR629
2149	microRNA 630	MIR630
2150	transcription factor 21	TCF21
2151	TEA domain transcription factor 4	TEAD4
2152	transcription factor A, mitochondrial	TFAM
2153	transcription factor AP-2 beta	TFAP2B
2154	transcription factor AP-2 gamma	TFAP2C
2155	transcription factor Dp-1	TFDP1
2156	transcription factor Dp-2	TFDP2
2157	trefoil factor 1	TFF1
2158	trefoil factor 3	TFF3
2159	thyroglobulin	TG
2160	transforming growth factor beta induced	TGFBI
2161	thrombospondin 2	THBS2
2162	Thy-1 cell surface antigen	THY1
2163	Kruppel like factor 10	KLF10
2164	tight junction protein 1	TJP1

2165	transmembrane 4 L six family member 4	TM4SF4
2166	transmembrane 7 superfamily member 2	TM7SF2
2167	tropomodulin 1	TMOD1
2168	claudin 5	CLDN5
2169	TNF receptor superfamily member 1A	TNFRSF1A
2170	DNA topoisomerase II beta	TOP2B
2171	DNA topoisomerase III alpha	TOP3A
2172	tumor protein p53 binding protein 1	TP53BP1
2173	tropomyosin 3	TPM3
2174	thyroid peroxidase	TPO
2175	cysteine rich secretory protein 2	CRISP2
2176	TNF receptor associated factor 2	TRAF2
2177	transient receptor potential cation channel subfamily C member 3	TRPC3
2178	microRNA 650	MIR650
2179	microRNA 655	MIR655
2180	tumor susceptibility 101	TSG101
2181	testis specific protein Y-linked 1	TSPY1
2182	pleckstrin homology like domain family A member 2	PHLDA2
2183	TTK protein kinase	TTK
2184	POTE ankyrin domain family member F	POTEF
2185	SPANX family member A2	SPANXA2
2186	OIP5 antisense RNA 1	OIP5-AS1
2187	ubiquitin conjugating enzyme E2 V1	UBE2V1
2188	ubiquitin C-terminal hydrolase L1	UCHL1
2189	UDP glycosyltransferase 8	UGT8
2190	upstream transcription factor 2, c-fos interacting	USF2
2191	vascular cell adhesion molecule 1	VCAM1
2192	valosin containing protein	VCP
2193	voltage dependent anion channel 1	VDAC1
2194	vitamin D receptor	VDR
2195	VGF nerve growth factor inducible	VGF
2196	von Hippel-Lindau tumor suppressor	VHL
2197	von Willebrand factor	VWF
2198	eukaryotic translation initiation factor 4H	EIF4H
2199	WEE1 G2 checkpoint kinase	WEE1
2200	Wnt family member 1	WNT1
2201	Wnt family member 7A	WNT7A
2202	WRN RecQ like helicase	WRN
2203	WT1 transcription factor	WT1
2204	X-box binding protein 1	XBP1
2205	xanthine dehydrogenase	XDH
2206	X-linked Kx blood group	XK
2207	XPA, DNA damage recognition and repair factor	XPA
2208	YY1 transcription factor	YY1

2209	tyrosine 3-monooxygenase/tryptophan 5-monooxygenase activation protein beta	YWHAB
2210	tyrosine 3-monooxygenase/tryptophan 5-monooxygenase activation protein epsilon	YWHAE
2211	tyrosine 3-monooxygenase/tryptophan 5-monooxygenase activation protein gamma	YWHAG
2212	carbonic anhydrase 8	CA8
2213	carbonic anhydrase 9	CA9
2214	microRNA 767	MIR767
2215	carbonic anhydrase 11	CA11
2216	tripartite motif containing 25	TRIM25
2217	carbonic anhydrase 12	CA12
2218	zinc finger protein 185 with LIM domain	ZNF185
2219	calcium voltage-gated channel subunit alpha1 C	CACNA1C
2220	PR/SET domain 2	PRDM2
2221	interleukin 1 receptor type 2	IL1R2
2222	CUE domain containing 2	CUEDC2
2223	AHNAK nucleoprotein	AHNAK
2224	fatty acid 2-hydroxylase	FA2H
2225	TNFAIP3 interacting protein 2	TNIP2
2226	fibronectin type III and SPRY domain containing 1	FSD1
2227	Laryngeal adductor paralysis	LAP
2228	transcription factor EB	TFEB
2229	chondroitin polymerizing factor	CHPF
2230	mitochondrial E3 ubiquitin protein ligase 1	MUL1
2231	polypeptide N-acetylgalactosaminyltransferase 14	GALNT14
2232	suppressor of variegation 3-9 homolog 2	SUV39H2
2233	zinc finger homeobox 4	ZFHX4
2234	mitochondrial transcription termination factor 1	MTERF1
2235	myosin heavy chain 14	MYH14
2236	agmatinase	AGMAT
2237	transmembrane channel like 5	TMC5
2238	Rho guanine nucleotide exchange factor 5	ARHGEF5
2239	long intergenic non-protein coding RNA 115	LINC00115
2240	lysophosphatidylcholine acyltransferase 1	LPCAT1
2241	long intergenic non-protein coding RNA 472	LINC00472
2242	dehydrodolichyl diphosphate synthase subunit	DHDDS
2243	ADP ribosylation factor like GTPase 14	ARL14
2244	CST telomere replication complex component 1	CTC1
2245	LIM homeobox 3	LHX3
2246	WD repeat and coiled coil containing	WDCP
2247	solute carrier family 25 member 16	SLC25A16
2248	TRAF3 interacting protein 3	TRAF3IP3
2249	ADAM metallopeptidase domain 12	ADAM12

2250	SRC kinase signaling inhibitor 1	SRCIN1
2251	inter-alpha-trypsin inhibitor heavy chain 5	ITIH5
2252	THAP domain containing 7	THAP7
2253	ASXL transcriptional regulator 3	ASXL3
2254	zinc finger protein 436	ZNF436
2255	lysine methyltransferase 2D	KMT2D
2256	Wnt family member 5B	WNT5B
2257	calreticulin	CALR
2258	calumenin	CALU
2259	solute carrier family 7 member 5	SLC7A5
2260	solute carrier family 38 member 1	SLC38A1
2261	tripartite motif containing 11	TRIM11
2262	diaphanous related formin 3	DIAPH3
2263	cyclin L2	CCNL2
2264	vacuole membrane protein 1	VMP1
2265	sprouty RTK signaling antagonist 4	SPRY4
2266	calnexin	CANX
2267	calcyphosine	CAPS
2268	eosinophil peroxidase	EPX
2269	AT-rich interaction domain 1A	ARID1A
2270	calpastatin	CAST
2271	cell division cycle 7	CDC7
2272	caspase 1	CASP1
2273	SH3 domain binding glutamate rich protein like 3	SH3BGRL3
2274	abhydrolase domain containing 11	ABHD11
2275	DEAD-box helicase 59	DDX59
2276	SRY-box transcription factor 7	SOX7
2277	zinc finger MIZ-type containing 2	ZMIZ2
2278	loss of heterozygosity, 19, chromosomal region 1	LOH19CR1
2279	fibronectin type III and SPRY domain containing 1 like	FSD1L
2280	ATPase family AAA domain containing 3B	ATAD3B
2281	cell division cycle associated 7	CDCA7
2282	coiled-coil domain containing 8	CCDC8
2283	regenerating family member 4	REG4
2284	elastin microfibril interfacer 2	EMILIN2
2285	transgelin 2	TAGLN2
2286	MINDY lysine 48 deubiquitinase 4	MINDY4
2287	small nucleolar RNA host gene 3	SNHG3
2288	serine/threonine kinase 24	STK24
2289	GINS complex subunit 4	GINS4
2290	BRMS1 like transcriptional repressor	BRMS1L
2291	AKT1 substrate 1	AKT1S1
2292	reversion inducing cysteine rich protein with kazal motifs	RECK
2293	sterol O-acyltransferase 2	SOAT2

2294	caveolae associated protein 2	CAVIN2
2295	ECRG4 augurin precursor	ECRG4
2296	dual specificity tyrosine phosphorylation regulated kinase 2	DYRK2
2297	zinc finger protein 512	ZNF512
2298	cullin 4B	CUL4B
2299	minichromosome maintenance 8 homologous recombination repair factor	MCM8
2300	cullin 3	CUL3
2301	small nucleolar RNA, C/D box 35B	SNORD35B
2302	microtubule associated protein 1 light chain 3 alpha	MAP1LC3A
2303	calcium sensing receptor	CASR
2304	interleukin 1 family member 10	IL1F10
2305	spermatogenic leucine zipper 1	SPZ1
2306	long intergenic non-protein coding RNA 852	LINC00852
2307	hes family bHLH transcription factor 7	HES7
2308	catalase	CAT
2309	AFAP1 antisense RNA 1	AFAP1-AS1
2310	semaphorin 7A (John Milton Hagen blood group)	SEMA7A
2311	lamin B2	LMNB2
2312	atonal bHLH transcription factor 8	ATOH8
2313	protein phosphatase, Mg2+/Mn2+ dependent 1D	PPM1D
2314	tensin 4	TNS4
2315	ubiquitin associated and SH3 domain containing B	UBASH3B
2316	small nucleolar RNA host gene 12	SNHG12
2317	phosphoinositide-3-kinase regulatory subunit 3	PIK3R3
2318	poly(ADP-ribose) glycohydrolase	PARG
2319	integrin subunit alpha 8	ITGA8
2320	interferon induced transmembrane protein 1	IFITM1
2321	ATP binding cassette subfamily C member 11	ABCC11
2322	myosin light chain kinase 2	MYLK2
2323	small nucleolar RNA, C/D box 14B	SNORD14B
2324	small nucleolar RNA, C/D box 14C	SNORD14C
2325	small nucleolar RNA, C/D box 14D	SNORD14D
2326	small nucleolar RNA, C/D box 14E	SNORD14E
2327	tankyrase 1 binding protein 1	TNKS1BP1
2328	basic helix-loop-helix family member e40	BHLHE40
2329	protein inhibitor of activated STAT 1	PIAS1
2330	cyclin dependent kinase 10	CDK10
2331	RUNX family transcription factor 2	RUNX2
2332	RuvB like AAA ATPase 1	RUVBL1
2333	RUNX family transcription factor 1	RUNX1
2334	eukaryotic translation initiation factor 3 subunit A	EIF3A
2335	eukaryotic translation initiation factor 3 subunit F	EIF3F
2336	embryonic ectoderm development	EED
2337	TNF superfamily member 13	TNFSF13

2338	TNF receptor superfamily member 6b	TNFRSF6B
2339	regulator of G protein signaling 11	RGS11
2340	TNF receptor superfamily member 10b	TNFRSF10B
2341	IQ motif containing GTPase activating protein 1	IQGAP1
2342	neuropilin 2	NRP2
2343	suppressor of cytokine signaling 2	SOCS2
2344	CASP8 and FADD like apoptosis regulator	CFLAR
2345	cyclin dependent kinase 5 regulatory subunit 1	CDK5R1
2346	nuclear receptor subfamily 1 group I member 2	NR1I2
2347	apelin	APLN
2348	Rho guanine nucleotide exchange factor 7	ARHGEF7
2349	sequestosome 1	SQSTM1
2350	cyclin A2	CCNA2
2351	cyclin B1	CCNB1
2352	autophagy related 16 like 2	ATG16L2
2353	tripartite motif containing 15	TRIM15
2354	transient receptor potential cation channel subfamily A member 1	TRPA1
2355	LIM domains containing 1	LIMD1
2356	F2R like thrombin or trypsin receptor 3	F2RL3
2357	heparan sulfate 6-O-sulfotransferase 2	HS6ST2
2358	urocortin 2	UCN2
2359	CEA cell adhesion molecule 21	CEACAM21
2360	C-C motif chemokine receptor like 2	CCRL2
2361	semaphorin 5A	SEMA5A
2362	NFS1 cysteine desulfurase	NFS1
2363	3'-phosphoadenosine 5'-phosphosulfate synthase 1	PAPSS1
2364	claudin 6	CLDN6
2365	basic charge Y-linked 2	BPY2
2366	interleukin 33	IL33
2367	ubiquitin specific peptidase 10	USP10
2368	N-myc and STAT interactor	NMI
2369	metastasis associated 1	MTA1
2370	solute carrier family 16 member 4	SLC16A4
2371	solute carrier family 16 member 3	SLC16A3
2372	RCC1 domain containing 1	RCCD1
2373	lysophosphatidic acid receptor 2	LPAR2
2374	oncostatin M receptor	OSMR
2375	Rho/Rac guanine nucleotide exchange factor 2	ARHGEF2
2376	MARVEL domain containing 3	MARVELD3
2377	CD247 molecule	CD247
2378	death effector domain containing	DEDD
2379	aurora kinase B	AURKB
2380	metadherin	MTDH
2381	nucleolar and coiled-body phosphoprotein 1	NOLC1

2382	PTTG1 regulator of sister chromatid separation, securin	PTTG1
2383	interleukin 32	IL32
2384	musculin	MSC
2385	MAFG divergent transcript	MAFG-DT
2386	piwi like RNA-mediated gene silencing 1	PIWIL1
2387	delta/notch like EGF repeat containing	DNER
2388	BPI fold containing family B member 1	BPIFB1
2389	BAF chromatin remodeling complex subunit BCL7B	BCL7B
2390	COPI coat complex subunit beta 2	COPB2
2391	CD9 molecule	CD9
2392	adhesion G protein-coupled receptor G1	ADGRG1
2393	membrane associated ring-CH-type finger 9	MARCHF9
2394	nicotinate phosphoribosyltransferase	NAPRT
2395	ORAI calcium release-activated calcium modulator 3	ORAI3
2396	thyroid hormone receptor interactor 13	TRIP13
2397	thyroid hormone receptor interactor 10	TRIP10
2398	TAO kinase 2	TAOK2
2399	cytochrome c oxidase subunit 5A	COX5A
2400	CD28 molecule	CD28
2401	absent in melanoma 2	AIM2
2402	geranylgeranyl diphosphate synthase 1	GGPS1
2403	autophagy related 5	ATG5
2404	kinesin family member 23	KIF23
2405	CD38 molecule	CD38
2406	BAG cochaperone 3	BAG3
2407	tumor protein p53 inducible protein 3	TP53I3
2408	C-X-C motif chemokine ligand 14	CXCL14
2409	chromodomain helicase DNA binding protein 1 like	CHD1L
2410	C-C motif chemokine ligand 4 like 2	CCL4L2
2411	gamma-aminobutyric acid type B receptor subunit 2	GABBR2
2412	Ras and Rab interactor 1	RIN1
2413	nuclear receptor corepressor 1	NCOR1
2414	nuclear receptor corepressor 2	NCOR2
2415	TNF receptor associated factor 4	TRAF4
2416	ATP binding cassette subfamily G member 1	ABCG1
2417	chloride channel accessory 2	CLCA2
2418	mortality factor 4 like 2	MORF4L2
2419	SH3 and PX domains 2A	SH3PXD2A
2420	GRIP and coiled-coil domain containing 2	GCC2
2421	family with sequence similarity 53 member B	FAM53B
2422	basic leucine zipper and W2 domains 1	BZW1
2423	CD69 molecule	CD69
2424	extra spindle pole bodies like 1, separase	ESPL1
2425	ST18 C2H2C-type zinc finger transcription factor	ST18

2426	dedicator of cytokinesis 4	DOCK4
2427	CD151 molecule (Raph blood group)	CD151
2428	MTSS I-BAR domain containing 1	MTSS1
2429	nucleoporin 58	NUP58
2430	spermatogenesis associated 2	SPATA2
2431	SPT7 like, STAGA complex subunit gamma	SUPT7L
2432	IQ motif and Sec7 domain ArfGEF 1	IQSEC1
2433	kinesin family member 14	KIF14
2434	cell division cycle 25A	CDC25A
2435	purinergic receptor P2Y14	P2RY14
2436	cell division cycle 25C	CDC25C
2437	nuclear receptor subfamily 1 group I member 3	NR1I3
2438	synthesis of cytochrome C oxidase 2	SCO2

Table S3. String interactions short of PPI.

node1	node2	combined score	node1	node2	combined score
ABCB1	NR3C1	0.4	DNMT3B	GSTP1	0.468
ABCB1	KDR	0.404	DNMT3B	PARP1	0.486
ABCB1	EGFR	0.694	EDNRA	MMP2	0.418
ABCB1	CASP3	0.566	EDNRA	EGFR	0.936
ABCB1	TYMS	0.618	EDNRA	SRC	0.528
ABCB1	CTNNB1	0.528	EDNRA	MAPK8	0.907
ABCB1	SRC	0.407	EGFR	MAPK1	0.962
ABCB1	MAPK8	0.918	EGFR	MMP2	0.707
ABCB1	GSTP1	0.595	EGFR	NR3C1	0.788
ABCB1	DHFR	0.485	EGFR	FOLH1	0.611
ABCB1	AKT1	0.636	EGFR	MDM2	0.87
ACLY	GSR	0.455	EGFR	PDGFRB	0.946
ACLY	FABP4	0.426	EGFR	SELP	0.412
ACLY	AKT1	0.64	EGFR	KDR	0.629
AKT1	MAPK1	0.895	EGFR	IGF1R	0.808
AKT1	MMP2	0.772	EGFR	WEE1	0.429
AKT1	GSR	0.471	EGFR	MMP10	0.435
AKT1	NR3C1	0.676	EGFR	FGFR1	0.465
AKT1	FABP4	0.524	EGFR	MMP1	0.535
AKT1	MDM2	0.998	EGFR	TYMS	0.606
AKT1	MMP13	0.521	EGFR	PARP1	0.644
AKT1	PDGFRB	0.937	EGFR	GSTP1	0.699
AKT1	CREBBP	0.978	EGFR	MAPK8	0.731
AKT1	SELP	0.521	EGFR	MMP9	0.733
AKT1	KDR	0.441	EGFR	MMP14	0.745
AKT1	IGF1R	0.578	EGFR	MET	0.946
AKT1	EGFR	0.794	EGFR	SRC	0.995

AKT1	MMP14	0.598	EGLN1	SRC	0.431
AKT1	CASP3	0.935	FGFR1	MAPK1	0.923
AKT1	EDNRA	0.937	FGFR1	MMP2	0.514
AKT1	TYMS	0.419	FGFR1	MDM2	0.47
AKT1	IMPDH2	0.451	FGFR1	PDGFRB	0.45
AKT1	MMP1	0.56	FGFR1	KDR	0.529
AKT1	DNMT3B	0.414	FGFR1	MET	0.45
AKT1	CTNNB1	0.996	FGFR1	SRC	0.544
AKT1	EGLN1	0.549	FOLH1	MDM2	0.475
AKT1	PARP1	0.666	GSR	GSTP1	0.85
AKT1	MMP9	0.815	GSR	GSTM2	0.863
AKT1	SRC	0.985	GSTM2	SRC	0.453
AKT1	ALOX5	0.456	GSTM2	GSTP1	0.704
AKT1	MAPK8	0.642	GSTP1	TYMS	0.603
AKT1	GSTP1	0.405	GSTP1	MAPK8	0.995
AKT1	FGFR1	0.564	IGF1R	MAPK1	0.935
AKT1	WEE1	0.509	IGF1R	MMP2	0.499
AKT1	CASP1	0.586	IGF1R	MDM2	0.988
ALOX5	MMP2	0.503	IGF1R	KDR	0.41
ALOX5	SELP	0.403	IGF1R	MMP9	0.504
ALOX5	MMP9	0.526	IGF1R	MAPK8	0.524
CASP1	EGFR	0.465	IGF1R	SRC	0.959
CASP1	CASP3	0.928	IMPDH2	TYMS	0.572
CASP1	PARP1	0.572	KDR	MAPK1	0.415
CASP1	MMP9	0.531	KDR	MMP2	0.639
CASP1	MAPK8	0.42	KDR	PDGFRB	0.57
CASP1	ERN1	0.419	KDR	SELP	0.469
CASP3	MAPK1	0.914	KDR	MMP1	0.466
CASP3	MMP2	0.681	KDR	MMP14	0.472
CASP3	GSR	0.603	KDR	MET	0.702
CASP3	NR3C1	0.403	KDR	MMP9	0.76
CASP3	MDM2	0.965	KDR	SRC	0.992
CASP3	MMP13	0.522	MAPK1	MDM2	0.417
CASP3	PDGFRB	0.457	MAPK1	MMP9	0.424
CASP3	SELP	0.408	MAPK1	WEE1	0.428
CASP3	KDR	0.605	MAPK1	PARP1	0.741
CASP3	IGF1R	0.617	MAPK1	MET	0.925
CASP3	EGFR	0.759	MAPK1	MAPK8	0.931
CASP3	MMP10	0.443	MAPK1	NR3C1	0.943
CASP3	FGFR1	0.411	MAPK1	SRC	0.956
CASP3	TYMS	0.428	MAPK8	MMP2	0.511
CASP3	MMP1	0.472	MAPK8	NR3C1	0.94
CASP3	WEE1	0.582	MAPK8	MDM2	0.407
CASP3	SRC	0.689	MAPK8	MET	0.92

CASP3	ERN1	0.689	MAPK8	MMP1	0.408
CASP3	MMP9	0.772	MAPK8	MMP9	0.569
CASP3	MAPK8	0.785	MAPK8	SRC	0.947
CASP3	MET	0.815	MDM2	NR3C1	0.532
CASP3	CTNNB1	0.99	MDM2	MMP9	0.403
CASP3	PARP1	0.998	MDM2	PDGFRB	0.424
CREBBP	MAPK1	0.675	MDM2	MET	0.433
CREBBP	NR3C1	0.993	MDM2	PARP1	0.46
CREBBP	FABP4	0.914	MDM2	WEE1	0.502
CREBBP	MDM2	0.997	MDM2	SRC	0.865
CREBBP	DNMT3B	0.41	MET	MMP2	0.531
CREBBP	EGFR	0.486	MET	PDGFRB	0.41
CREBBP	FGFR1	0.898	MET	MMP14	0.421
CREBBP	SRC	0.9	MET	MMP1	0.403
CREBBP	PARP1	0.967	MET	MMP9	0.57
CREBBP	CTNNB1	0.999	MET	SRC	0.982
CTNNB1	MAPK1	0.633	MMP1	MMP2	0.921
CTNNB1	MMP2	0.696	MMP1	MMP13	0.682
CTNNB1	NR3C1	0.465	MMP1	MMP10	0.936
CTNNB1	FABP4	0.404	MMP1	SRC	0.441
CTNNB1	MDM2	0.64	MMP1	MMP9	0.961
CTNNB1	MMP13	0.578	MMP10	MMP2	0.692
CTNNB1	PDGFRB	0.539	MMP10	MMP13	0.685
CTNNB1	KDR	0.998	MMP10	MMP9	0.966
CTNNB1	IGF1R	0.989	MMP12	MMP9	0.724
CTNNB1	EGFR	0.999	MMP13	MMP9	0.436
CTNNB1	MMP10	0.492	MMP13	MMP14	0.931
CTNNB1	MMP14	0.593	MMP14	MMP2	0.923
CTNNB1	EDNRA	0.442	MMP14	PDGFRB	0.645
CTNNB1	TYMS	0.489	MMP14	SRC	0.929
CTNNB1	MET	0.994	MMP2	PARP1	0.451
CTNNB1	MMP1	0.509	MMP2	SELP	0.459
CTNNB1	DNMT3B	0.451	MMP2	PDGFRB	0.591
CTNNB1	DHFR	0.53	MMP2	MMP9	0.908
CTNNB1	PARP1	0.656	MMP2	SRC	0.96
CTNNB1	MMP9	0.763	MMP9	PDGFRB	0.482
CTNNB1	MAPK8	0.817	MMP9	SELP	0.579
CTNNB1	FGFR1	0.99	MMP9	SRC	0.97
CTNNB1	SRC	0.999	NR3C1	SRC	0.517
DHFR	MDM2	0.699	PARP1	SRC	0.4
DHFR	EGFR	0.43	PARP1	WEE1	0.599
DHFR	TYMS	0.999	PDGFRB	SRC	0.991
DNMT3B	TYMS	0.415	SELP	SRC	0.479

Table S4. The results of Molecular docking.

Target	-CDOCKER ENERGY	-CDOCKER INTERACTION ENERGY	ChiFlex Energy	LibDockScore
AKT1	-2.46764	49.1404	-25.952	81.8119
DHFR	0.638308	47.0942	-18.446	103.171
TYMS	25.9965	72.9033	1.763	96.2157
