# Supplementary material for: Diosbulbin C, a novel active ingredient in Dioscorea bulbifera L. extract, inhibits lung cancer cell proliferation by inducing G0/G1 phase cell cycle arrest
Source: BMC Complement Med Ther. 2023 Dec 4;23:436. doi: 10.1186/s12906-023-04245-9 (PMC10694954; doi:10.1186/s12906-023-04245-9)
Supplement: Supplementary file 2 — Additional file 2. [file 12906_2023_4245_MOESM2_ESM.pdf]

Diosbulbin C, a novel active ingredient in *Dioscorea bulbifera* L. extract, inhibits lung cancer cell proliferation by inducing G0/G1 phase cell cycle arrest

Zhiyu Zhu[1], Yanfen Liu[1], Jiangping Zeng[1], Shuyi Ren[1], Lu Wei[1], Fei Wang[1], Xiaoyu Sun[1], Yufei Huang[1], Haiyang Jiang[1], Xinbing Sui[1,2], Weiwei Jin[2*], Lijun Jin[3*], Xueni Sun[1,2*]

[1] School of Pharmacy, Key Laboratory of Elemene Class Anti-Cancer Chinese Medicines; Engineering Laboratory of Development and Application of Traditional Chinese Medicines; Collaborative Innovation Center of Traditional Chinese Medicines of Zhejiang Province, Hangzhou Normal University, Hangzhou, Zhejiang 311121, China

[2] Department of Gastrointestinal & Pancreatic Surgery, Key Laboratory of Gastroenterology of Zhejiang Province, Zhejiang Provincial People's Hospital, People's Hospital of Hangzhou Medical College, Hangzhou, Zhejiang 310014, China

[3] Department of Traditional Chinese Medicine, Hangzhou Shangcheng District People's Hospital, Hangzhou, China

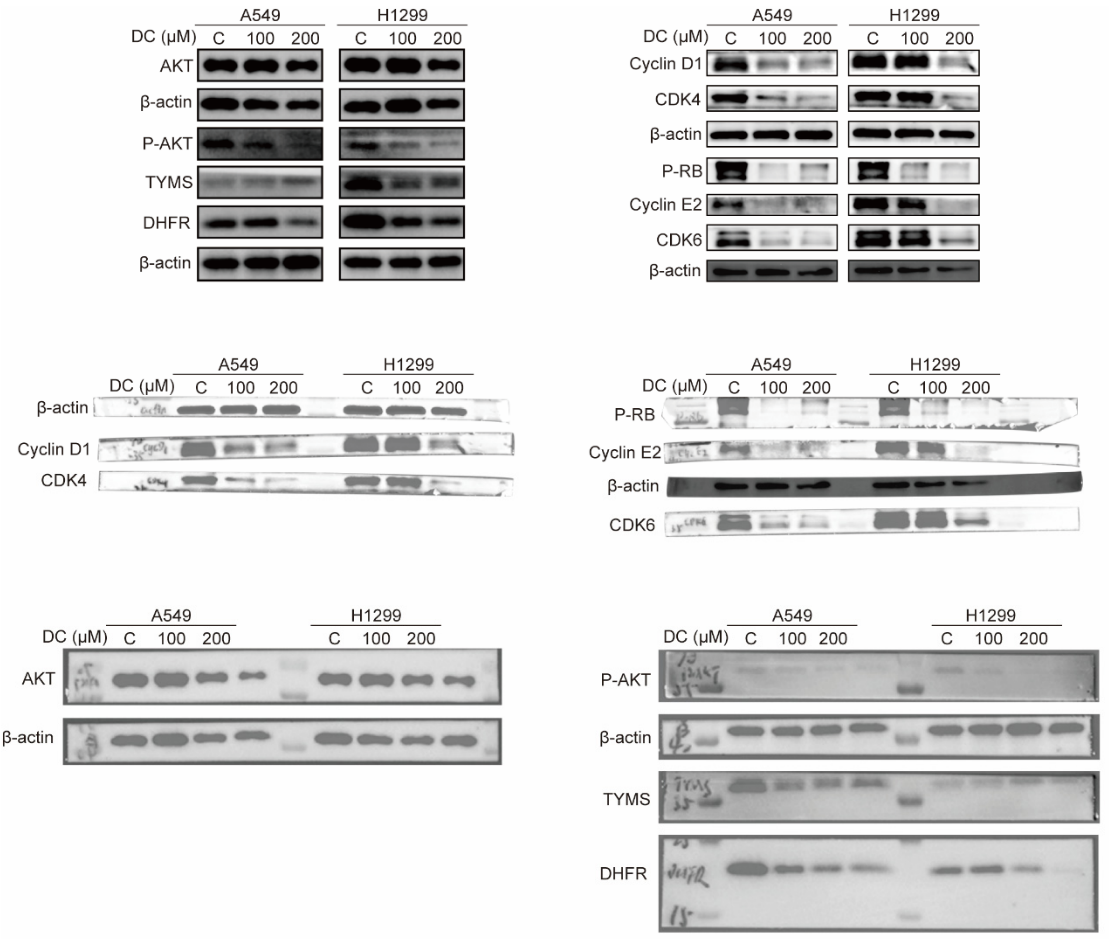

AKT, DHFR, TYMS, β-actin, p-RB, Cyclin D1, Cyclin E2, CDK4, CDK6 original blots of A549 and H1299 cells are shown. All the blots have been processed by photoshop.
